# Facile synthetic approach towards vasorelaxant active 4-hydroxyquinazoline-4-carboxamides†

**DOI:** 10.1039/c9ra04321g

**Published:** 2019-09-10

**Authors:** Marian N. Aziz, Siva S. Panda, ElSayed M. Shalaby, Nehmedo G. Fawzy, Adel S. Girgis

**Affiliations:** Department of Pesticide Chemistry, National Research Centre Dokki Giza 12622 Egypt girgisas10@yahoo.com; Department of Chemistry & Physics, Augusta University Augusta GA 30912 USA; X-Ray Crystallography Lab, Physics Division, National Research Centre Dokki Giza 12622 Egypt

## Abstract

A Facile synthetic approach is reported towards 4-hydroxyquinazoline-4-carboxamides 13a–i through ring expansion of 2,3-dioxoindoline-1-carboxamides 10a–c during secondary amine 11a–d nucleophilic reaction. Single crystal X-ray studies of 10c and 13d support the structures. Some of the synthesized quinazolinecarboxamides 13 show promising vasorelaxant properties with potency higher than that of Doxazosin through the pre-contracted (norepinephrine hydrochloride) rat aorta standard bioassay. Good molecular models (2D-QSAR, pharmacophore) describe the biological observations.

## Abstract 2

A set of vasorelaxant active 4-hydroxyquinazoline-4-carboxamides was obtained in a novel synthetic approach *via* secondary amine nucleophilic reaction with 2,3-dioxoindoline-1-carboxamides.

## Graphical abstract

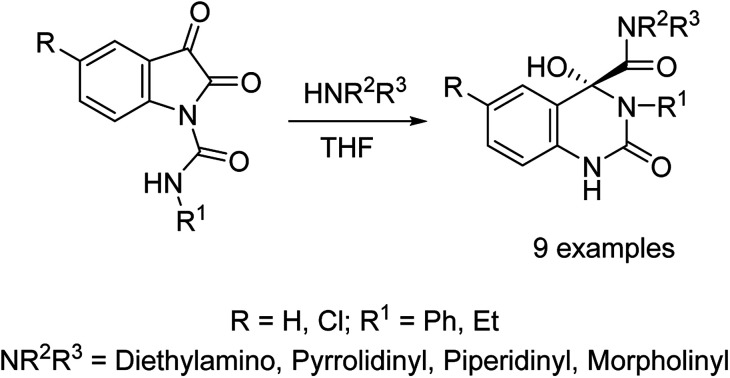

## Introduction

**Fig. 1:**
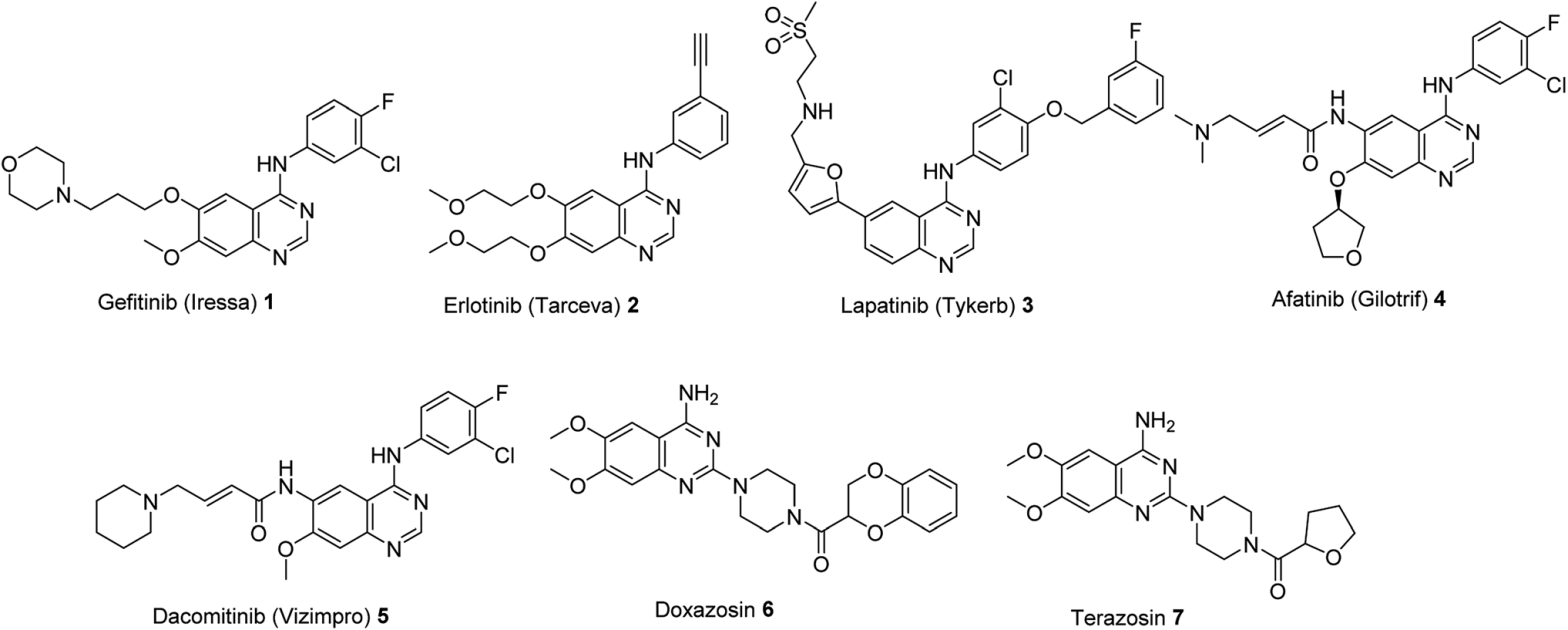
Quinazoline-based antitumor and antihypertensive drugs.

Quinazoline is an important heterocyclic system that occupies a striking position in medicinal chemistry due to the diverse biological and/or pharmacological properties associated with its derivatives.^1–6^ For example, Gefitinib (Iressa) 1 is a drug clinically approved by the FDA for treatment of non-small cell lung cancer (NSCLC, on May 5, 2003) (Fig. 1). It is a tyrosine kinase inhibitor accessible for patients with metastatic NSCLC.^7,8^ Erlotinib (Tarceva) 2 is also a kinase inhibitor approved by the FDA (Nov. 18, 2004) for locally advanced or metastatic NSCLC and pancreatic cancers.^9,10^ Lapatinib (Tykerb) 3 is approved by the FDA (Mar. 13, 2007) for treatment of advanced or metastatic breast cancer.^11,12^ However, due to the drug resistance observed by the targeted first generation reversible EGFR (epidermal growth factor receptor) drugs 1–3, by many patients, the second generation irreversible EGFR inhibitors were developed.^13^ Of which, Afatinib (Gilotrif) 4 and Dacomitinib (Vizimpro) 5 were approved for treatment of metastatic NSCLC (FDA on July 12, 2013 and Sept. 27, 2018, respectively).^14–17^

Additionally, Doxazosin 6 and Terazosin 7 are well known drugs for hypertension.^18^ Hypertension is one of the cardiovascular diseases which are the first cause of human death globally (approximately 17.9 millions representing 31% of global deaths in 2016 according to WHO “World Health Organization”^19,20^). Heart attack and stroke are usually associated with hypertension.^21^ Many α_1_-adrenergic receptor (α_1_-AR) antagonists are clinically useful drugs for vascular smooth muscle relaxation (vasodilator) such as Doxazosin 6 and Terazosin 7 but associate with severe side effects.^22^ The high mortality factor of hypertension and serious side effects of the clinically known drugs dimensioned their applications, directed the research efforts for developing novel effective hits/leads. The recent publications describing diverse biological properties of quinazoline containing-compounds as antibacterial,^23^ antiviral,^24^ antifungal,^25^ antiplasmodial,^26^ anti-inflammatory,^27^ cholinesterase,^28,29^ and monoamine oxidase inhibitors^30^ also prompted the present work.

**Scheme 1:**
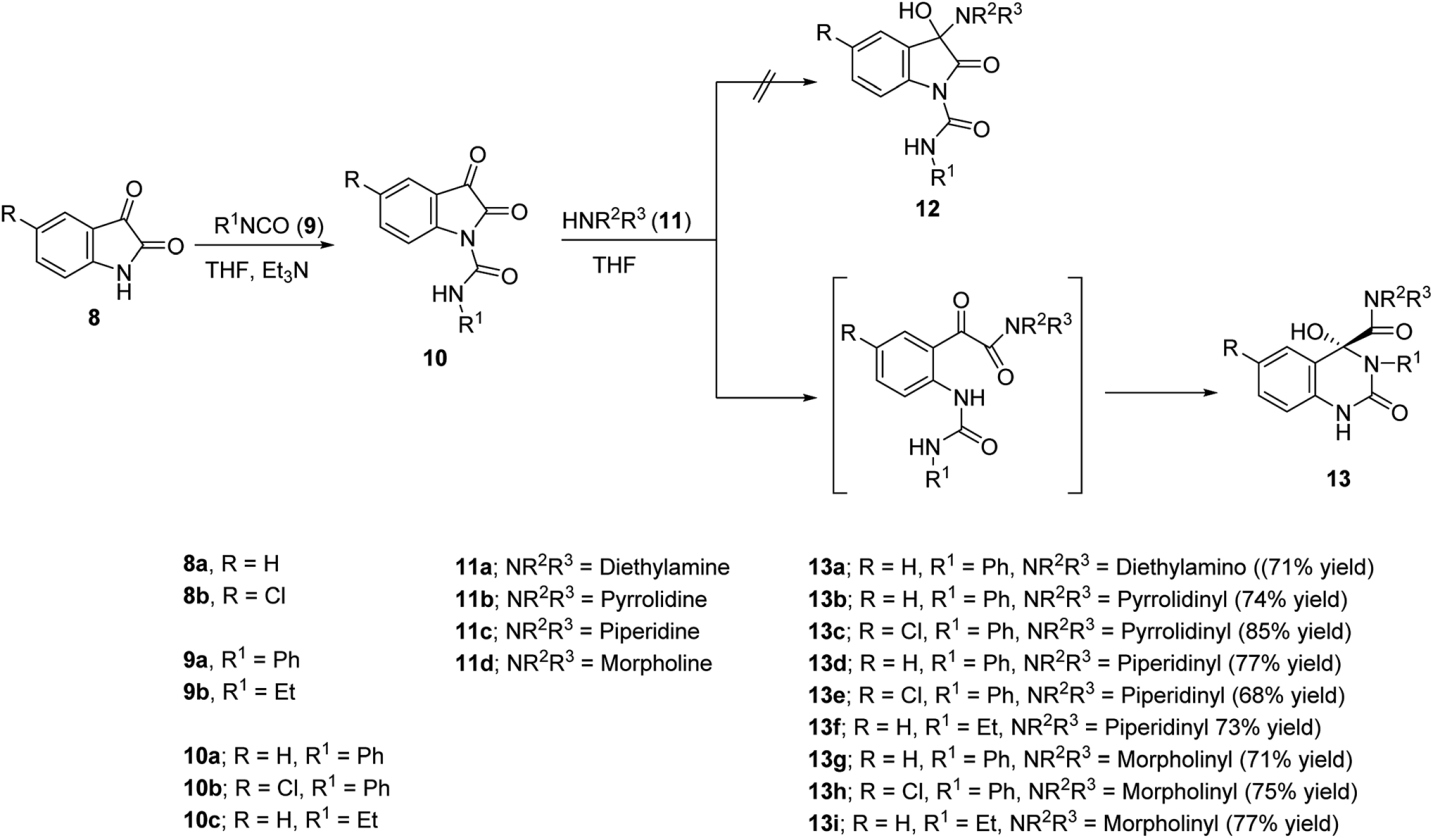
Synthetic route towards 4-hydroxyquinazoline-4-carboxamides 13a–i.

The present study is directed towards construction of novel quinazoline-based analogues and investigation their vasorelaxant properties. The amino group located at the 4-position of the antihypertensive active drugs 6 and 7 were replaced by a hydroxy group due to the common chemical properties of the two functions. The alicyclic-amino ring is also attached to the quinazolinyl C-4 forming an amidic linkage (Scheme 1). Many synthetic pathways were developed for construction of quinazoline-containing compounds including *m*-chloroperbenzoic acid oxidative rearrangement of 4-imino-(1*H*,4*H*)-3,1-benzoxazin-2-ones and reaction of isatins with arylamines in the presence of hydrogen peroxide as an oxidant.^31^ Previous publications described the ring opening of isatins under the effect of water, alcohols, amines,^32,33^ urea,^34^ hydrazines^33,35^ and guanidine.^34,36^ The present study describes a facile synthetic approach *via* ring expansion of isatin-1-carboxamides during secondary amine nucleophilic attack.

## Results and discussion

### Chemistry

**Fig. 2:**
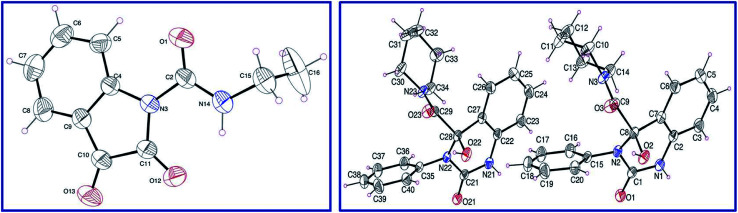
ORTEP views of compounds 10c (left) and 13d (right) with the atom-numbering scheme. H atoms are shown as small spheres of arbitrary radii.

Isocyanates 9a,b were subjected to reaction with isatins 8a,b in dry tetrahydrofuran (THF) in the presence of sufficient amount of triethylamine at 0 °C affording the corresponding 2,3-dioxoindoline-1-carboxamides 10a–c in good yields which were used in the next reaction without any further purification. *N*-Ethyl-2,3-dioxoindoline-1-carboxamide (10c) could be isolated in good microcrystallized form accessible for single crystal X-ray studies, which add good support for the structure (Fig. 2).

Reaction of 10a–c with secondary amines 11a–d in dry THF led to ring expansion affording directly the 4-hydroxyquinazoline-4-carboxamides 13a–i and not the expected 3-hydroxy-2-oxoindoline-1-carboxamides 12. IR spectrum of 13a (an example of the synthesized analogues) reveals the carbonyl groups as a broad signal at *ν* = 1670 cm^−1^. ^1^H-NMR spectrum of 13a reveals the triplet and quartet signals of the ethyl groups at *δ*_H_ = 1.03 and 2.74 ppm, respectively, which are also well observed at *δ*_C_ = 10.8, 18.5; 41.1, 56.0, respectively in the ^13^C-NMR spectrum. The quinazolinyl C-2 and amidic carbonyls are shown at *δ*_C_ = 151.9, 172.4 ppm, respectively. Meanwhile, the characteristic quinazolinyl C-4 is exhibited at *δ*_C_ = 87.1 ppm (spectral charts are exhibited in ESI Fig. S1–S30,† overlapping of few carbon signals in the ^13^C-NMR spectral data of compounds 13g,h is observed). Single crystal X-ray studies of 13d support the chemical/stereochemical structure assigned (Fig. 2). The reaction is assumed to take place through amine 11 nucleophilic attack at the indolyl C-2 giving rise to five-membered ring opening followed by re-cyclization with ring expansion to the six-membered quinazoline heterocycle (Scheme 1).

### X-ray studies

ORTEP views of compounds 10c and 13d are revealed in Fig. 2. Compound 10c is crystallized in the monoclinic space group *C*2/*c* while, compound 13d is crystallized in the monoclinic space group *P*2_1_/*n* (ESI Table S1†). The main constituent of 10c is the indolyl heterocycle and the main constituents of 13d are quinazolinyl, piperidinyl and phenyl rings. Compound 10c exhibits one molecule per asymmetric unit cell and four molecules per unit cell. However, compound 13d shows two molecules per asymmetric unit cell and eight molecules per unit cell. In 10c, the ethyl group is nearly perpendicular to the plane containing the main constituent. Meanwhile, the piperidinyl ring of compound 13d exhibits a chair conformation and the phenyl as well as quinazolinyl heterocycle are nearly planar. Generally, the geometrical parameters of compounds 10c and 13d (ESI Tables S2 and S3†) are comparable to structures having similar constituents.^37^ Different sets of intermolecular hydrogen-bonding interactions stabilize the crystal structure of the studied compounds and led to the formation of supramolecular assemblies (ESI Table S4, Fig. S31 and S32†).

### Vasodilation studies

The standard pre-contracted (norepinephrine hydrochloride) rat aorta technique was utilized for determination the vasodilation properties of the synthesized compounds 10c, 13a–i and compared with Doxazosin (α_1_-AR antagonist).^38^ From the exhibited data (Table 1, ESI Fig. S33 and S34†) it can be concluded that, many of the synthesized quinazolines are vasorelaxant active agents with efficacy comparable to that of Doxazosin. Compound 13h seems superior among all the tested analogues with 2.2 folds potency relative to the standard reference (IC_50_ = 158, 348 μM for 13h and Doxazosin, respectively). Compounds 13e also shows remarkable vasorelaxant properties (about 139% potency of Doxazosin with IC_50_ = 250 μM). Promising smooth muscle relaxation is also shown by compounds 13a,d,f,g (IC_50_ = 298–332 μM). *N*-Ethyl-2,3-dioxoindoline-1-carboxamide (10c) is also a promising vasorelaxant active agent (1.47 folds efficacy relative to the standard drug).

**Table 1:** Vasorelaxant properties of 10c, 13a–i and Doxazosin

Entry	Compd.	R	R^1^	NR^2^/R^3^	IC_50_, μM
1	10c	H	Et	—	236
2	13a	H	Ph	NEt_2_	302
3	13b	H	Ph	Pyrrolidinyl	415
4	13c	Cl	Ph	Pyrrolidinyl	392
5	13d	H	Ph	Piperidinyl	332
6	13e	Cl	Ph	Piperidinyl	250
7	13f	H	Et	Piperidinyl	305
8	13g	H	Ph	Morpholinyl	298
9	13h	Cl	Ph	Morpholinyl	158
10	13i	H	Et	Morpholinyl	416
11	Doxazosin	—	—	—	348

Based on the observed vasodilation properties few SAR (structure–activity relationship) rules can be stated. The chlorine substituent attached at the quinazolinyl C-6 is an important factor for enhancing the biological properties observed as exhibited in pairs 13b/13c, 13d/13e and 13g/13h. The alicyclic-amino ring of the amidic function is also a controlling parameter for biological activity. The importance of the amino ring for vasodilation properties enhancement can be arranged in the following order morpholinyl > piperidinyl > pyrrolidinyl (compound 13f is an exception).

### Molecular modeling studies

#### 2D-QSAR study

**Fig. 3:**
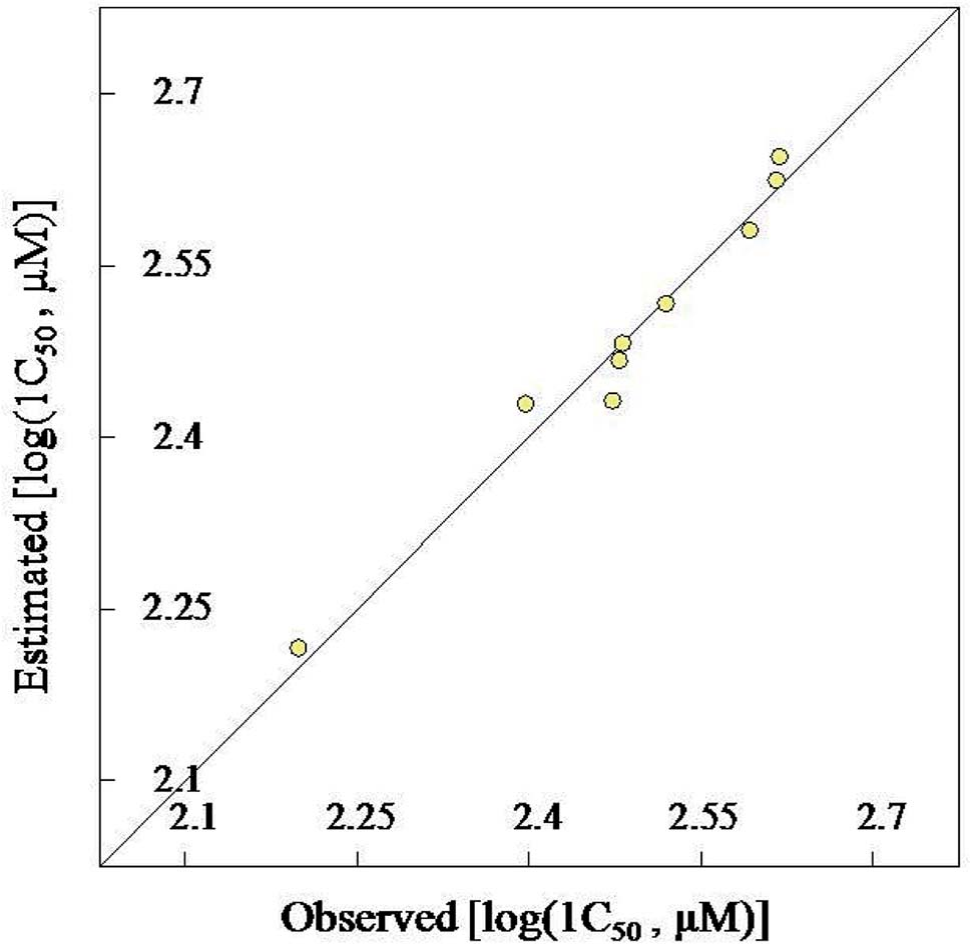
BMLR-QSAR model plot of correlation representing the observed *versus* predicted [log(IC_50_, μM)] values for the synthesized vasorelaxant quinazoline (13a–i).

QSAR (quantitative structure–activity relationship) study is a widely used computational technique for explaining the biological observations and better understanding the parameters optimizing properties.^39^ Two descriptor QSAR model was obtained by CODESSA-Pro for the vasorelaxant quinazoline-4-carboxamides 13a–i (*R*^2^ = 0.970, ESI Table S5†). Explanation/calculation of the QSAR descriptors was mentioned in the ESI.† Goodness of the QSAR model is supported by the leave one-out and leave many-out (up to 20% of the compounds used in the study) coefficient values relative to the original QSAR coefficient value (*R*^2^ = 0.970, *R*^2^cvOO = 0.905, *R*^2^cvMO = 0.937). The predicted IC_50_ values relative to the experimental are also supporting evidence for the robust of computational model (Fig. 3).

#### 3D-Pharmacophore study

Three chemical features (two hydrophobics “H-1, H-2” and one hydrogen bonding donor “HBD”, ESI Fig. S35 and S36†) are shown by the 3D-pharmacophoric model (Discovery Studio 2.5 software) for the vasorelaxant active quinazoline-4-carboxamides 13a–i.^40^ All the synthesized compounds reveal alignment of the hydroxyl group with the HBD and the amino residue/ring of the amidic linkage with one of the hydrophobic in variable fitness affording diverse estimated biological properties (ESI Table S8†). These observations support the elements of design for the present study and also the assigned SAR mentioned. The high correlations between the estimated and experimental biological properties support the goodness of the 3D-pharmacophoric model.

## Conclusions

4-Hydroxyquinazoline-4-carboxamides 13a–i are synthesized in a facile synthetic approach (yield 68–85%) *via* reaction of 2,3-dioxoindoline-1-carboxamides 10a–c with secondary amines 11a–d in dry THF. Single crystal X-ray studies of 10c and 13d support the chemical structures. Some of the synthesized quinazolines reveal vasorelaxant properties with efficacy comparable to that of Doxazosin especially, compound 13h that exhibits 2.2 folds potency relative to the standard reference. 2D-QSAR model describes the biological observations (*N* = 9, *n* = 2, *R*^2^ = 0.970). Also, the 3D-pharmacophoric model supports the elements of design for the present study.

## Experimental

### Chemistry

Melting points were determined on a capillary point apparatus (Stuart SMP3) equipped with a digital thermometer. IR spectra (KBr) were recorded on a Shimadzu FT-IR 8400S spectrophotometer. Reactions were monitored using thin layer chromatography (TLC) on 0.2 mm silica gel F254 plates (Merck) utilizing various solvents for elution. The chemical structures of the synthesized compounds were characterized by nuclear magnetic resonance spectra (^1^H-NMR, ^13^C-NMR) and determined on a Bruker NMR spectrometer (500 MHz, 125 MHz). ^13^C-NMR spectra are fully decoupled. Chemical shifts were reported in parts per million (ppm) using the deuterated solvent peak or tetramethylsilane as an internal standard.

### Reaction of 8a,b with isocyanates 9a,b

A solution of the corresponding isocyanate 9a,b (5 mmol) in dry THF (5 ml) was added dropwise to a mixture of the appropriate 8a,b (5 mmol) in dry THF (20 ml) containing triethylamine (5.5 mmol), at 0 °C. The reaction was magnetically stirred at the same temperature for 10 h then, stored in the fridge overnight. The separated solid was collected and washed with benzene affording 10a,b which was used without any more purifications. In case of the reaction of 8a and 9b, the separated solid was crystallized from benzene affording 10c as orange microcrystals.

#### *N*-Ethyl-2,3-dioxoindoline-1-carboxamide (10c)

It was obtained from the reaction of 8a and 9b (reaction time 10 h) as orange microcrystals from benzene with mp 159–161 °C and yield 73% (0.80 g). IR: *ν*_max_/cm^−1^ 3337, 1756, 1740, 1701, 1612. ^1^H-NMR (DMSO-*d*_6_) *δ* (ppm): 1.15 (t, *J* = 7.2 Hz, 3H, C*H*_3_CH_2_), 3.30–3.35 (m, 2H, CH_3_C*H*_2_), 7.28 (t, *J* = 7.5 Hz, 1H, arom. H), 7.67 (d, *J* = 7.5 Hz, 1H, arom. H), 7.71 (t, *J* = 7.9 Hz, 1H, arom. H), 8.17 (d, *J* = 8.2 Hz, 1H, arom. H), 8.22 (t, *J* = 5.3 Hz, 1H, NH). ^13^C-NMR (DMSO-*d*_6_) *δ* (ppm): 14.7 (CH_2_*C*H_3_), 34.4 (N*C*H_2_CH_3_), 116.5, 118.9, 124.3, 124.6, 137.5, 148.2 (arom. C), 150.4 (urea CO), 158.9 (indolyl C-2), 180.4 (indolyl C-3). Anal. calcd for C_11_H_10_N_2_O_3_ (218.21): C, 60.55; H, 4.62; N, 12.84. Found: C, 60.76; H, 4.77; N, 12.93.

### Reaction of 10a–c with secondary amines 11a–d

The appropriate secondary amine 11a–d (5.5 mmol) was added dropwise to the magnetically stirred solution of 10a–c (5 mmol) in dry THF (20 ml) at room temperature (20–25 °C) for the appropriate time. The separated solid was collected and crystallized from a suitable solvent affording the corresponding 13a,c,d,g–i. In case of 13b, 13e, and 13f the reaction mixture was evaporated till dryness under reduced pressure. The remaining material was triturated with methanol (5 ml). The separated solid was collected and crystallized from a suitable solvent.

#### *N*,*N*-Diethyl-4-hydroxy-2-oxo-3-phenyl-1,2,3,4-tetrahydroquinazoline-4-carboxamide (13a)

It was obtained from the reaction of 10a and 11a (reaction time 8 h) as colorless microcrystals from methanol with mp 145–147 °C and yield 71% (1.20 g). IR: *ν*_max_/cm^−1^ 3202, 3059, 1670, 1609, 1497, 1412. ^1^H-NMR (DMSO-*d*_6_) *δ* (ppm): 1.03 (t, *J* = 6.9 Hz, 6H, 2 CH_2_*CH*_3_), 2.74 (q, *J* = 6.4 Hz, 4H, 2 *CH*_2_CH_3_), 6.41 (br s, 1H, NH), 6.80–6.85 (m, 2H, arom. H), 7.05 (d, *J* = 7.4 Hz, 1H, arom. H), 7.14 (t, *J* = 7.3 Hz, 1H, arom. H), 7.22–7.27 (m, 5H, arom. H), 9.62 (br s, 1H, OH). ^13^C-NMR (DMSO-*d*_6_) *δ* (ppm): 10.8 (CH_2_*C*H_3_), 18.5 (CH_2_*C*H_3_), 41.1 (N*C*H_2_CH_3_), 56.0 (N*C*H_2_CH_3_), 87.1 (quinazolinyl C-4), 113.0, 120.5, 124.0, 125.7, 126.5, 127.6, 128.1, 130.7, 136.0, 139.3 (arom. C), 151.9 (quinazolinyl C-2), 172.4 (carboxamide CO). Anal. calcd for C_19_H_21_N_3_O_3_ (339.40): C, 67.24; H, 6.24; N, 12.38. Found: C, 67.38; H, 6.30; N, 12.42.

#### 4-Hydroxy-3-phenyl-4-(pyrrolidine-1-carbonyl)-3,4-dihydroquinazolin-2(1*H*)-one (13b)

It was obtained from the reaction of 10a and 11b (reaction time 10 h) as colorless microcrystals from methanol with mp 184–186 °C and yield 74% (1.25 g). IR: *ν*_max_/cm^−1^ 3503, 3402, 1678, 1636, 1605, 1504. ^1^H-NMR (DMSO-*d*_6_) *δ* (ppm): 1.59–1.76 (m, 3H, pyrrolidinyl H), 2.53–2.56 (m, 1H, pyrrolidinyl H), 3.01 (quintet, *J* = 7.0 Hz, 1H, pyrrolidinyl H), 3.18 (d, *J* = 4.8 Hz, 1H, pyrrolidinyl H), 3.35–3.38 (m, 1H, pyrrolidinyl H), 3.48–3.53 (m, 1H, pyrrolidinyl H), 6.70 (s, 1H, NH), 6.93–7.00 (m, 3H, arom. H), 7.24 (d, *J* = 7.4 Hz, 2H, arom. H), 7.30–7.38 (m, 4H, arom. H), 10.22 (s, 1H, OH). ^13^C-NMR (DMSO-*d*_6_) *δ* (ppm): 22.7, 25.8 (pyrrolidinyl C-3/4), 46.5, 47.5 (pyrrolidinyl C-2/5), 85.9 (quinazolinyl C-4), 113.8, 119.8, 121.7, 125.5, 127.3, 128.2, 129.4, 129.5, 135.8, 137.6 (arom. C), 150.9 (quinazolinyl C-2), 166.2 (carboxamide CO). Anal. calcd for C_19_H_19_N_3_O_3_ (337.38): C, 67.64; H, 5.68; N, 12.46. Found: C, 67.81; H, 5.59; N, 12.57.

#### 6-Chloro-4-hydroxy-3-phenyl-4-(pyrrolidine-1-carbonyl)-3,4-dihydroquinazolin-2(1*H*)-one (13c)

It was obtained from the reaction of 10b and 11b (reaction time 6 h) as colorless microcrystals from *n*-butanol with mp 208–210 °C and yield 85% (1.58 g). IR: *ν*_max_/cm^−1^ 3564, 3345, 3210, 1682, 1643, 1605, 1489. ^1^H-NMR (DMSO-*d*_6_) *δ* (ppm): 1.59–1.74 (m, 4H, pyrrolidinyl H), 2.57–2.62 (m, 1H, pyrrolidinyl H), 3.01 (quintet, *J* = 6.7 Hz, 1H, pyrrolidinyl H), 3.33–3.52 (m, 2H, pyrrolidinyl H), 6.91 (s, 1H, NH), 6.92 (d, *J* = 2.1 Hz, 1H, arom. H), 6.98 (d, *J* = 8.7 Hz, 1H, arom. H), 7.22 (d, *J* = 7.4 Hz, 2H, arom. H), 7.31–7.39 (m, 4H, arom. H), 10.25 (s, 1H, OH). ^13^C-NMR (DMSO-*d*_6_) *δ* (ppm): 22.6, 25.9 (pyrrolidinyl C-3/4), 46.6, 47.6 (pyrrolidinyl C-2/5), 86.0 (quinazolinyl C-4), 115.8, 122.0, 124.9, 125.1, 127.4, 128.2, 129.5, 129.6, 134.8, 137.4 (arom. C), 150.6 (quinazolinyl C-2), 165.8 (carboxamide CO). Anal. calcd for C_19_H_18_ClN_3_O_3_ (371.82): C, 61.38; H, 4.88; N, 11.30. Found: C, 61.57; H, 5.00; N, 11.38.

#### 4-Hydroxy-3-phenyl-4-(piperidine-1-carbonyl)-3,4-dihydroquinazolin-2(1*H*)-one (13d)

It was obtained from the reaction of 10a and 11c (reaction time 6 h) as colorless microcrystals from ethanol with mp 176–178 °C and yield 77% (1.35 g). IR: *ν*_max_/cm^−1^ 3387, 1682, 1636, 1605, 1501. ^1^H-NMR (DMSO-*d*_6_) *δ* (ppm): 0.62 (br s, 1H, piperidinyl H), 1.12–1.46 (m, 5H, piperidinyl H), 2.64 (t, *J* = 10.9 Hz, 1H, piperidinyl H), 3.12 (t, *J* = 11.8 Hz, 1H, piperidinyl H), 3.49 (d, *J* = 12.8 Hz, 1H, piperidinyl H), 3.94 (d, *J* = 12.3 Hz, 1H, piperidinyl H), 6.75 (s, 1H, NH), 6.93–7.00 (m, 3H, arom. H), 7.30–7.34 (m, 6H, arom. H), 10.22 (s, 1H, OH). ^13^C-NMR (DMSO-*d*_6_) *δ* (ppm): 23.3, 24.0, 25.1 (piperidinyl C-3/4/5), 44.3, 46.9 (piperidinyl C-2/6), 85.4 (quinazolinyl C-4), 114.2, 120.6, 121.7, 125.6, 127.5, 128.3, 129.5, 129.7, 135.0, 137.8 (arom. C), 150.7 (quinazolinyl C-2), 166.2 (carboxamide CO). Anal. calcd for C_20_H_21_N_3_O_3_ (351.41): C, 68.36; H, 6.02; N, 11.96. Found: C, 68.14; H, 5.84; N, 12.16.

#### 6-Chloro-4-hydroxy-3-phenyl-4-(piperidine-1-carbonyl)-3,4-dihydroquinazolin-2(1*H*)-one (13e)

It was obtained from the reaction of 10b and 11c (reaction time 12 h) as colorless microcrystals from *n*-butanol of mp 180–182 °C and yield 68% (1.30 g). IR: *ν*_max_/cm^−1^ 3314, 3210, 1682, 1643, 1605, 1493. ^1^H-NMR (DMSO-*d*_6_) *δ* (ppm): 0.65–0.77 (m, 1H, piperidinyl H), 1.22–1.53 (m, 5H, piperidinyl H), 2.69 (t, *J* = 11.0 Hz, 1H, piperidinyl H), 3.16 (t, *J* = 11.4 Hz, 1H, piperidinyl H), 3.48 (d, *J* = 13.4 Hz, 1H, piperidinyl H), 3.93 (d, *J* = 12.5 Hz, 1H, piperidinyl H), 6.90 (br s, 2H, NH + arom. H), 7.01 (d, *J* = 8.6 Hz, 1H, arom. H), 7.30–7.42 (m, 6H, arom. H), 10.45 (s, 1H, OH). ^13^C-NMR (DMSO-*d*_6_) *δ* (ppm): 23.2, 24.1, 25.1 (piperidinyl C-3/4/5), 44.1, 46.7 (piperidinyl C-2/6), 85.1 (quinazolinyl C-4), 116.0, 122.3, 124.86, 124.94, 127.4, 128.2, 129.3, 129.6, 134.0, 137.5 (arom. C), 150.4 (quinazolinyl C-2), 165.5 (carboxamide CO). Anal. calcd for C_20_H_20_ClN_3_O_3_ (385.85): C, 62.26; H, 5.22; N, 10.89. Found: C, 62.44; H, 5.03; N, 10.73.

#### 3-Ethyl-4-hydroxy-4-(piperidine-1-carbonyl)-3,4-dihydroquinazolin-2(1*H*)-one (13f)

It was obtained from the reaction of 10c and 11c (reaction time 24 h) as colorless microcrystals from benzene with mp 165–167 °C and yield 73% (1.10 g). IR: *ν*_max_/cm^−1^ 3318, 3198, 3121, 1667, 1636, 1609, 1504. ^1^H-NMR (DMSO-*d*_6_) *δ* (ppm): 0.61 (br s, 1H, piperidinyl H), 1.10 (t, *J* = 7.0 Hz, 4H, *CH*_3_CH_2_ + piperidinyl H), 1.37–1.53 (m, 4H, piperidinyl H), 3.00–3.33 (m, 5H, CH_3_*CH*_2_ + 3 piperidinyl H), 3.96 (br s, 1H, piperidinyl H), 6.75 (s, 1H, NH), 6.86 (d, *J* = 8.1 Hz, 1H, arom. H), 6.90–6.96 (m, 2H, arom. H), 7.25 (t, *J* = 7.6 Hz, 1H, arom. H), 9.96 (s, 1H, OH). ^13^C-NMR (DMSO-*d*_6_) *δ* (ppm): 14.3 (*C*H_3_CH_2_), 23.2, 24.1, 25.1 (piperidinyl C-3/4/5), 37.8 (CH_3_*C*H_2_), 44.5, 46.5 (piperidinyl C-2/6), 84.6 (quinazolinyl C-4), 113.6, 119.6, 121.3, 125.7, 129.5, 135.0 (arom. C), 150.3 (quinazolinyl C-2), 167.5 (carboxamide CO). Anal. calcd for C_16_H_21_N_3_O_3_ (303.36): C, 63.35; H, 6.98; N, 13.85. Found: C, 63.22; H, 6.87; N, 13.79.

#### 4-Hydroxy-4-(morpholine-4-carbonyl)-3-phenyl-3,4-dihydroquinazolin-2(1*H*)-one (13g)

It was obtained from the reaction of 10a and 11d (reaction time 20 h) as colorless microcrystals from ethanol with mp 173–175 °C and yield 71% (1.25 g). IR: *ν*_max_/cm^−1^ 3618, 3526, 3345, 3198, 1682, 1639, 1609. ^1^H-NMR (DMSO-*d*_6_) *δ* (ppm): 2.83 (t, *J* = 8.9 Hz, 1H, morpholinyl H), 2.96 (t, *J* = 9.5 Hz, 1H, morpholinyl H), 3.20–3.45 (m, 5H, morpholinyl H), 3.65–3.72 (m, 1H, morpholinyl H), 6.79 (s, 1H, NH), 6.96–7.03 (m, 3H, arom. H), 7.29–7.35 (m, 6H, arom. H), 10.28 (s, 1H, OH). ^13^C-NMR (DMSO-*d*_6_) *δ* (ppm): 43.4, 46.7 (morpholinyl NCH_2_), 64.6, 65.7 (morpholinyl OCH_2_), 85.8 (quinazolinyl C-4), 114.2, 118.7, 120.7, 121.6, 125.3, 127.2, 128.1, 128.7, 129.2, 129.6, 135.0, 137.9 (arom. C), 150.8 (quinazolinyl C-2), 166.5 (carboxamide CO). Anal. calcd for C_19_H_19_N_3_O_4_ (353.38): C, 64.58; H, 5.42; N, 11.89. Found: C, 64.64; H, 5.56; N, 11.76.

#### 6-Chloro-4-hydroxy-4-(morpholine-4-carbonyl)-3-phenyl-3,4-dihydroquinazolin-2(1*H*)-one (13h)

It was obtained from the reaction of 10b and 11d (reaction time 6 h) as colorless microcrystals from *n*-butanol with mp 189–191 °C and yield 75% (1.45 g). IR: *ν*_max_/cm^−1^ 3352, 3213, 1686, 1643, 1609. ^1^H-NMR (DMSO-*d*_6_) *δ* (ppm): 2.82–2.86 (m, 1H, morpholinyl H), 2.99 (t, *J* = 9.1 Hz, 1H, morpholinyl H), 3.23–3.43 (m, 5H, morpholinyl H), 3.64 (br d, *J* = 10.1 Hz, 1H, morpholinyl H), 6.95–7.02 (m, 3H, NH + 2 arom. H), 7.31–7.41 (m, 6H, arom. H), 10.42 (s, 1H, OH). ^13^C-NMR (DMSO-*d*_6_) *δ* (ppm): 43.4, 46.7 (morpholinyl NCH_2_), 64.8, 65.8 (morpholinyl OCH_2_), 85.8 (quinazolinyl C-4), 116.1, 124.6, 125.0, 127.3, 128.2, 129.2, 129.6, 134.1, 137.7 (arom. C), 150.5 (quinazolinyl C-2), 166.0 (carboxamide CO). Anal. calcd for C_19_H_18_ClN_3_O_4_ (387.82): C, 58.84; H, 4.68; N, 10.84. Found: C, 58.96; H, 4.78; N, 11.04.

#### 3-Ethyl-4-hydroxy-4-(morpholine-4-carbonyl)-3,4-dihydroquinazolin-2(1*H*)-one (13i)

It was obtained from the reaction of 10c and 11d (reaction time 10 h) as colorless microcrystals from benzene with mp 161–163 °C and yield 77% (1.17 g). IR: *ν*_max_/cm^−1^ 3310, 3267, 3210, 1748, 1655, 1609. ^1^H-NMR (CDCl_3_) *δ* (ppm): 1.33 (t, *J* = 7.0 Hz, 3H, *CH*_3_CH_2_), 2.94 (br t, *J* = 9.8 Hz, 1H, morpholinyl H), 3.11 (br d, *J* = 13.7 Hz, 1H, morpholinyl H), 3.23–3.27 (m, 1H, morpholinyl H), 3.34–3.55 (m, 4H, CH_3_*CH*_2_ + 2 morpholinyl H), 3.61 (t, *J* = 9.8 Hz, 1H, morpholinyl H), 3.78 (br d, *J* = 11.9 Hz, 1H, morpholinyl H), 4.01 (br d, *J* = 13.3 Hz, 1H, morpholinyl H), 6.65 (s, 1H, NH), 6.87 (d, *J* = 8.1 Hz, 1H, arom. H), 7.03 (t, *J* = 7.4 Hz, 1H, arom. H), 7.06 (t, *J* = 6.5 Hz, 1H, arom. H), 7.31 (d, *J* = 8.3 Hz, 1H, arom. H), 9.57 (s, 1H, OH). ^13^C-NMR (CDCl_3_) *δ* (ppm): 14.7 (*C*H_3_CH_2_), 38.8 (CH_3_*C*H_2_), 44.6, 46.9 (morpholinyl NCH_2_), 65.3, 66.4 (morpholinyl OCH_2_), 85.0 (quinazolinyl C-4), 114.4, 119.1, 122.6, 125.9, 130.3, 134.5 (arom. C), 152.0 (quinazolinyl C-2), 169.1 (carboxamide CO). Anal. calcd for C_15_H_19_N_3_O_4_ (305.33): C, 59.01; H, 6.27; N, 13.76. Found: C, 59.08; H, 6.36; N, 13.89.

### Single crystal X-ray studies

Experimental procedure for the single X-ray studies of compounds 10c and 13d is mentioned in the ESI.†

### Vasodilation studies

Experimental procedure utilized for vasodilation studies is mentioned in the ESI.†

### Molecular modeling studies

Experimental procedures for the molecular modeling studies are mentioned in the ESI.†

## Conflicts of interest

The authors have declared no conflict of interest.

## Supplementary Material

RA-009-C9RA04321G-s001

RA-009-C9RA04321G-s002
