# Supplementary material for: Facile synthetic approach towards vasorelaxant active 4-hydroxyquinazoline-4-carboxamides
Source: RSC Adv. 2019 Sep 10;9(49):28534–40. doi: 10.1039/c9ra04321g (PMC9071013; doi:10.1039/c9ra04321g)
Supplement: RA-009-C9RA04321G-s001 [file RA-009-C9RA04321G-s001.pdf]

Electronic Supplementary Material (ESI) for RSC Advances.
This journal is © The Royal Society of Chemistry 2019

Facile synthetic approach towards vasorelaxant active 4-hydroxyquinazoline-4-carboxamides

Marian N. Aziz,[a] Siva S. Panda,[b] ElSayed M. Shalaby,[c] Nehmedo G. Fawzy[a] and Adel S. Girgis*,[a]

[a] *Department of Pesticide Chemistry, National Research Centre, Dokki, Giza 12622, Egypt*

[b] *Department of Chemistry & Physics, Augusta University, Augusta, GA 30912, USA*

[c] *X-Ray Crystallography Lab., Physics Division, National Research Centre, Dokki, Giza 12622, Egypt*

* Author for correspondence: girgisas10@yahoo.com

Supplementary material

Table titles

Table S1. Crystal data and structure refinement parameters of compounds **10c** and **13d**.

Table S2. Selected intramolecular bond lengths (Å) of compounds **10c** and **13d**.

Table S3. Selected intramolecular bond angles (°) of compounds **10c** and **13d**.

Table S4. Hydrogen-bond geometry (Å, °) for compounds **10c** and **13d**.

Table S5. Descriptors of the BMLR-QSAR model for the synthesized vasorelaxant quinazolines (**13a-i**).

Table S6. Observed/estimated vasorelaxant properties of the synthesized quinazolines (**13a-i**) according to 2D-QSAR model.

Table S7. Molecular descriptor values of the BMLR-QSAR model for the synthesized vasorelaxant quinazolines (**13a-i**).

Table S8. Best fit values and estimated vasodilation activity values for the tested quinazolines (**13a-i**) according to the 3D-pharmacophore modeling.

Figure captions

Fig. S1. IR spectrum of compound **10c** (KBr pellet).

Fig. S2. ^{1}H-NMR spectrum of compound **10c** in DMSO-d_6.

Fig. S3. ^{13}C-NMR spectrum of compound **10c** in DMSO-d_6.

Fig. S4. IR spectrum of compound **13a** (KBr pellet).

Fig. S5. ^{1}H-NMR spectrum of compound **13a** in DMSO-d_6.

Fig. S6. ^{13}C-NMR spectrum of compound **13a** in DMSO-d_6.

Fig. S7. IR spectrum of compound **13b** (KBr pellet).

Fig. S8. ^{1}H-NMR spectrum of compound **13b** in DMSO-d_6.

Fig. S9. ^{13}C-NMR spectrum of compound **13b** in DMSO-d_6.

Fig. S10. IR spectrum of compound **13c** (KBr pellet).

Fig. S11. ^{1}H-NMR spectrum of compound **13c** in DMSO-d_6.

Fig. S12. ^{13}C-NMR spectrum of compound **13c** in DMSO-d_6.

Fig. S13. IR spectrum of compound **13d** (KBr pellet).

Fig. S14. ^{1}H-NMR spectrum of compound **13d** in DMSO-d_6.

Fig. S15. ^{13}C-NMR spectrum of compound **13d** in DMSO-d_6.

Fig. S16. IR spectrum of compound **13e** (KBr pellet).

Fig. S17. ^{1}H-NMR spectrum of compound **13e** in DMSO-d_6.

Fig. S18. ^{13}C-NMR spectrum of compound **13e** in DMSO-d_6.

Fig. S19. IR spectrum of compound **13f** (KBr pellet).

Fig. S20. ^{1}H-NMR spectrum of compound **13f** in DMSO-d_6.

Fig. S21. ^{13}C-NMR spectrum of compound **13f** in DMSO-d_6.

Fig. S22. IR spectrum of compound **13g** (KBr pellet).

Fig. S23. ^{1}H-NMR spectrum of compound **13g** in DMSO-d_6.

Fig. S24. ^{13}C-NMR spectrum of compound **13g** in DMSO-d_6.

Fig. S25. IR spectrum of compound **13h** (KBr pellet).

Fig. S26. ^{1}H-NMR spectrum of compound **13h** in DMSO-d_6.

Fig. S27. ^{13}C-NMR spectrum of compound **13h** in DMSO-d_6.

Fig. S28. IR spectrum of compound **13i** (KBr pellet).

Fig. S29. ^{1}H-NMR spectrum of compound **13i** in $CDCl_3$.

Fig. S30. ^{13}C-NMR spectrum of compound **13i** in $CDCl_3$.

Fig. S31. A view of the unit cell contents for **10c** showing the H-bonds as dashed lines.

Fig. S32. A view of the unit cell contents for **13d** showing the H-bonds as dashed lines.

Fig. S33. Effect of synthesized compounds (**10c**, **13a-i**) and Doxazosin on contracture induced by norepinephrine hydrochloride (NE.HCl) in rat thoracic aortic rings.

Fig. S34. Potency (IC_{50}, μM) of the tested compounds on contracture induced by norepinephrine hydrochloride in rat thoracic aortic rings compared with Doxazosin used as a reference standard.

Fig. S35. (A) Constraint distances "H-1 – H-2 = 5.886, H-1 – HBD = 4.853, H-2 – HBD = 5.669 Å", (B) Constraint angle "H-2 – H-2 – HBD = 62.86 °" of the generated 3D-pharmacophore for the tested quinazoline-4-carboxamides **13a–i** which contains two hydrophobics (H-1 and H-2; light blue) and one hydrogen bonding donor (HBD; purple).

Fig. S36. 3D-pharmacophore model mapped on the tested quinazoline-4-carboxamides **13a–i**.

Single crystal X-ray studies

Data collections for compounds **10c** and **13d** were performed at the X-ray diffraction beamline (XRD1) of the Elettra Synchrotron, Trieste (Italy).[1] Crystals were dipped in NHV oil (Jena Bioscience GmbH) and mounted on the goniometer head with a nylon loop. Complete datasets were collected at room temperature through the rotating crystal method. Data were acquired using a monochromatic wavelength of 0.700 Å on a Pilatus 2M hybrid-pixel area detector. The diffraction data were indexed and integrated using XDS.[2]

The structures were solved by SUPERFLIP[3] implemented in the CRYSTALS program suit.[4] Fourier analysis and refinement were performed by the full-matrix least-squares methods based on F^2 implemented in CRYSTALS. The hydrogen atoms were positioned geometrically in their idealized positions.[5]The general-purpose program, *PLATON*[6] was used for the structure analysis and presentation of the results. *ORTEP-3* for Windows[7] and *MERCURY*[8] programs were used for molecular graphics representations. Essential crystal and refinement data (Table S1) are reported below. Geometrical parameters are summarized in ESI Tables S2, S3. Crystallographic data for the structures in this paper have been deposited with the Cambridge Crystallographic Data Center as supplementary publication numbers CCDC 1913146 and CCDC 1913147. Copies of the data can be obtained, free of charge, on application to CCDC, 12 Union Road, Cambridge CB2 1EZ, UK [Fax: 144(0)1223 336033 or e-mail: deposit@ccdc.cam.ac.uk].

Vasodilation studies

The vasodilation activity screening was undertaken by Pharmacology Department, National Research Centre, Egypt, according to the standard *in vitro* bioassay technique,[9] by testing the effects of the synthesized agents **10c** and **13a-i** and compared with Doxazosin (α_1-AR antagonist) on isolated thoracic aortic rings of male Wistar rats (200–250 g) pre-contracted with norepinephrine hydrochloride. After light ether anesthesia, the rats were sacrificed by cervical dislocation. The aortae were immediately excised, freed of extraneous tissues, and prepared for isometric tension recording. Aorta was cut into (3–5 mm width) rings and each ring was placed in a vertical chamber "10 ml

jacketed automatic multi-chamber organ bath system (Model no. ML870B6/C, Panlab, Spain)" filled with Krebs solution composed of (in mM): NaCl, 118.0; KCl, 4.7; $NaHCO_3$, 25.0; $CaCl_2$, 1.8; NaH_2PO_4, 1.2; $MgSO_4$, 1.2; glucose, 11.0 and oxygenated with carbogen gas (95% O2/5% CO_2) at 37 ± 0.5 °C. Each aortic ring was mounted between two stainless steel hooks passed through its lumen. The lower hook was fixed between two plates, while the upper one was attached to a force displacement transducer (Model no. MLT0201, Panlab, Spain) connected to an amplifier (PowerLab, AD Instruments Pty. Ltd.), which was connected to a computer. The Chart for windows (v 3.4) software was used to record and elaborate data.

Preparations were stabilized under 2 g resting tension during 2 h, and then the contracture response to norepinephrine hydrochloride (10^{-6} M) was measured before and after exposure to increasing concentrations of the tested synthesized compounds. The tested compounds were dissolved in dimethylsulfoxide (DMSO) as stock solution (10 ml of 0.005 M). Control experiments were performed in the presence of DMSO alone, at the same concentrations as those used with the derivatives tested, which demonstrated that the solvent did not affect the contractile response of isolated aorta. The observed vasodilation activity screening data for the synthesized compounds **10c**, **13a-i** and doxazosin are expressed as IC_{50} (μM) concentration necessary for 50% reduction of maximal norepinephrine hydrochloride induced contracture.

2D-QSAR study

The synthesized quinazoline (**13a-i**) revealing variable vasorelaxant properties were utilized for developing the 2D-QSAR modeling by CODESSA-Pro (comprehensive descriptors for structural and statistical analysis) software. Geometry of the compounds was initially optimized by AM1 technique using hyperChem 8.0 then, uploaded to CODESSA-Pro for final geometrical structure optimization by MOPAC.[10] CODESSA-Pro calculated 670 molecular descriptors (constitutional, topological, geometrical, charge-related, semi-empirical, thermodynamic, molecular-type, atomic-type and bond-type descriptors) for the exported agents. Mathematical transformation of the experimental values (including IC_{50}, $1/IC_{50}$, $\log(IC_{50})$ and $1/\log(IC_{50})$ µM) were used searching for the best QSAR model. The best multi-linear regression (BMLR) technique

was utilized which is a stepwise search for the best *n*-parameter regression equations (where *n* stands for the number of descriptors used), based on the highest R^2 (squared correlation coefficient), R^2cvOO (squared cross-validation "leave one-out, LOO" coefficient), R^2cvMO (squared cross-validation "leave many-out up to 20% of the training set, LMO" coefficient), *F* (Fisher statistical significance criteria) values, and s^2 (standard deviation). The QSAR up to 2-descriptor model describing the biological activity of the active agents were generated (obeying the thumb rule of about 4.5:1 which is the ratio between the data points and the number of QSAR descriptor).

ZX shadow/ZX rectangle is a geometrical descriptor with *t*-criterion value (level of significance) = 9.331. Due to its high coefficient value (3.03178), compounds with high descriptor value reveal low biological properties and vice versa, as shown for compounds **13h**, **13i** which possess descriptor values = 0.61476, 0.71826 corresponding to predicted values = 164, 441 μM, respectively (ESI Tables S5–S7). Relative shadow areas of a molecule can be calculated by equ. (1).[11]

$$S_k^r = \frac{\oint_{(C)} (v d\rho - \rho dv)}{S^{(k)}} \quad \ldots\ldots\ldots\ldots\ldots (1)$$

Where, *C* is the contour of projection for the molecule on the plane (defined by principal axes $k = XY, XZ$ or YZ plane), v - x or y, ρ - y or z and $S^{(k)} = X{\bullet}Y; X{\bullet}Z$ or $Y{\bullet}Z$.

Topographic electronic index (all pairs) is a charge-related descriptor with negative sign in the QSAR model (coefficient value = -0.356238). This is why the compounds with high descriptor value possess high predicted efficacy and vice versa as exhibited for pairs **13h**/**13i** (descriptor values = 5.55987, 5.23453, respectively). Topological electronic indices can be calculated by equ. (2, 3).[11]

$$T_1^E = \sum_{(i<j)}^{N_{SA}} \frac{|q_i - q_j|}{r_{ij}^2} \quad \ldots\ldots\ldots\ldots\ldots (2)$$

$$T_2^E = \sum_{(i<j)}^{N_b} \frac{|q_i - q_j|}{r_{ij}^2} \quad \ldots\ldots\ldots\ldots\ldots (3)$$

Where, q_i is the partial charge on the ith atom. r_{ij} is the distance between the ith and j^{th} atoms. N_{SA} is the number of non-hydrogen atom in the molecule. N_b is the number of bonds between non-hydrogen atom in the molecule.

3D-Pharmacophore modeling study

3D-Pharmacophoric study was undertaken by the standard technique utilizing Discovery Studio 2.5 software for the vasorelaxant active quinazolines **13a-i**.[12]

References

1. A. Lausi, M. Polentarutti, S. Onesti, J. R. Plaisier, E. Busetto, G. Bais, L. Barba, A. Cassetta, G. Campi, D. Lamba, A. Pifferi, S. C. Mande, D. D. Sarma, S. M. Sharma and G. Paolucci, Status of the crystallography beamlines at Elettra, *Eur. Phy. J. Plus* 2015, **130: 43**, 1–8.
2. W. Kabsch, XDS, *Acta Cryst. Sect. D* 2010, **66**, 125–132.
3. L. Palatinus and G. Chapuis, *SUPERFLIP* – a computer program for the solution of crystal structures by charge flipping in arbitrary dimensions, *J. Appl. Cryst.* 2007, **40**, 786–790.
4. P. W. Betteridge, J. R. Carruthers, R. I. Cooper, K. Prout and D. J. Watkin, *CRYSTALS* version 12: software for guided crystal structure analysis, *J. Appl. Cryst.* 2003, **36**, 1487.
5. R. I. Cooper, A. L. Thompson and D. J. Watkin, *CRYSTALS* enhancements: dealing with hydrogen atoms in refinement, *J. Appl. Cryst.* 2010, **43**, 1100–1107.
6. A. L. Spek, Structure validation in chemical crystallography, *Acta Cryst. Sect. D* 2009, **65**, 148–155.
7. L. J. Farrugia, *WinGX* and *ORTEP for Windows*: an update, *J. Appl. Cryst.* 2012, **45**, 849–854.
8. C. F. Macrae, I. J. Bruno, J. A. Chisholm, P. R. Edgington, P. McCabe, E. Pidcock, L. Rodriguez-Monge, R. Taylor, J. van de Streek and P. A. Wood, *Mercury CSD 2.0* – new features for the visualization and investigation of crystal structures, *J. Appl. Cryst.* 2008, **41**, 466–470.
9. E. M. Shalaby, A. S. Girgis, H. Farag, A. F. Mabied and A. N. Fitch, Synthesis, X-ray powder diffraction and DFT calculations of vasorelaxant active 3-(arylmethylidene)pyrrolidine-2,5-diones, *RSC Adv.* 2016, **6**, 112950–112959.
10. A. M. Srour, S. S. Panda, A. M. M. Salman, M. A. El-Manawaty, R. F. George, E.

M. Shalaby, A. N. Fitch, N. G. Fawzy and A. S. Girgis, Synthesis and molecular modeling studies of bronchodilatory active indole-pyridine conjugates, *Future Med. Chem.* 2018, **10**, 1787–1804.

11. A. R. Katritzky, R. Petrukhin, I. Petrukhina, A. Lomaka, D. B. Tatham and M. Karelson, CODESSA-Pro software manual 2005, pp. 44, 48.
12. S. S. Panda, O. S. Detistov, A. S. Girgis, P. P. Mohapatra, A. Samir and A. R. Katritzky, Synthesis and molecular modeling of antimicrobial active fluoroquinolone-pyrazine conjugates with amino acid linkers, *Bioorg. Med. Chem. Lett.* 2016, **26**, 2198–2205.

Table S1. Crystal data and structure refinement parameters of compounds **10c** and **13d**.

Parameters	Compd. **10c**	Compd. **13d**
Crystal data		
Chemical formula	$C_{11}H_{10}N_2O_3$	$C_{20}H_{21}N_3O_3$
M_r	218.21	351.40
Crystal system, space group	Monoclinic, *C*2/*c*	Monoclinic, $P2_1/n$
Temperature (K)	293	293
a, *b*, *c* (Å)	10.926 (2), 21.310 (4), 8.9155 (18)	17.500 (4), 13.189 (3), 17.709 (4)
β (°)	94.02 (3)	117.61 (3)
V (Å^3)	2070.7 (4)	3621.9 (17)
Z	8	8
Radiation type	Synchrotron	Synchrotron
μ (mm^{-1})	0.10	0.09
Crystal size (mm)	0.07 × 0.07 × 0.06	0.10 × 0.09 × 0.06
Data collection		
Absorption correction	Multi-scan *DENZO*/*SCALEPACK* (Otwinowski & Minor, 1997)	Multi-scan *DENZO*/*SCALEPACK* (Otwinowski & Minor, 1997)
T_{min}, T_{max}	0.99, 0.99	0.99, 0.99
No. of measured, independent and observed [I > 2.0σ(I)] reflections	26527, 3404, 3317	67134, 11051, 10075
R_{int}	0.024	0.021
(sin θ/λ)$_{max}$ (Å^{-1})	0.735	0.714
Refinement		

$R[F^2 > 2\sigma(F^2)]$, $wR(F^2)$, S	0.066, 0.185, 1.20	0.045, 0.110, 1.18
No. of reflections	3317	10075
No. of parameters	149	470
$\Delta\rho_{max}$, $\Delta\rho_{min}$ (e Å^{-3})	0.39, −0.32	0.31, −0.29
CCDC deposition number	1913146	1913147

Table S2. Selected intramolecular bond lengths (Å) of compounds **10c** and **13d**.

Geometric parameters	Compd. **10c**	Geometric parameters	Compd. **13d**	
			Moiety 1	Moiety 2
O1—C2	1.216	N1—C1	1.3605	1.361
C2—N3	1.4325	N1—C2	1.3914	1.3914
C2—N14	1.3398	N2—C1	1.3831	1.3803
N3—C4	1.4347	N2—C8	1.4734	1.476
N3—C11	1.391	N2—C15	1.4422	1.4407
C4—C5	1.3904	N3—C9	1.34	1.3399
C4—C9	1.4023	N3—C10	1.4749	1.4708
C5—C6	1.3911	N3—C14	1.4683	1.4627
C6—C7	1.388	C1—O1	1.2315	1.2341
C7—C8	1.394	C2—C3	1.4014	1.4001
C8—C9	1.3929	C2—C7	1.3918	1.3901
C9—C10	1.4501	C3—C4	1.3827	1.384
C10—C11	1.5439	C4—C5	1.3889	1.3846
C10—O13	1.2134	C5—C6	1.3891	1.3883
C11—O12	1.2114	C6—C7	1.3914	1.3934
N14—C15	1.4582	C7—C8	1.5131	1.5111
C15—C16	1.455	C8—C9	1.5658	1.5642
		C8—O2	1.4108	1.4074
		C9—O3	1.2346	1.2325
		C10—C11	1.5109	1.5113
		C11—C12	1.5207	1.5148
		C12—C13	1.5205	1.5208
		C13—C14	1.525	1.5237
		C15—C16	1.3878	1.3911
		C15—C20	1.3886	1.3871
		C16—C17	1.392	1.3884
		C17—C18	1.3799	1.3751

		C18—C19	1.3725	1.3779
		C19—C20	1.396	1.394

Table S3. Selected intramolecular bond angles (°) of compounds **10c** and **13d**.

Geometric parameters	Compd. **10c**	Geometric parameters	Compd. **13d**	
			Moiety 1	Moiety 2
O1—C2—N3	119.83	C1—N1—C2	123.7	124.07
O1—C2—N14	125.33	C1—N2—C8	118.9	119.58
N3—C2—N14	114.82	C1—N2—C15	117.49	117.98
C2—N3—C4	124.3	C8—N2—C15	119.7	119.35
C2—N3—C11	125.84	C9—N3—C10	117.59	118.75
C4—N3—C11	109.53	C9—N3—C14	127.06	128.03
N3—C4—C5	128.72	C10—N3—C14	114.45	113.22
N3—C4—C9	110.16	N2—C1—N1	116.22	116.19
C5—C4—C9	121.09	N2—C1—O1	122.31	122.26
C4—C5—C6	116.82	N1—C1—O1	121.44	121.51
C5—C6—C7	122.64	N1—C2—C3	120.69	120.7
C6—C7—C8	120.48	N1—C2—C7	118.81	118.92
C7—C8—C9	117.57	C3—C2—C7	120.5	120.38
C4—C9—C8	121.38	C2—C3—C4	119.37	119.4
C4—C9—C10	108.33	C3—C4—C5	120.52	120.67
C8—C9—C10	130.23	C4—C5—C6	119.89	119.76
C9—C10—C11	105.46	C5—C6—C7	120.43	120.49
C9—C10—O13	131.93	C2—C7—C6	119.26	119.29
C11—C10—O13	122.53	C2—C7—C8	117.17	117.53
C10—C11—N3	106.49	C6—C7—C8	123.37	122.89
C10—C11—O12	125.61	C7—C8—N2	109.32	109.75
N3—C11—O12	127.9	C7—C8—C9	111.82	112.19
C2—N14—C15	121.81	N2—C8—C9	111	109.77
N14—C15—C16	112.9	C7—C8—O2	106.77	106.63
		N2—C8—O2	109.74	109.97
		C9—C8—O2	108.07	108.47
		C8—C9—N3	121.12	120.48
		C8—C9—O3	116.26	116.91
		N3—C9—O3	122.56	122.6
		N3—C10—C11	111.21	110.49
		C10—C11—C12	110.71	110.88
		C11—C12—C13	109.84	111.17

		C12—C13—C14	111.86	111.92
		C13—C14—N3	111.4	110.61
		N2—C15—C16	119.01	118.95
		N2—C15—C20	121.26	121.15
		C16—C15—C20	119.67	119.83
		C15—C16—C17	120.02	119.98
		C16—C17—C18	120.29	120.3
		C17—C18—C19	119.74	119.81
		C18—C19—C20	120.79	120.79
		C19—C20—C15	119.48	119.25

Table S4. Hydrogen-bond geometry (Å, °) for compounds **10c** and **13d**.

Compd.	*D*—H···*A*	*D*—H	H···*A*	*D*···*A*	*D*—H···*A*
**10c**	N14—H141···O12^{i}	0.92(2)	1.99(3)	2.709(2)	134.6(13)
	C8—H81···O1^{ii}	0.95(3)	2.48(4)	3.414(2)	168.0(12)
	C5—H51···O13^{ii}	0.95(4)	2.66(3)	3.300(3)	124.9(13)
**13d**	N1—H11···O1^{iii}	0.87	1.96	2.827(2)	180
	O2—H21···O3^{iv}	0.86	2.20	2.925(3)	141
	C20—H201···O3^{iv}	0.95	2.56	3.485(3)	166
	N21—H211···O21^{v}	0.87	1.95	2.819(2)	177
	O22—H221···O23^{iv}	0.84	2.13	2.837(2)	141

Symmetry codes: (i) -x,1-y,1-z; (ii) -1/2+x,1/2-y,1/2+z; (iii) 1-x,2-y,2-z; (iv) 1-x,1-y,2-z; (v) 1-x,2-y,1-z; (vi) 1-x,1-y,1-z

Table S5. Descriptors of the BMLR-QSAR model for the synthesized vasorelaxant quinazolines (**13a-i**).

Entry	ID	Coefficient	*s*	*t*	Descriptor
1	0	2.33137	0.320	7.281	Intercept
2	D_1	3.03178	0.325	9.331	ZX Shadow/ZX Rectangle
3	D_2	-0.356238	0.040	-8.949	Topographic electronic index (all pairs)
$N = 9$, $n = 2$, $R^2 = 0.970$, R^2cvOO = 0.905, R^2cvMO = 0.937, $F = 98.592$, $s^2 = 0.001$<br>Log(IC$_{50}$, μM) = 2.33137 + (3.03178 x D_1) – (0.356238 x D_2)					

Table S6. Observed/estimated vasorelaxant properties of the synthesized quinazolines (**13a-i**) according to 2D-QSAR model.

Entry	Compd.	Observed (IC$_{50}$, μM)	Log[observed (IC$_{50}$, μM)]	Predicted (IC$_{50}$, μM)	Log[predicted (IC$_{50}$, μM)]	Error[a]
1	**13a**	302	2.48001	292	2.46575	10
2	**13b**	415	2.61805	421	2.62458	-6
3	**13c**	392	2.59329	380	2.57985	12
4	**13d**	332	2.52114	328	2.51622	4
5	**13e**	250	2.39794	268	2.42867	-18
6	**13f**	305	2.4843	303	2.481	2
7	**13g**	298	2.47422	270	2.43184	28
8	**13h**	158	2.19866	164	2.21454	-6
9	**13i**	416	2.61909	441	2.64423	-25

[a] Error is the difference between the observed and estimated IC$_{50}$ (μM) vasorelaxant values.

Table S7. Molecular descriptor values of the BMLR-QSAR model for the synthesized vasorelaxant quinazolines (**13a-i**).

Entry	Compound	Descriptors[a]	
		D_1	D_2
1	**13a**	0.65032	5.15739
2	**13b**	0.66837	4.86514
3	**13c**	0.66393	4.9529
4	**13d**	0.66933	5.17745
5	**13e**	0.65465	5.29831
6	**13f**	0.63608	4.99336
7	**13g**	0.67779	5.4864
8	**13h**	0.61476	5.55987
9	**13i**	0.71826	5.23453

[a] D_1 = ZX Shadow/ZX Rectangle, D_2 = Topographic electronic index (all pairs).

Table S8. Best fit values and estimated vasodilation activity values for the tested quinazolines (**13a-i**) according to the 3D-pharmacophore modeling.

Entry	Compd.	Observed (IC_{50}, μM)	Estimated (IC_{50}, μM)	Error[a]	Fit value
1	**13a**	302	324	-22	5.551
2	**13b**	415	445	-30	5.413
3	**13c**	392	363	29	5.502
4	**13d**	332	319	13	5.557
5	**13e**	250	237	13	5.686
6	**13f**	305	400	-95	5.459
7	**13g**	298	318	-20	5.559
8	**13h**	158	150	8	5.885
9	**13i**	416	337	79	5.534

[a] Error is the difference between the observed and estimated IC_{50} (μM) vasorelaxant values.

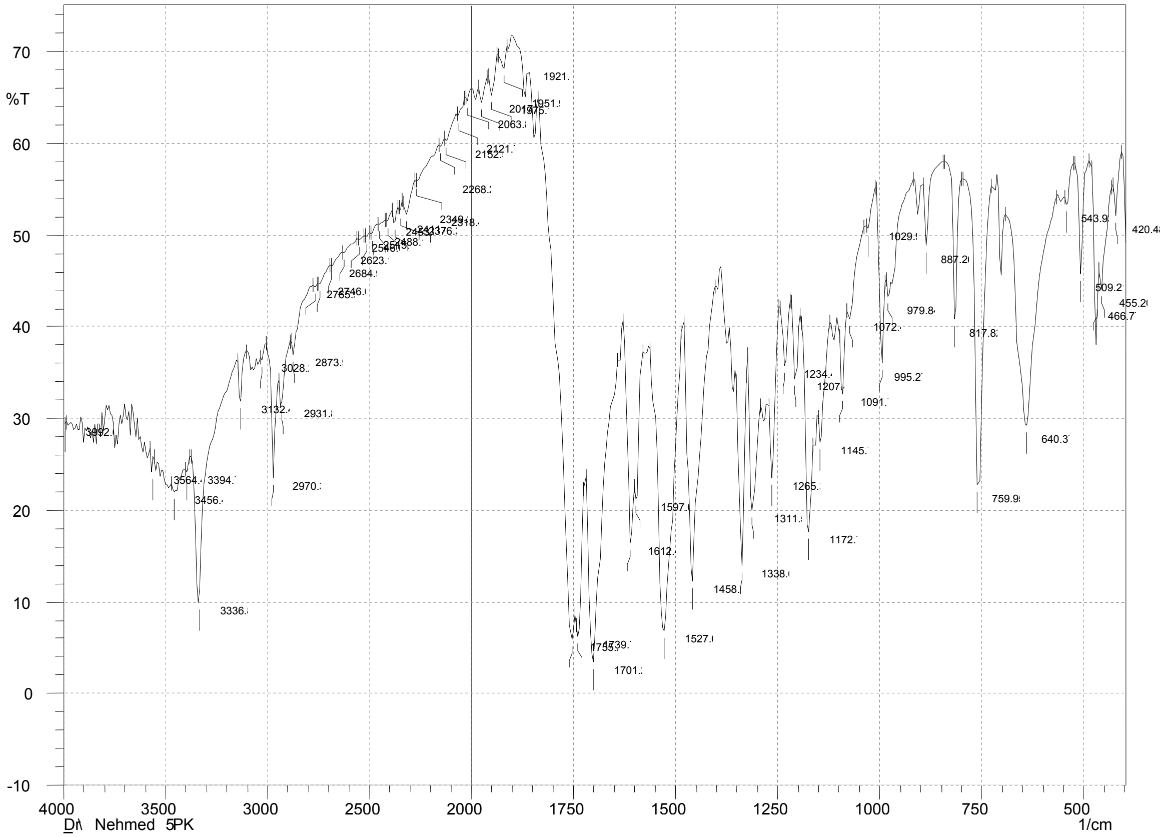

Fig. S1. IR spectrum of compound **10c** (KBr pellet).

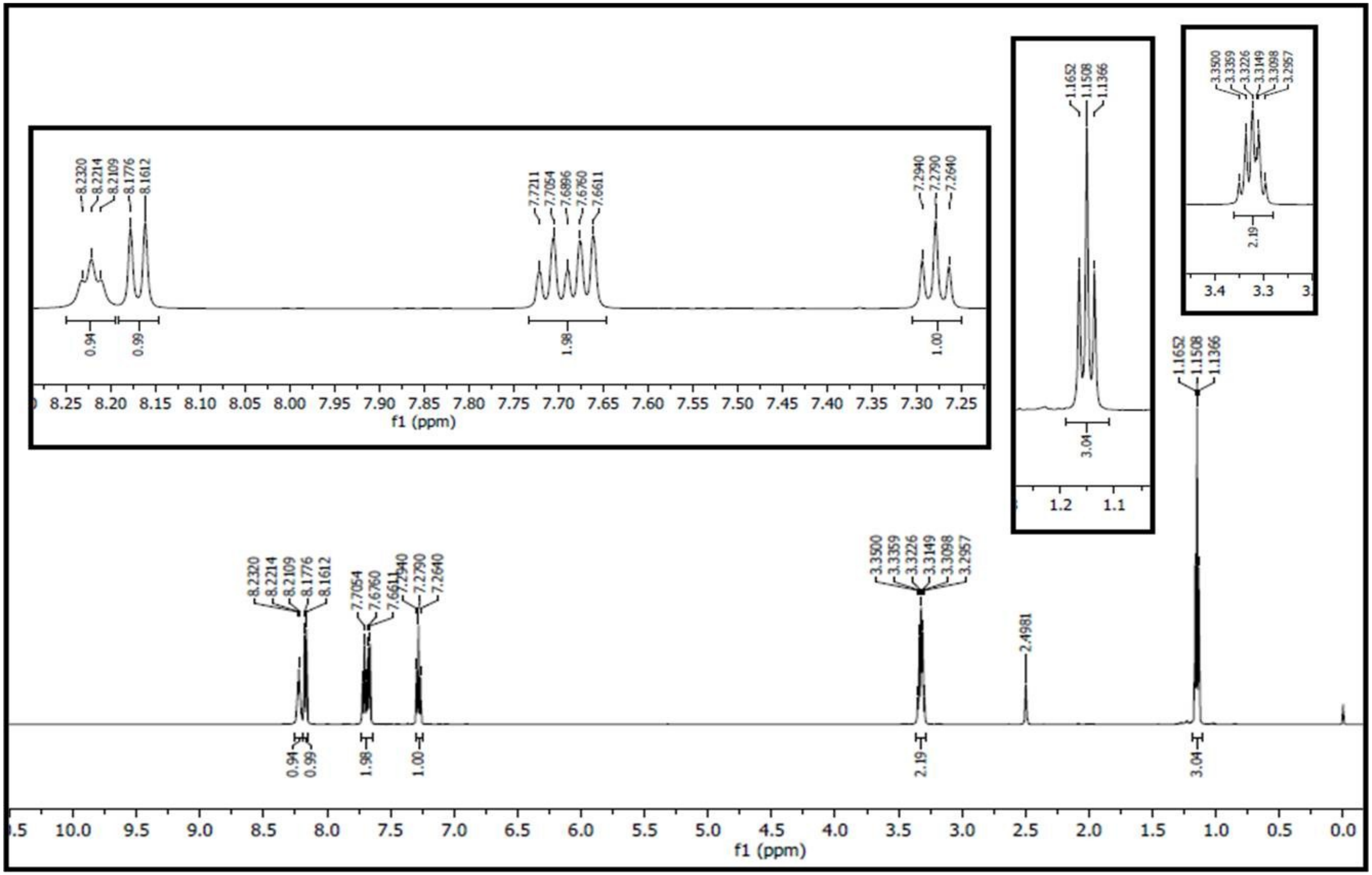

Fig. S2. ^{1}H-NMR spectrum of compound **10c** in DMSO-d_6.

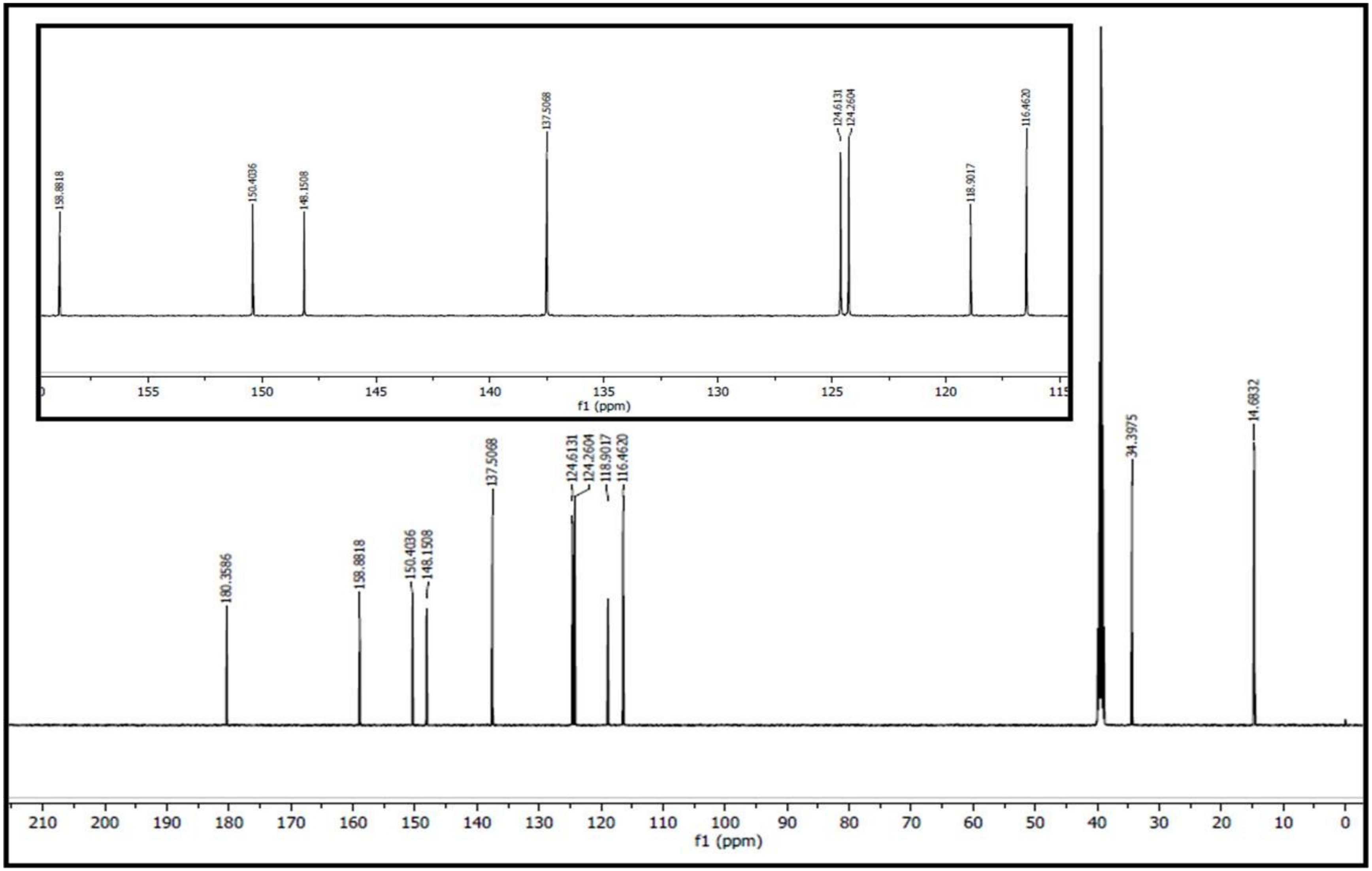

Fig. S3. ^{13}C-NMR spectrum of compound **10c** in DMSO-d_6.

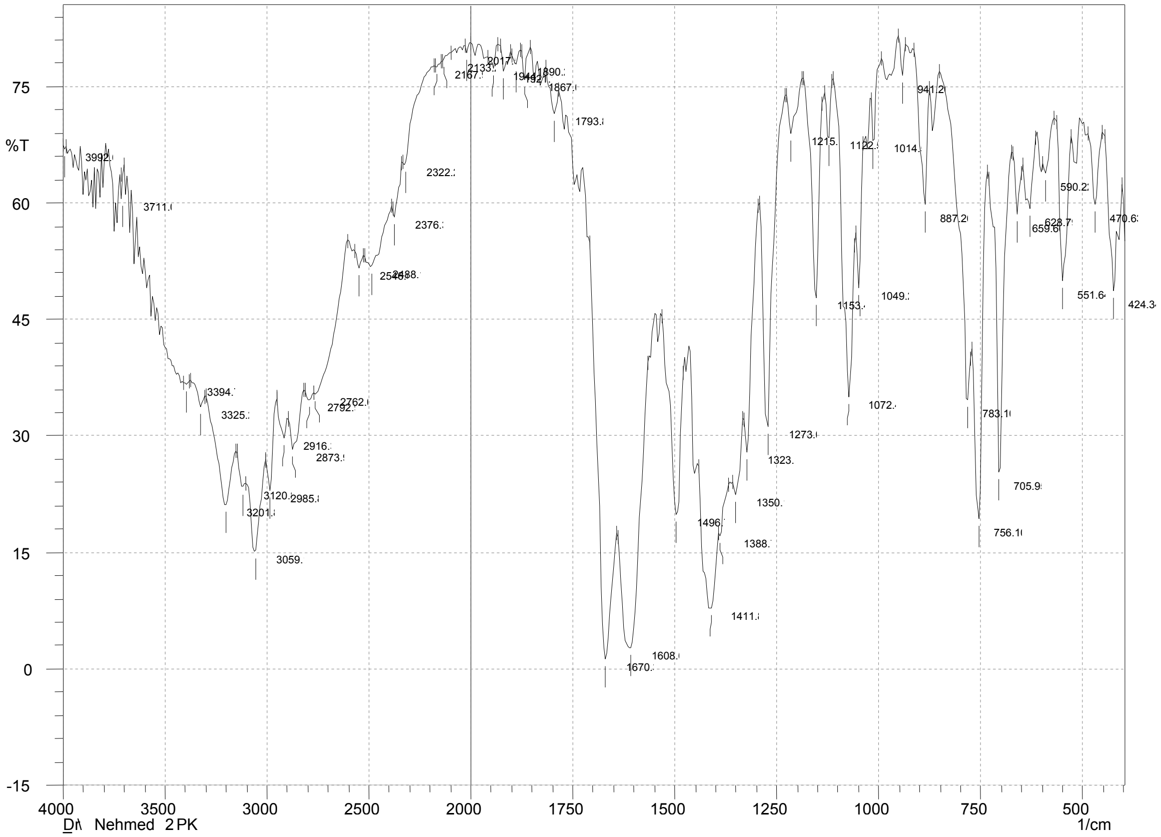

Fig. S4. IR spectrum of compound **13a** (KBr pellet).

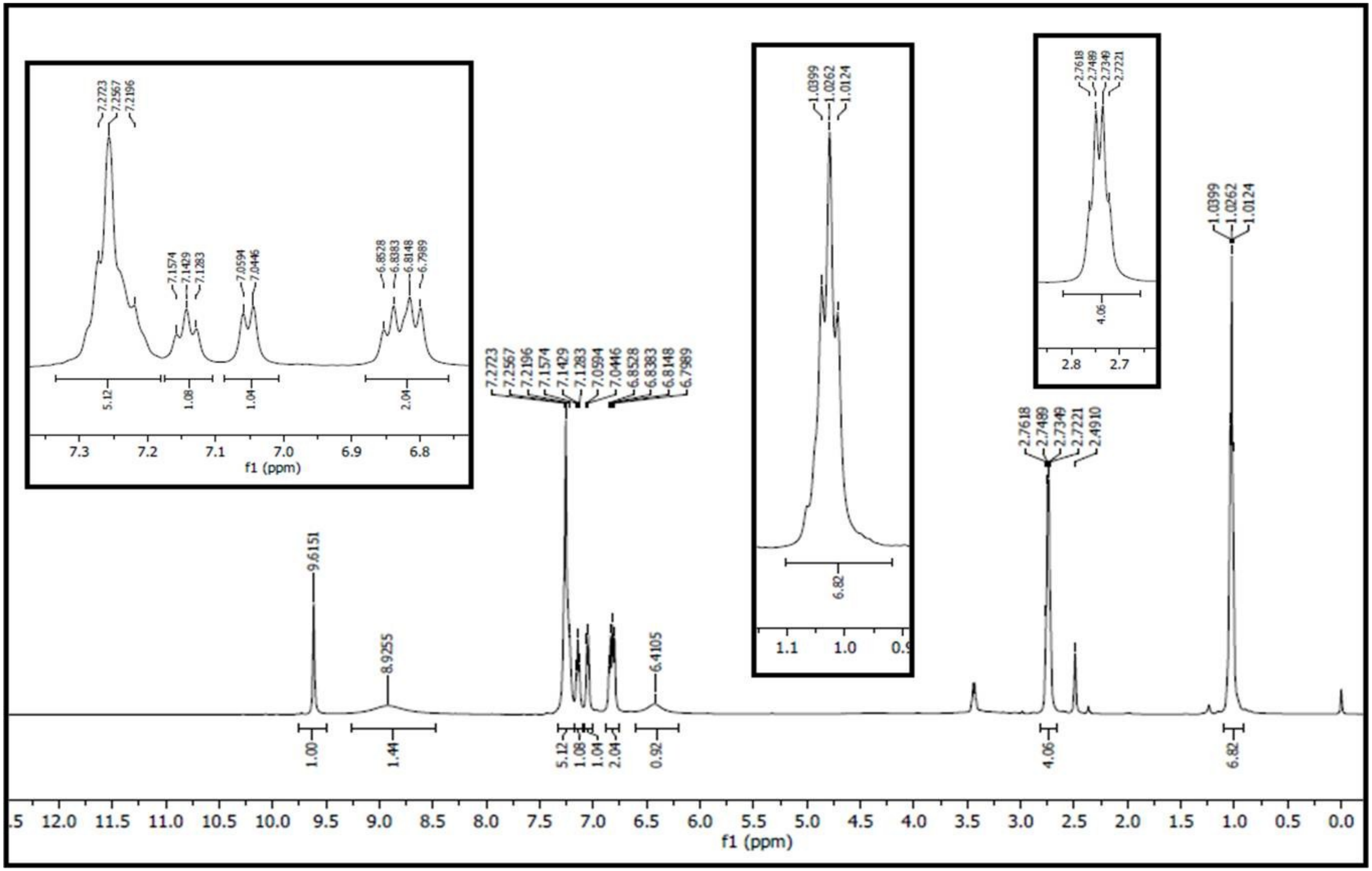

Fig. S5. ^{1}H-NMR spectrum of compound **13a** in DMSO-d_6.

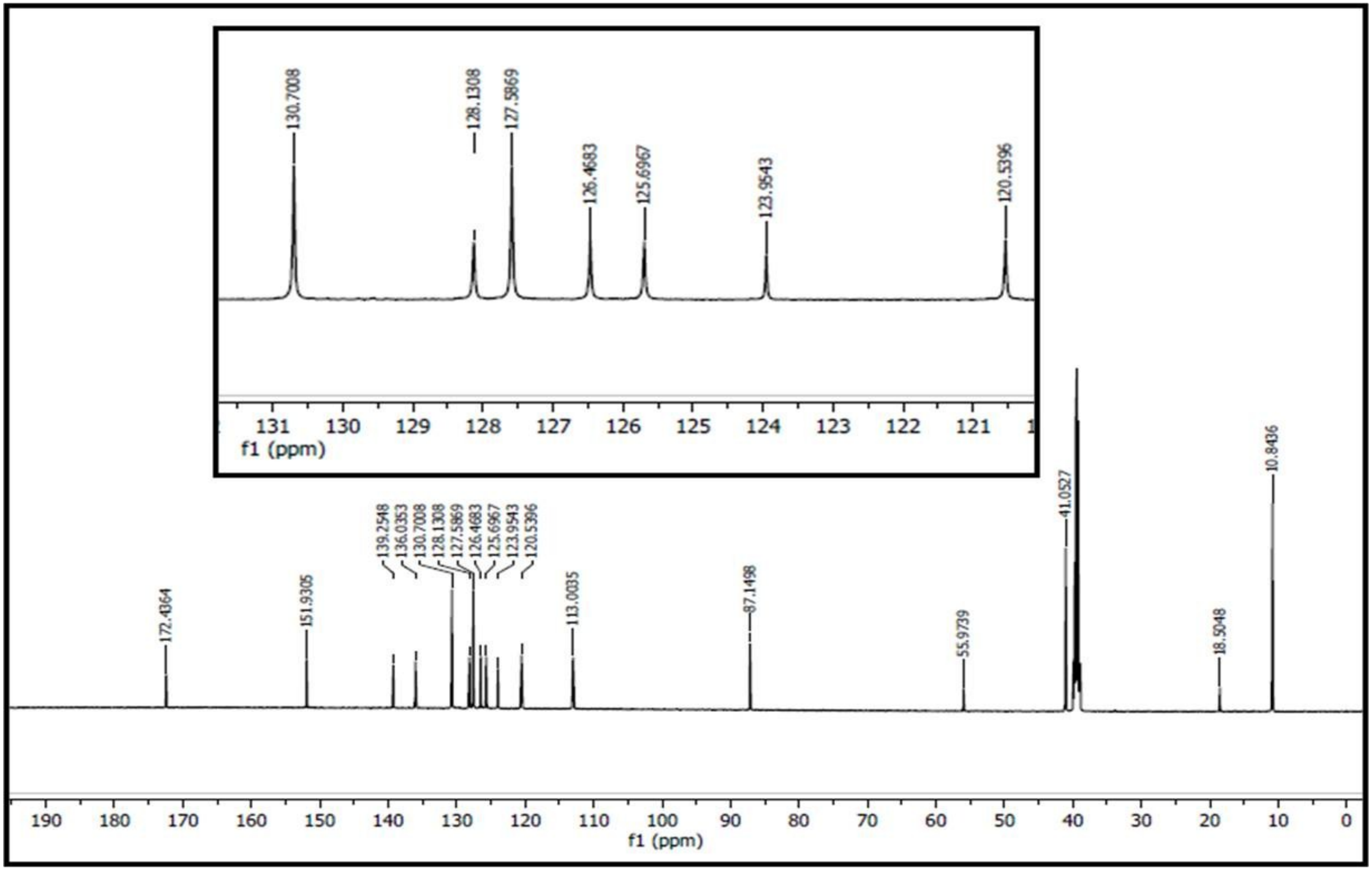

Fig. S6. ^{13}C-NMR spectrum of compound **13a** in DMSO-d_6.

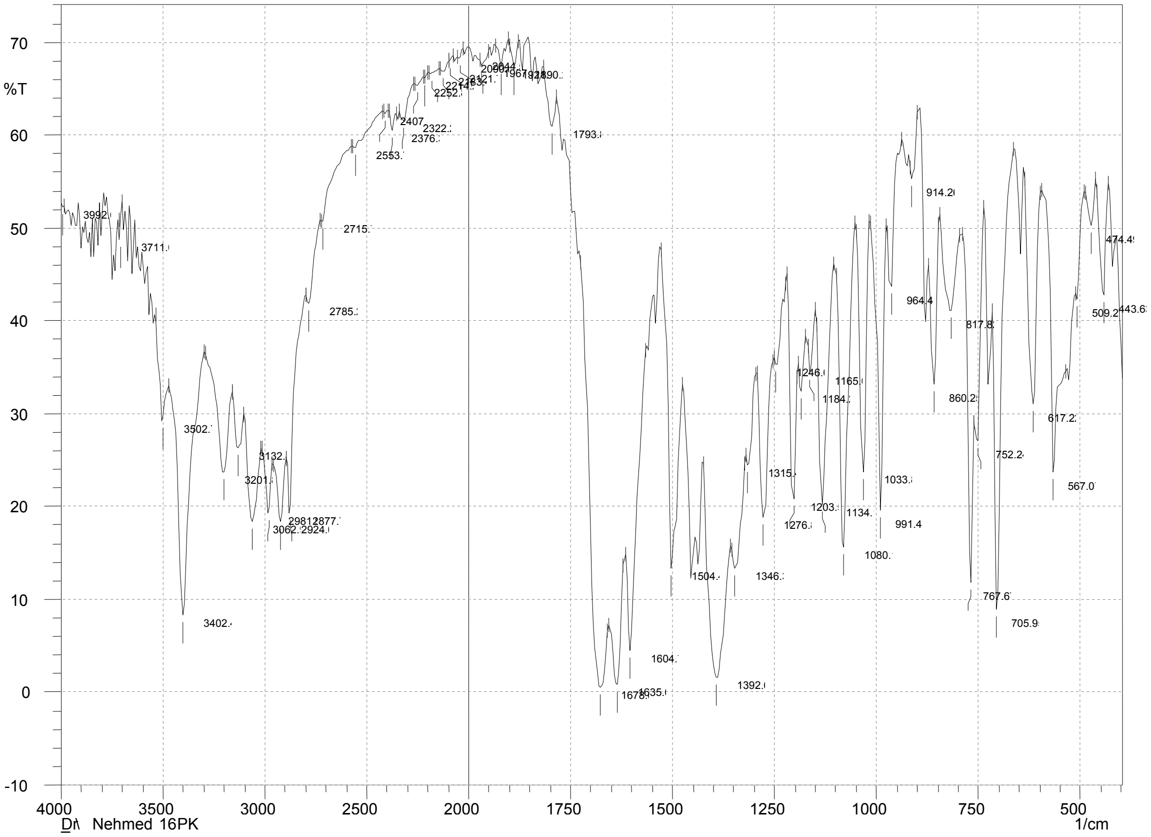

Fig. S7. IR spectrum of compound **13b** (KBr pellet).

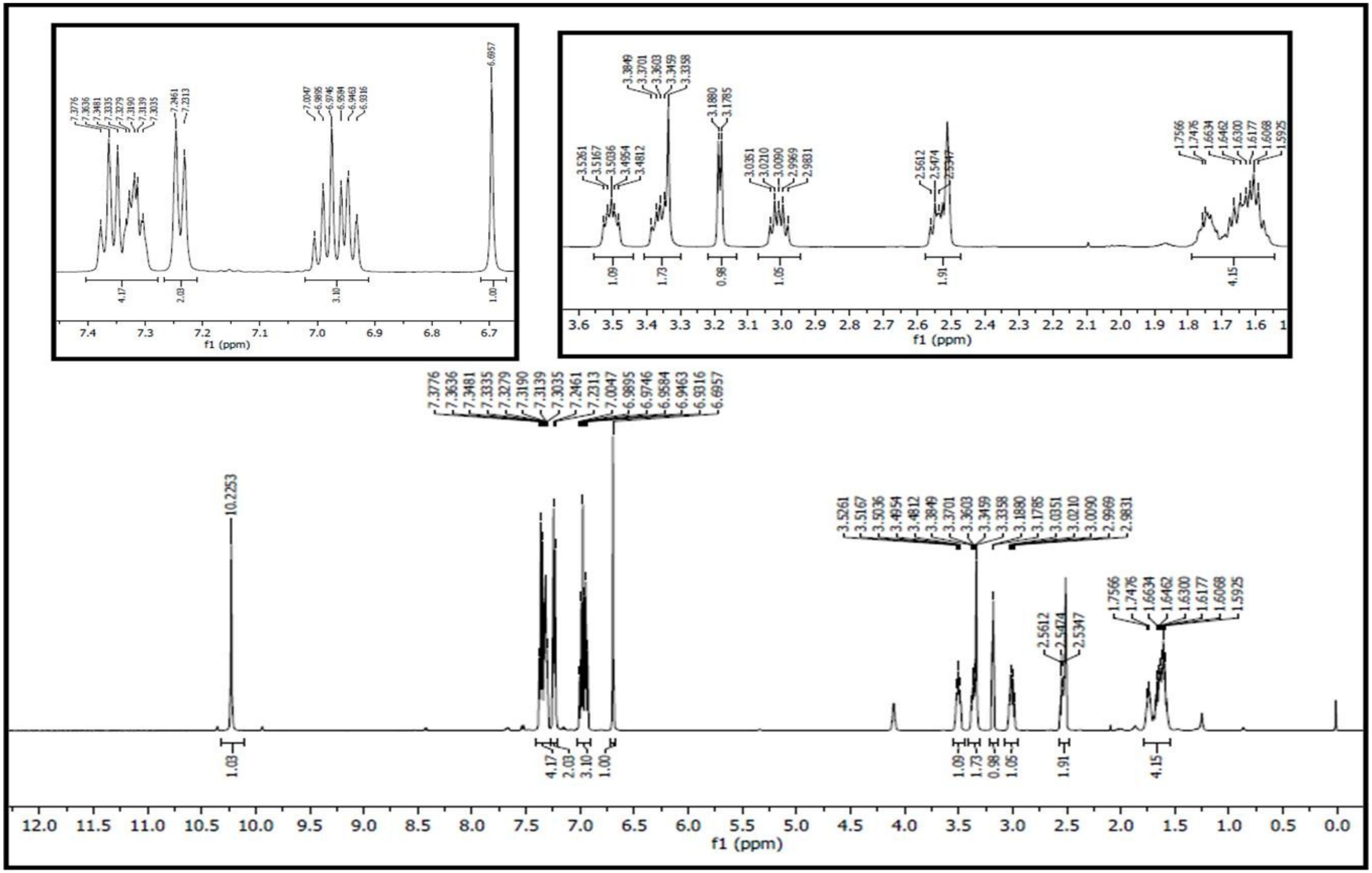

Fig. S8. ^{1}H-NMR spectrum of compound **13b** in DMSO-d_6.

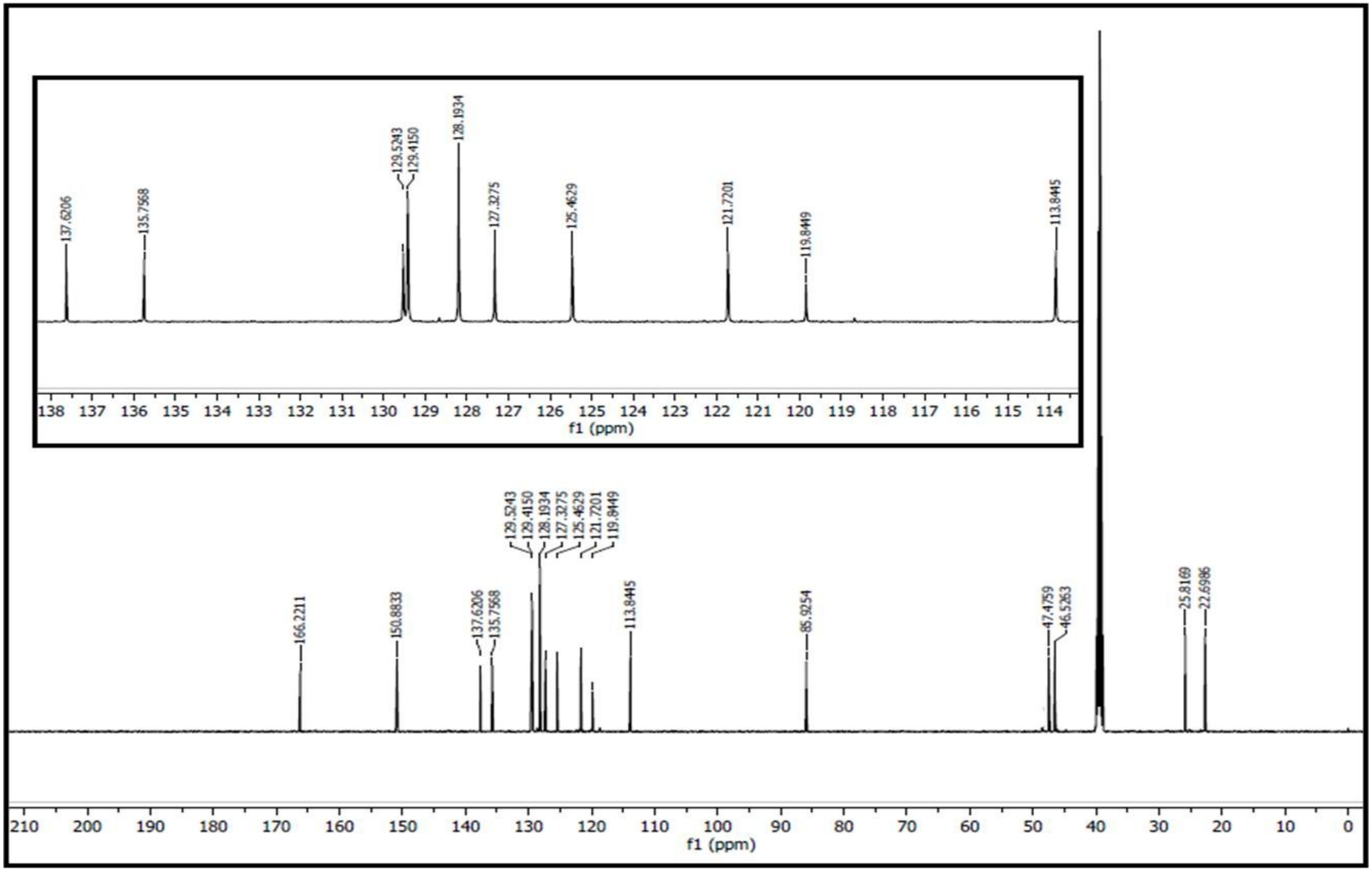

Fig. S9. ^{13}C-NMR spectrum of compound **13b** in DMSO-d_6.

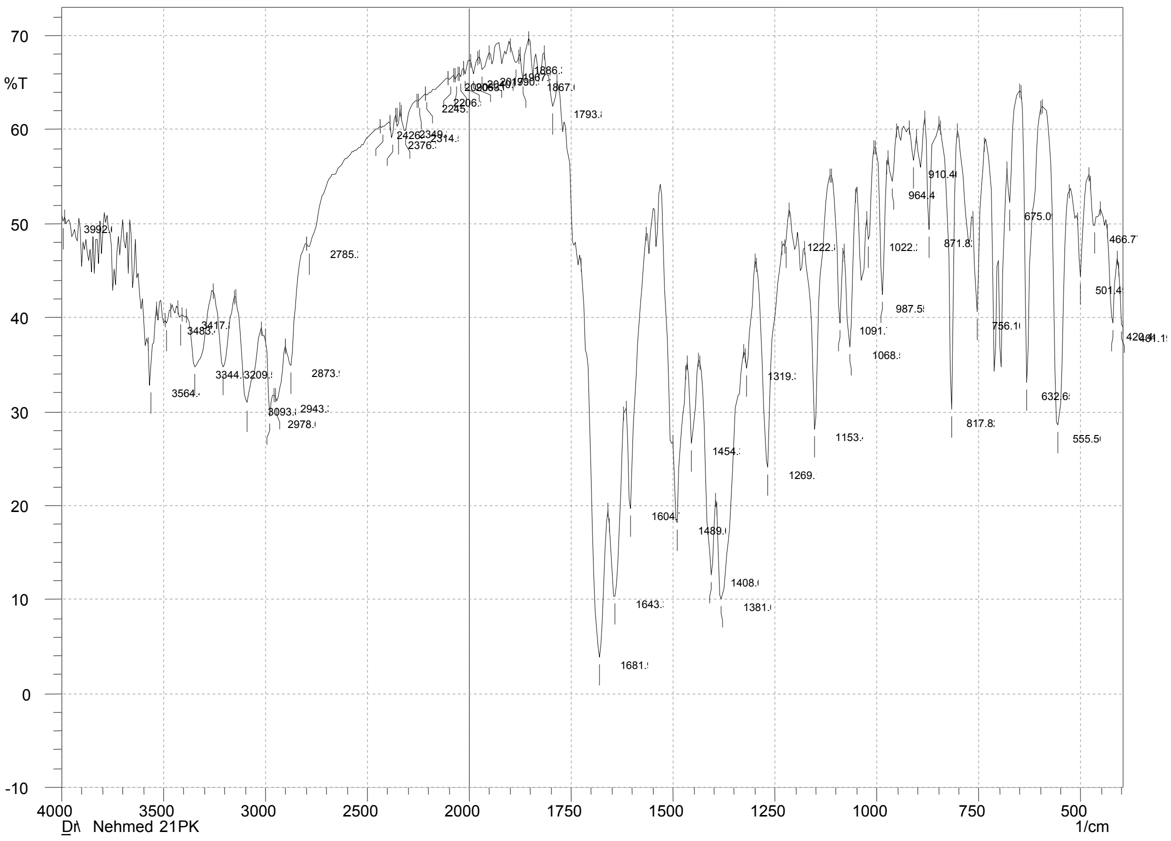

Fig. S10. IR spectrum of compound **13c** (KBr pellet).

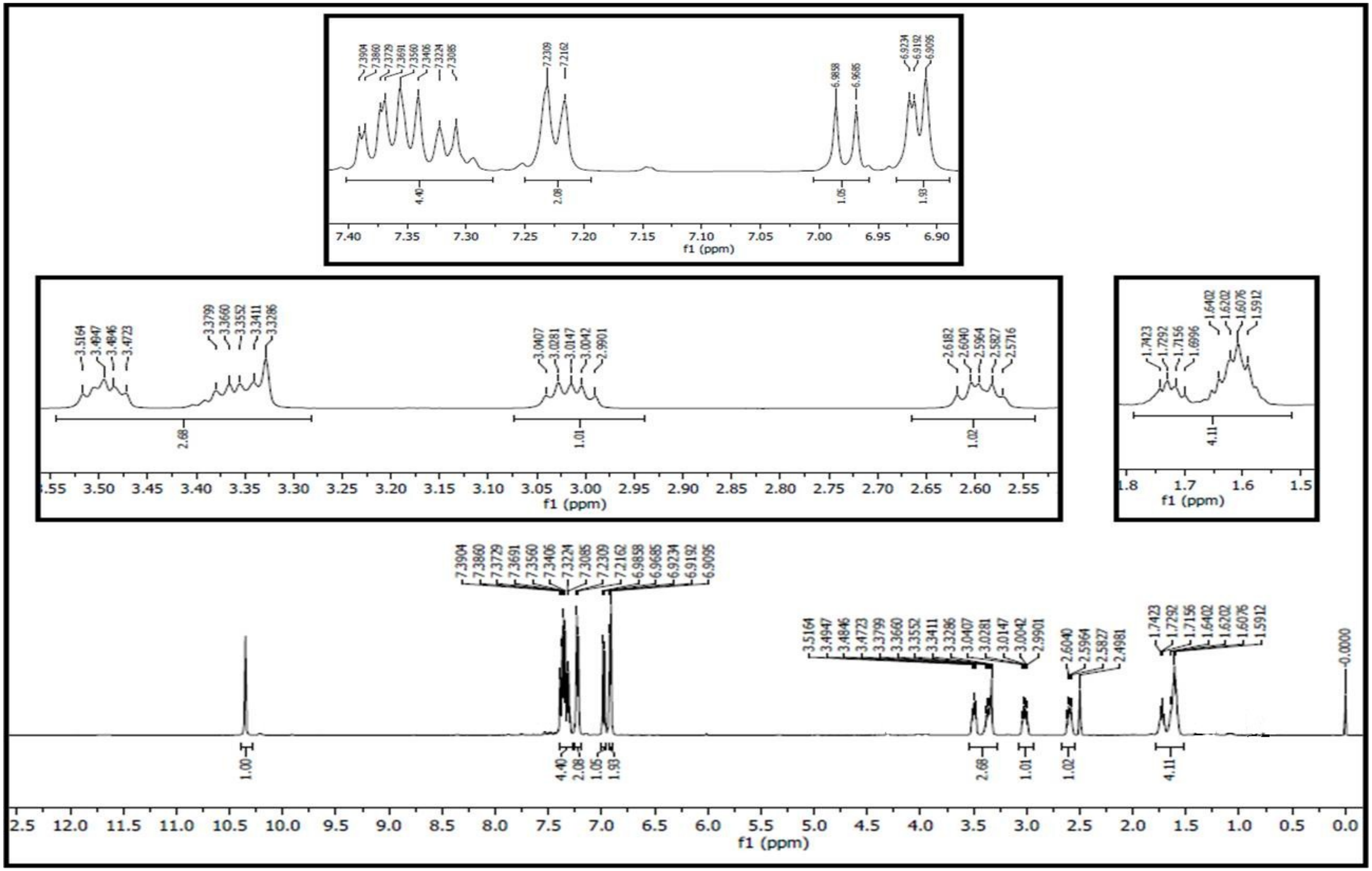

Fig. S11. ^{1}H-NMR spectrum of compound **13c** in DMSO-d_6.

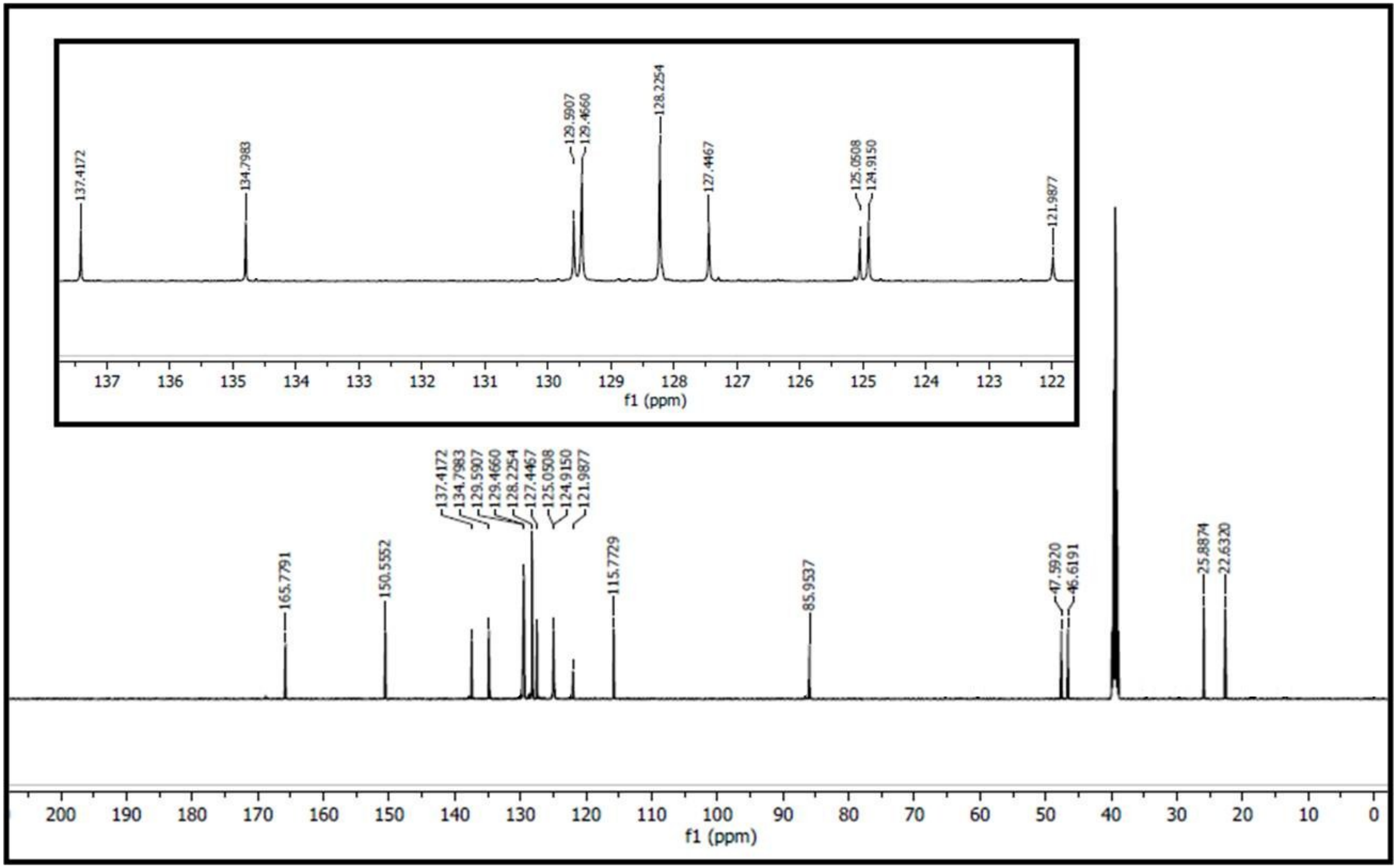

Fig. S12. ^{13}C-NMR spectrum of compound **13c** in DMSO-d_6.

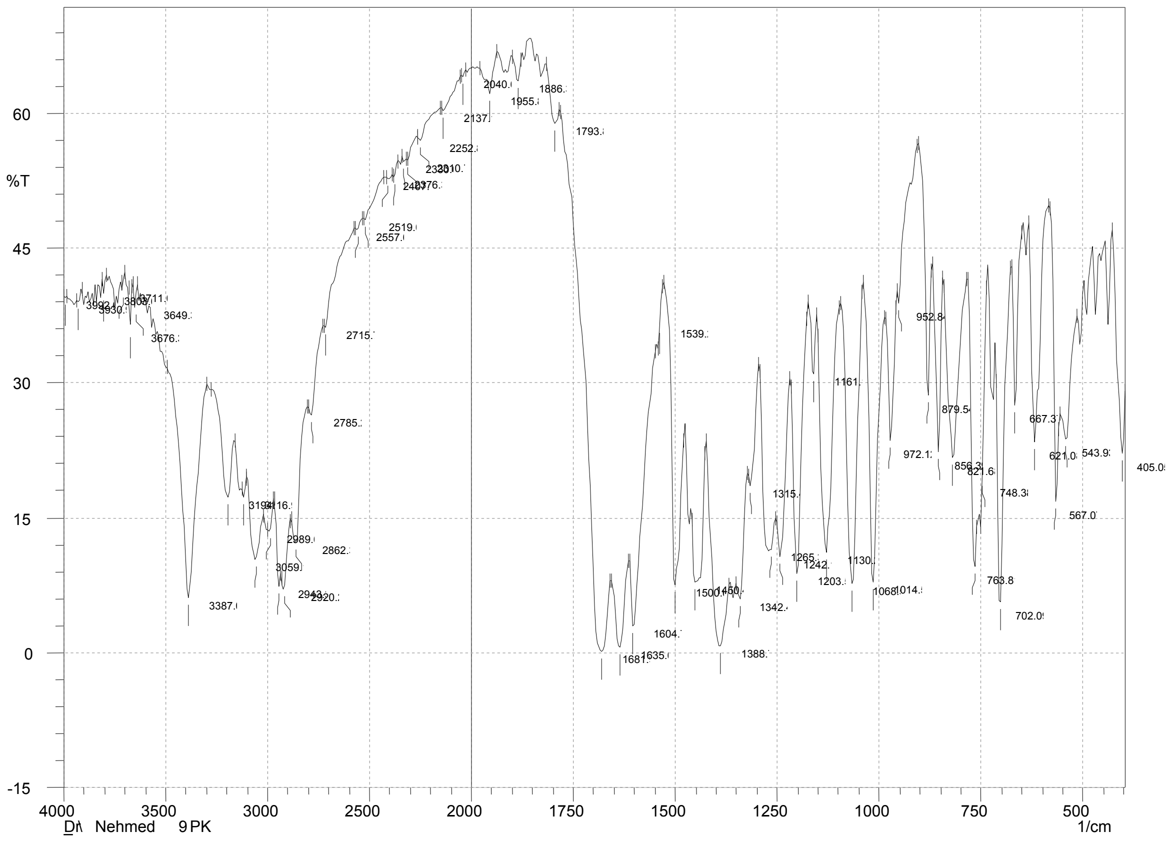

Fig. S13. IR spectrum of compound **13d** (KBr pellet).

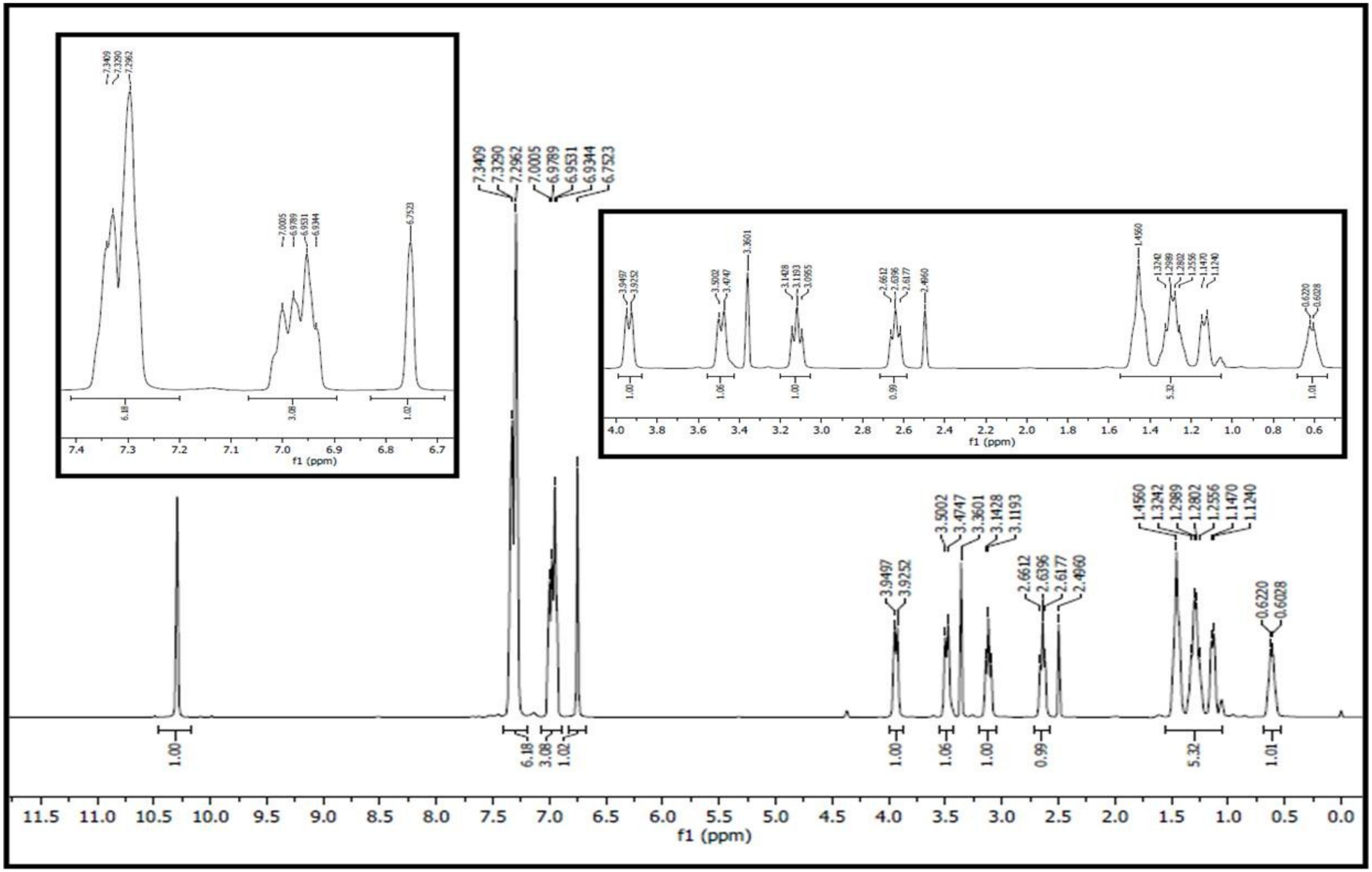

Fig. S14. ^{1}H-NMR spectrum of compound **13d** in DMSO-d_6.

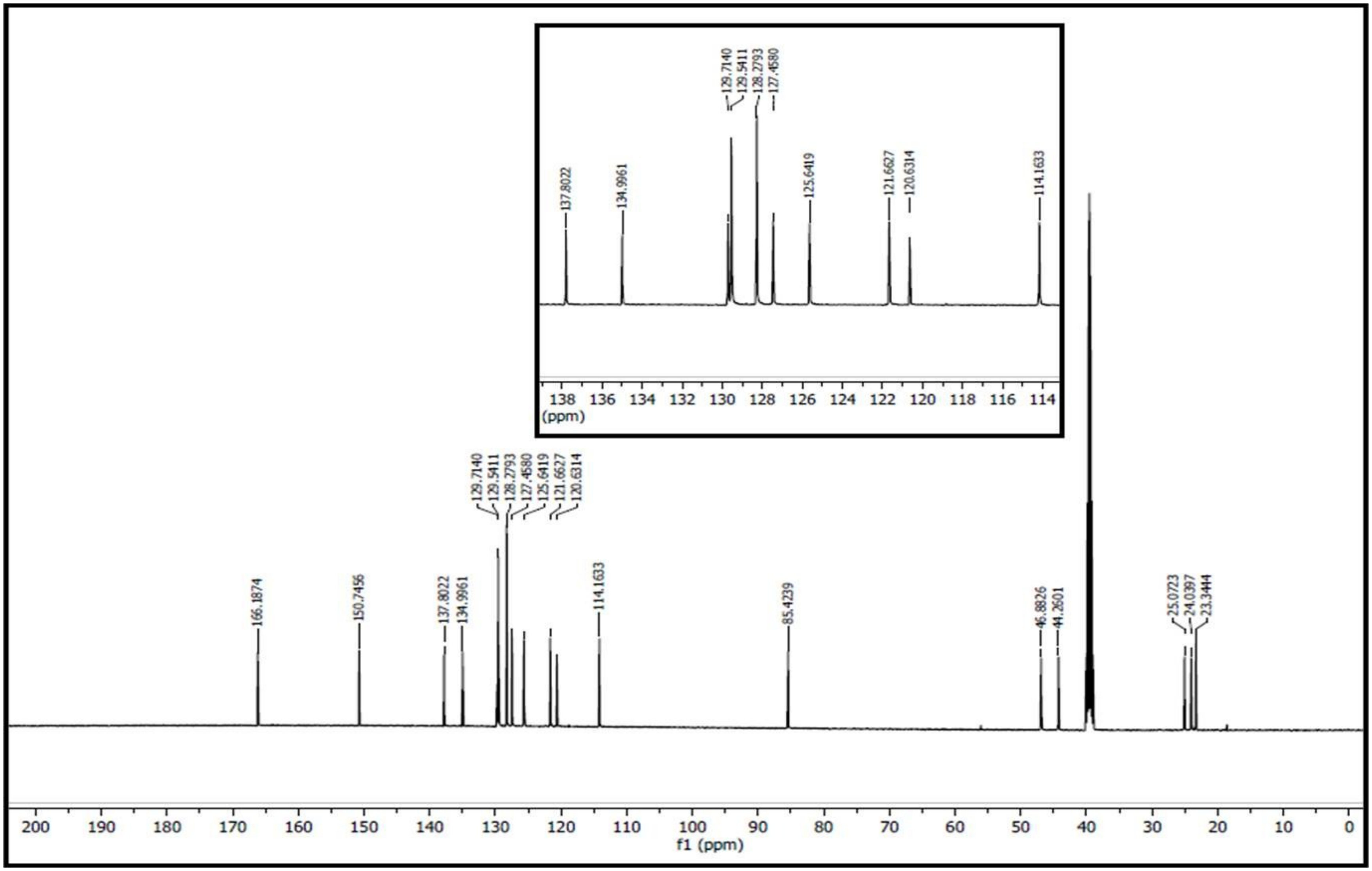

Fig. S15. ^{13}C-NMR spectrum of compound **13d** in DMSO-d_6.

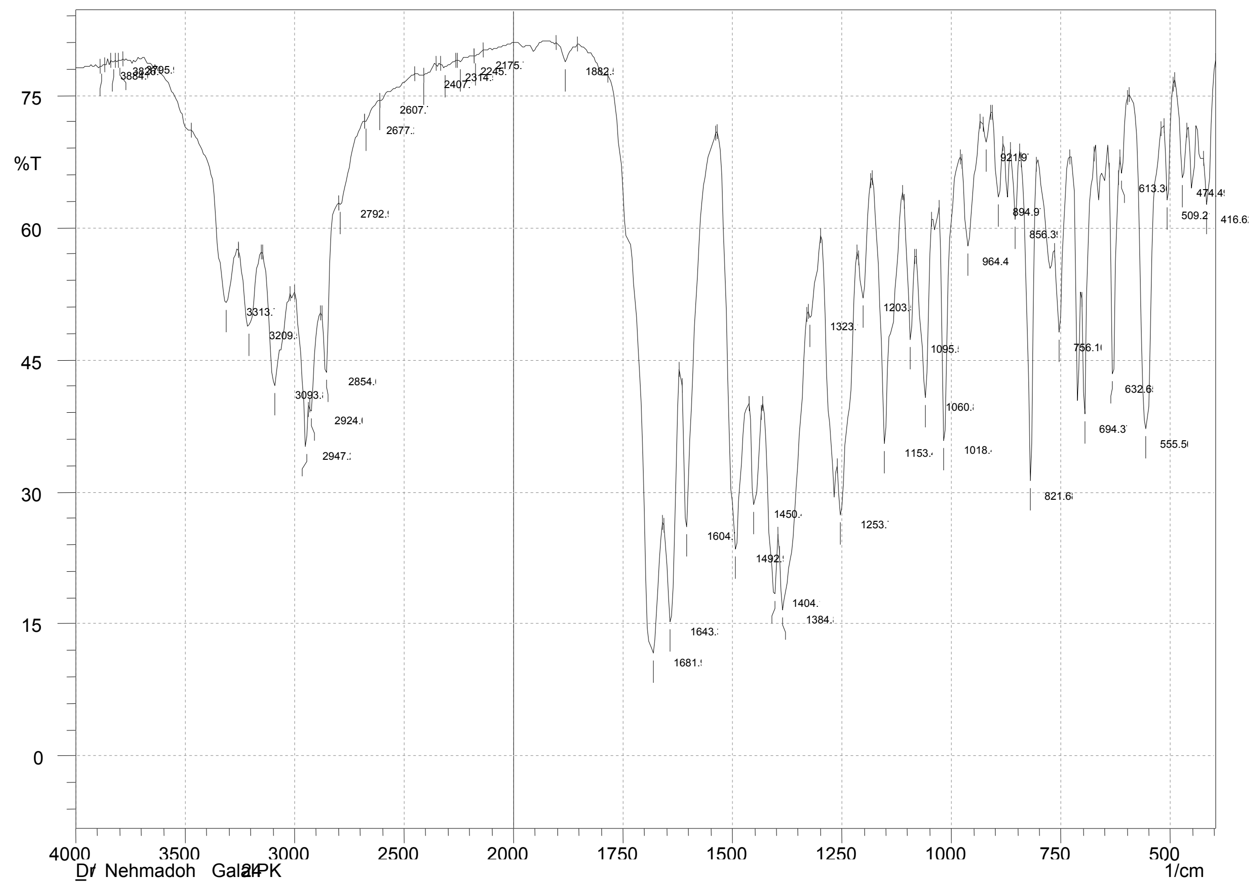

Fig. S16. IR spectrum of compound **13e** (KBr pellet).

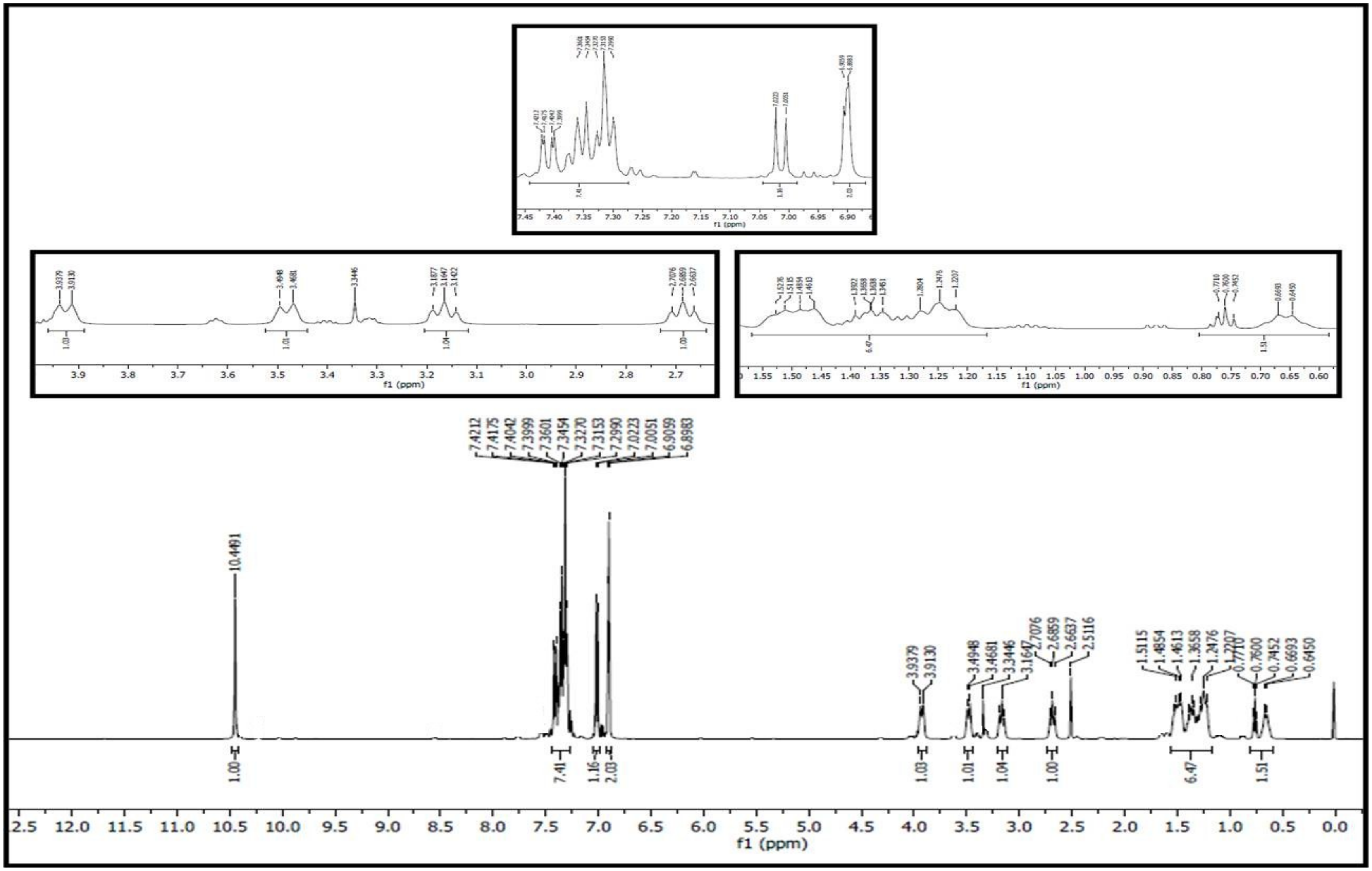

Fig. S17. ^{1}H-NMR spectrum of compound **13e** in DMSO-d_6.

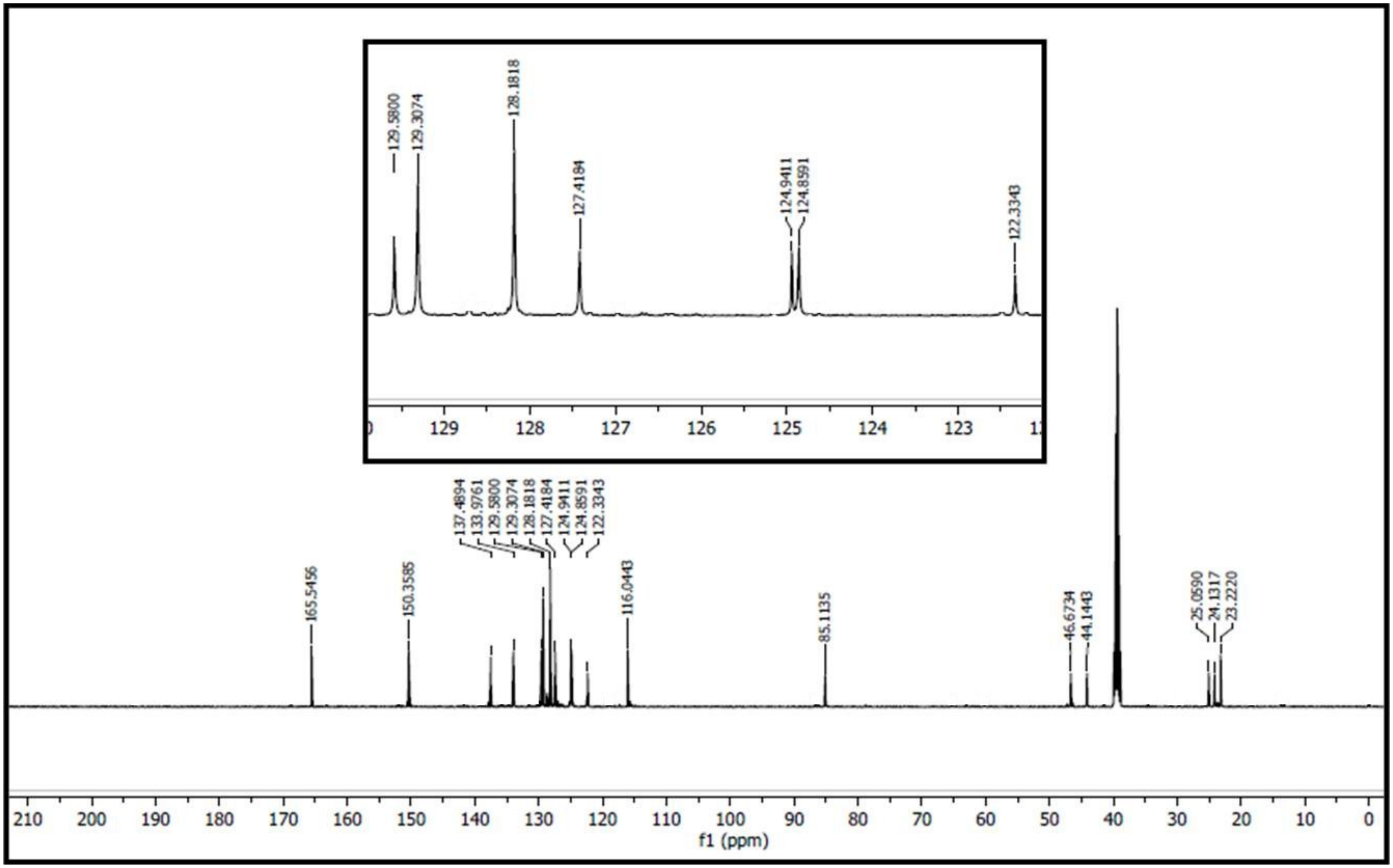

Fig. S18. ^{13}C-NMR spectrum of compound **13e** in DMSO-d_6.

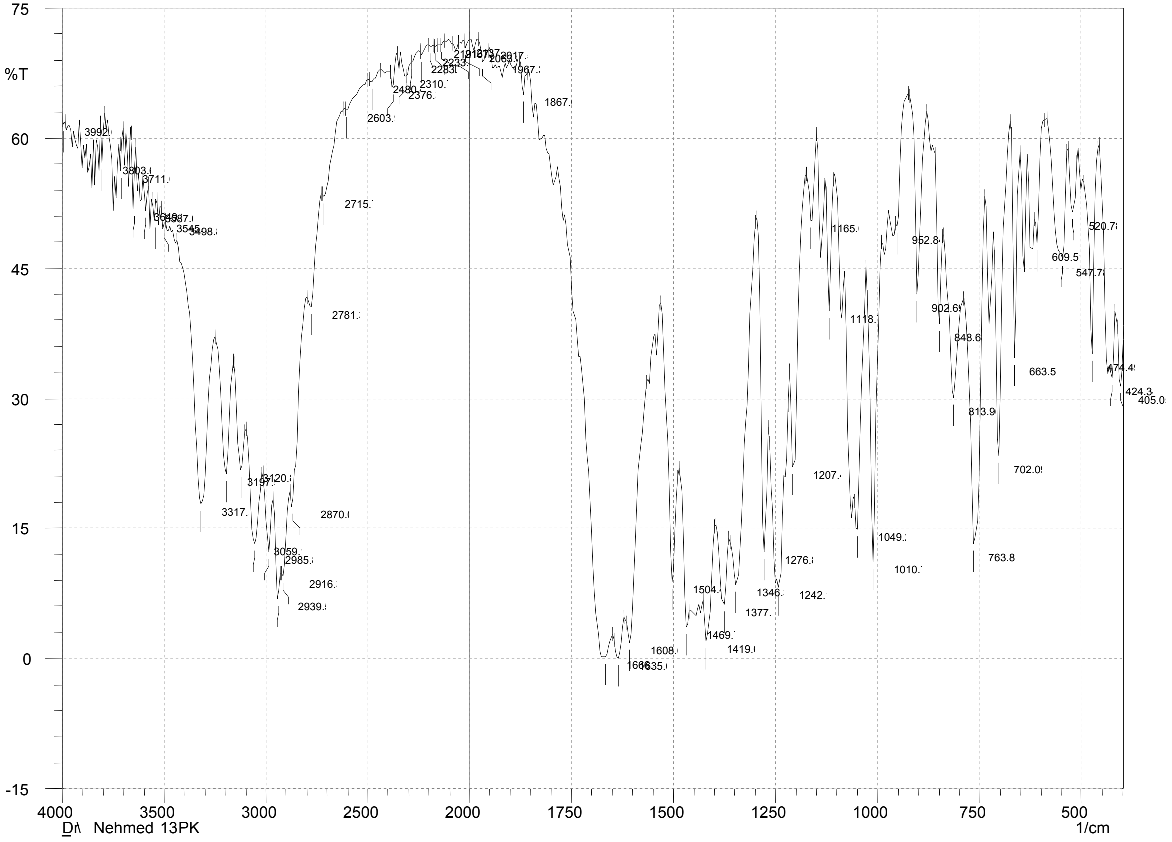

Fig. S19. IR spectrum of compound **13f** (KBr pellet).

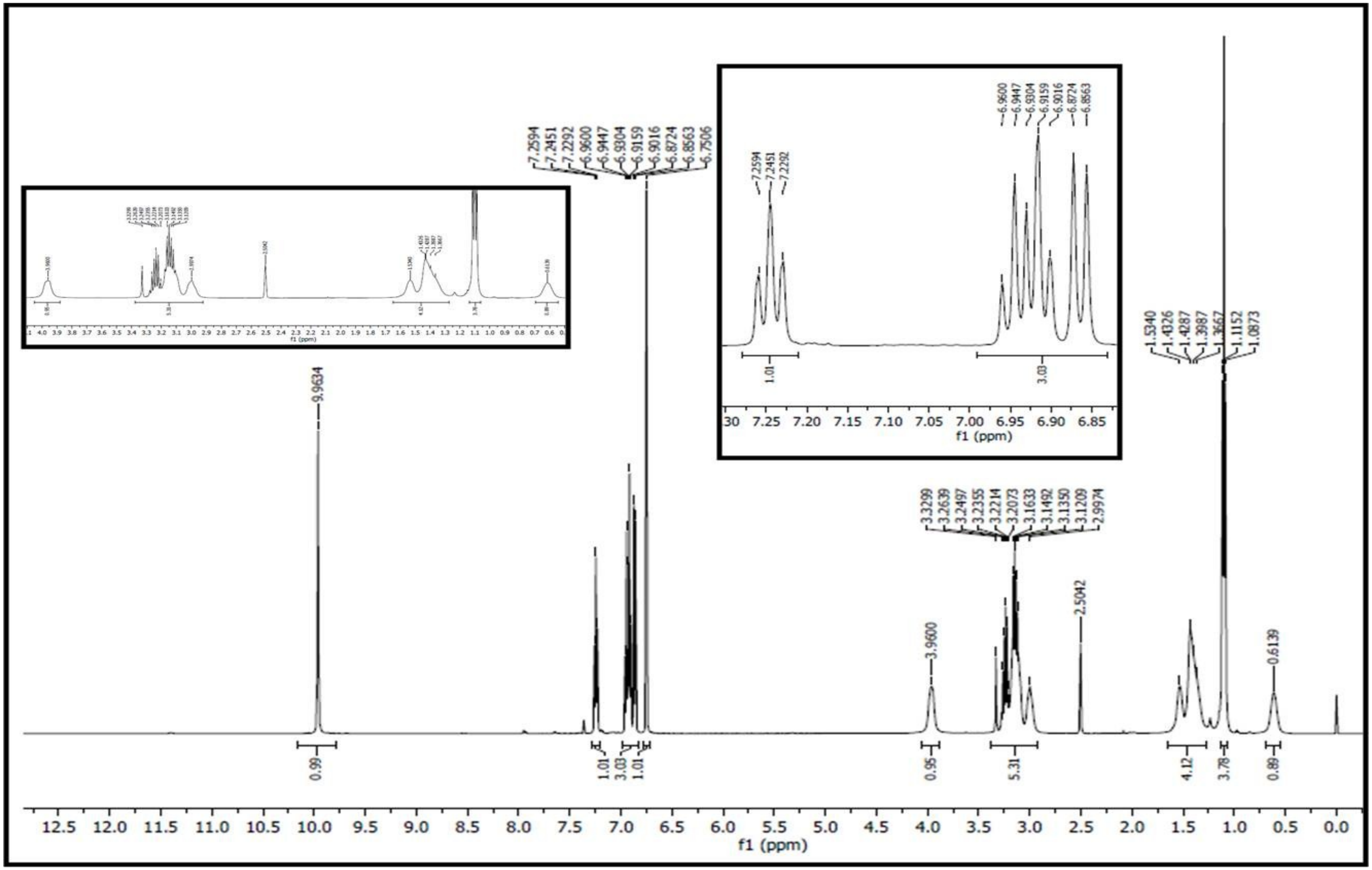

Fig. S20. ^{1}H-NMR spectrum of compound **13f** in DMSO-d_6.

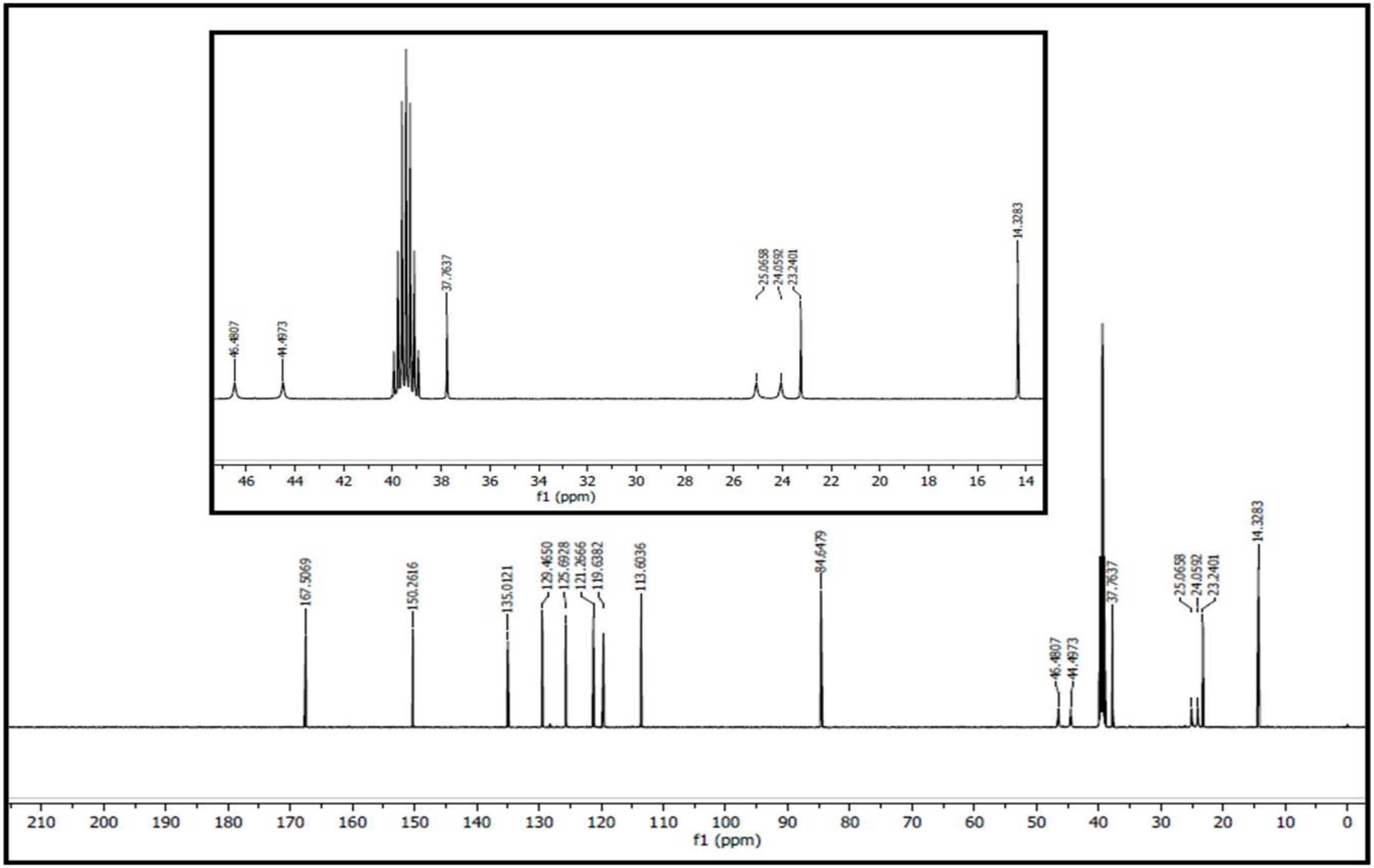

Fig. S21. ^{13}C-NMR spectrum of compound **13f** in DMSO-d_6.

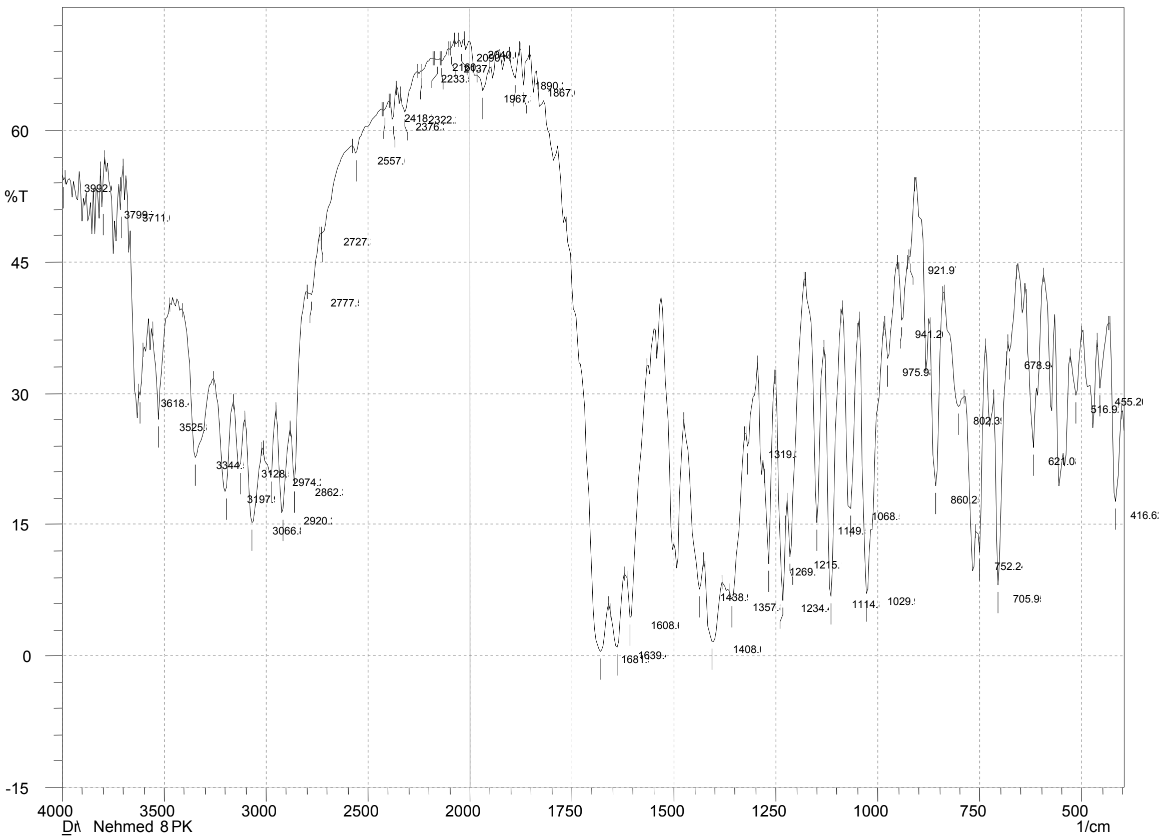

Fig. S22. IR spectrum of compound **13g** (KBr pellet).

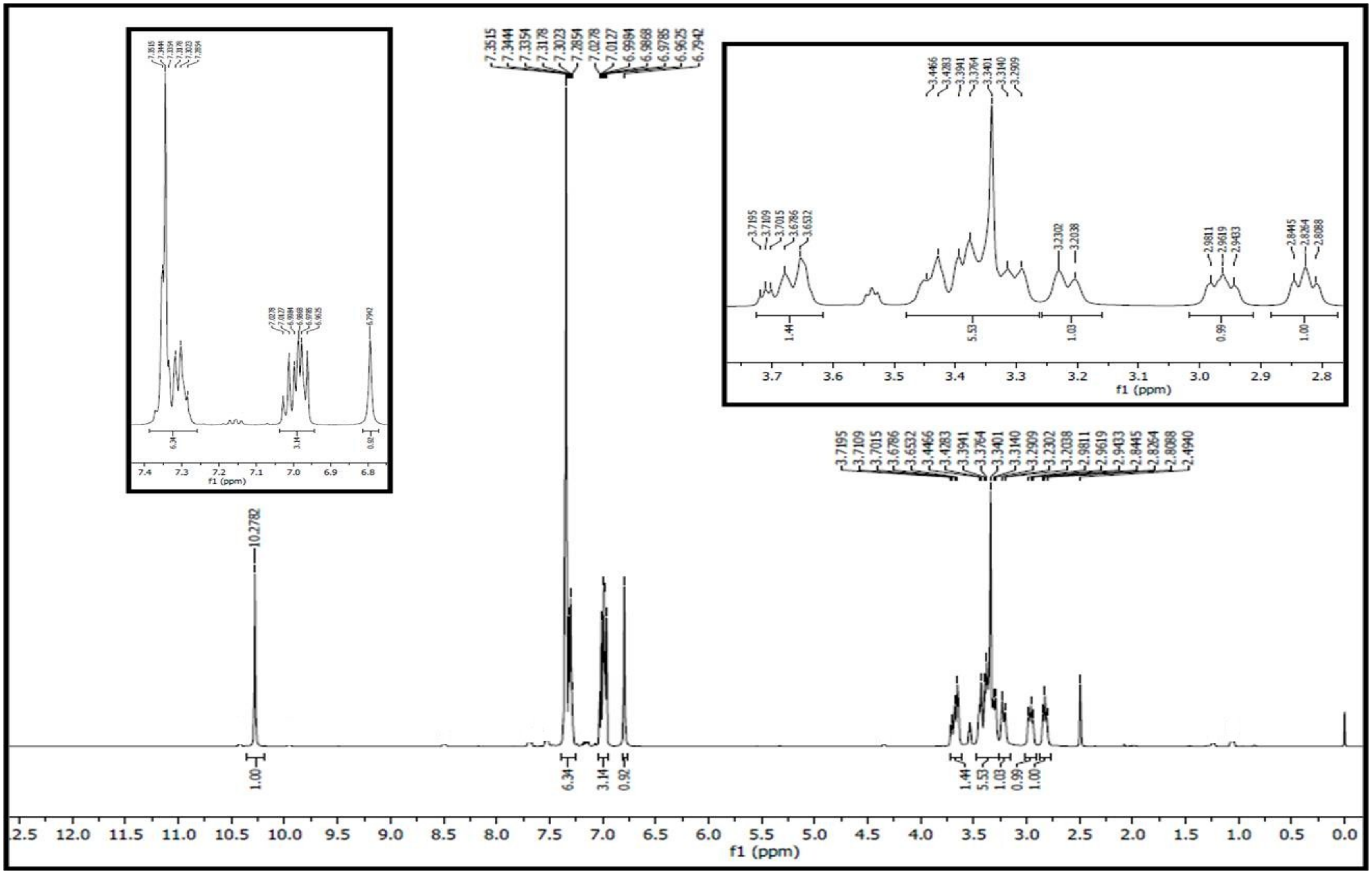

Fig. S23. ^{1}H-NMR spectrum of compound **13g** in DMSO-d_6.

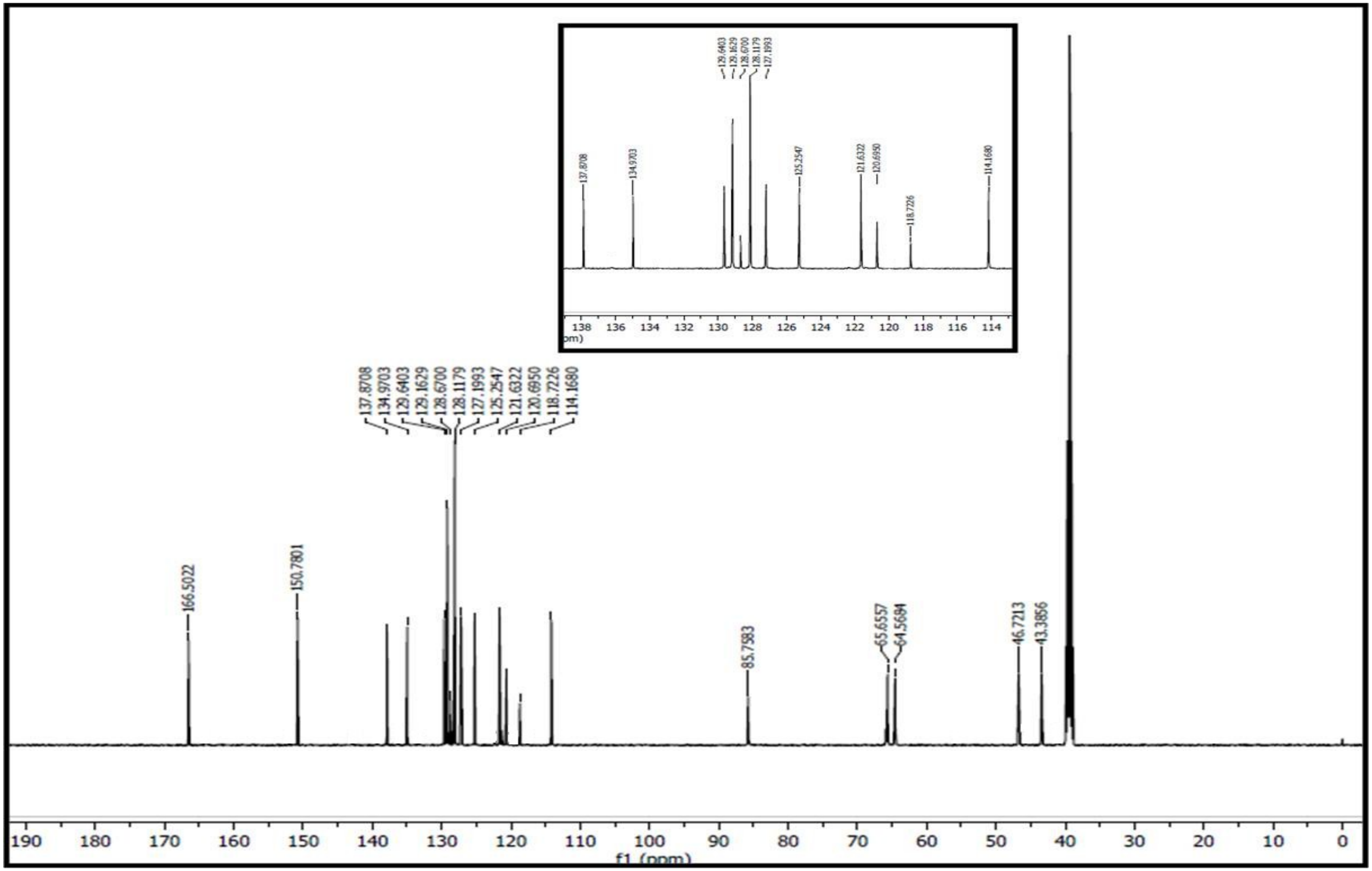

Fig. S24. ^{13}C-NMR spectrum of compound **13g** in DMSO-d_6.

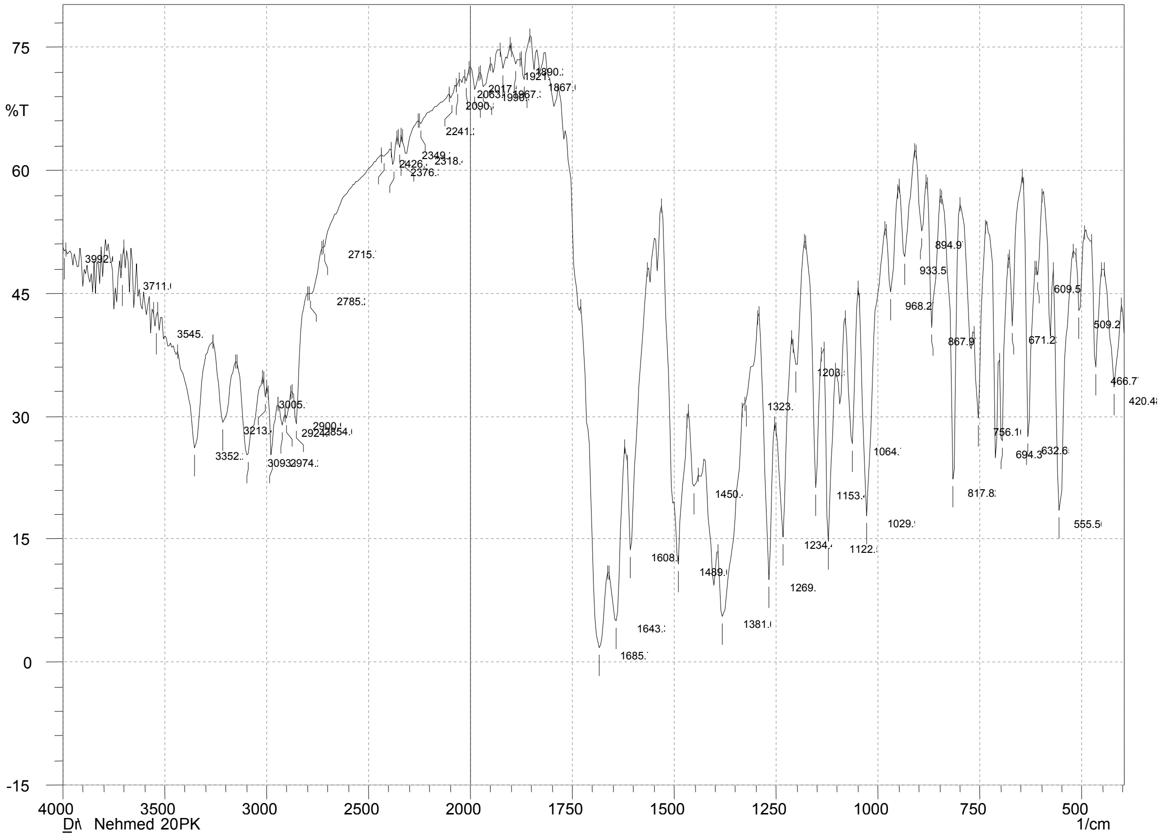

Fig. S25. IR spectrum of compound **13h** (KBr pellet).

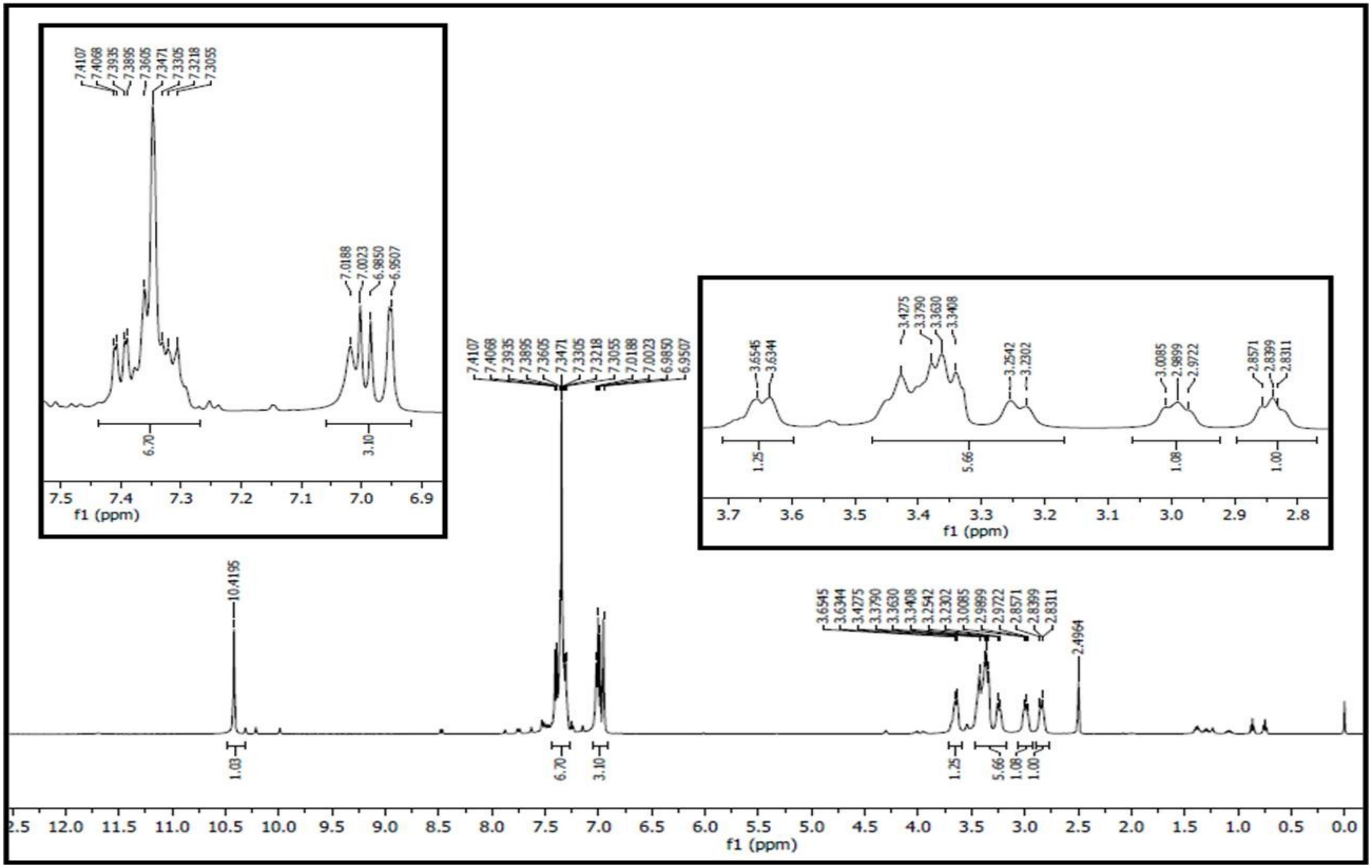

Fig. S26. ^{1}H-NMR spectrum of compound **13h** in DMSO-d_6.

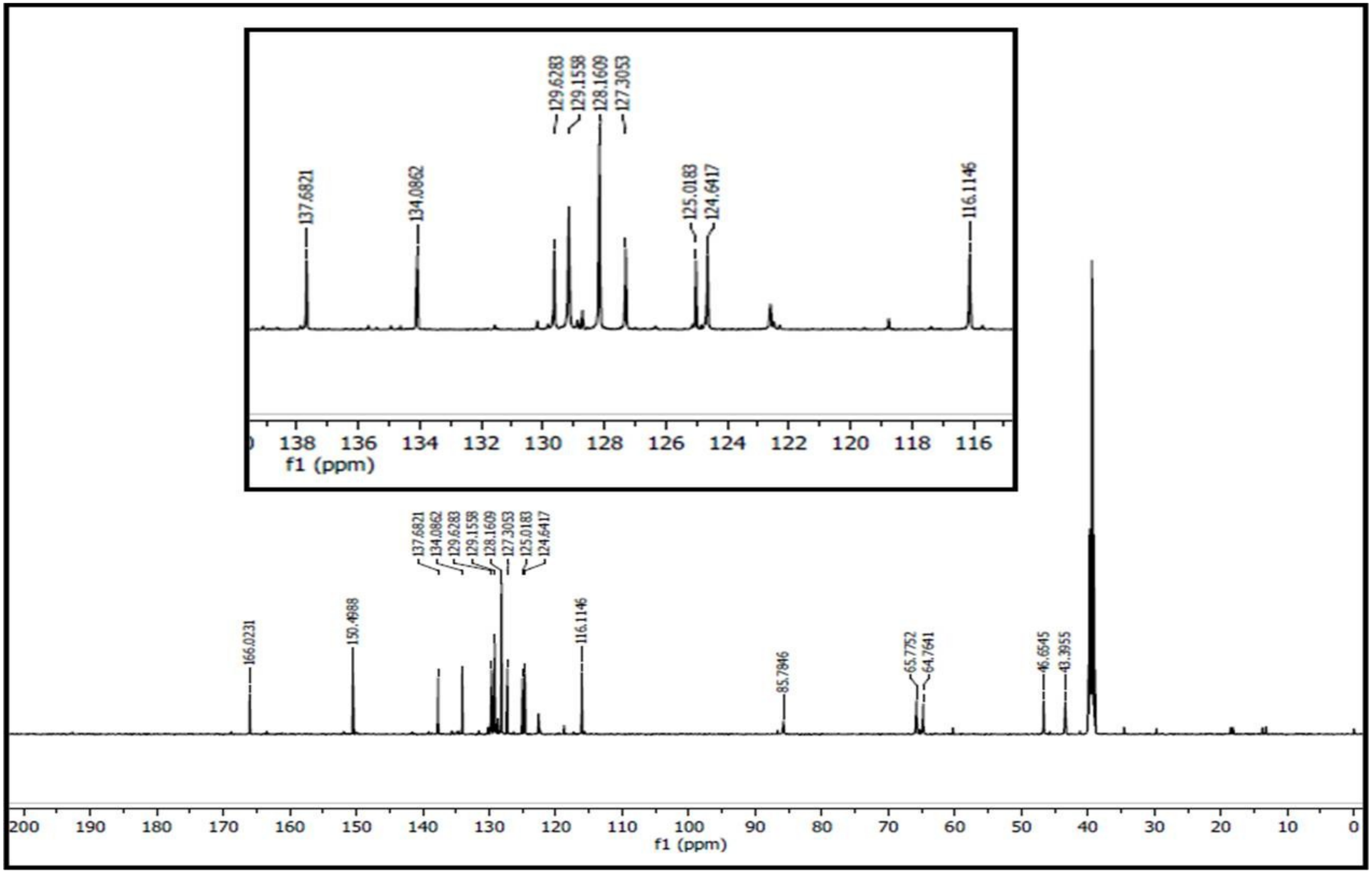

Fig. S27. ^{13}C-NMR spectrum of compound **13h** in DMSO-d_6.

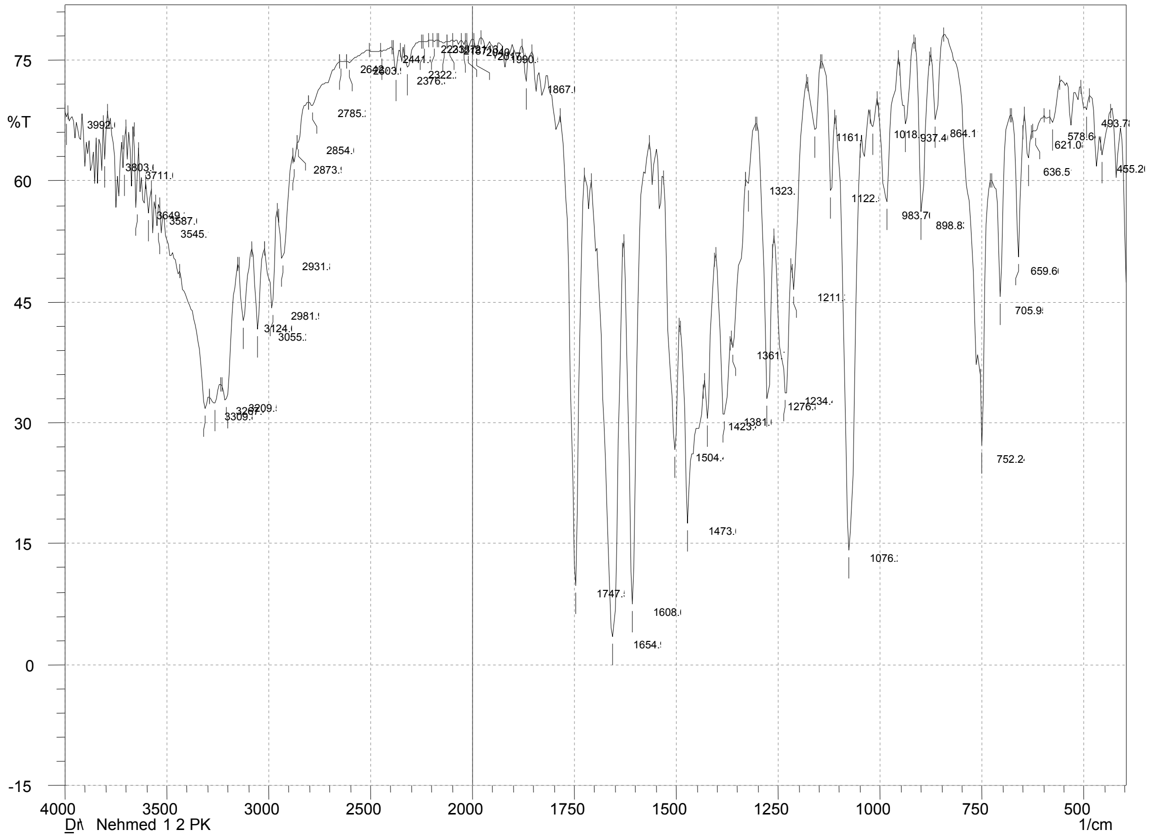

Fig. S28. IR spectrum of compound **13i** (KBr pellet).

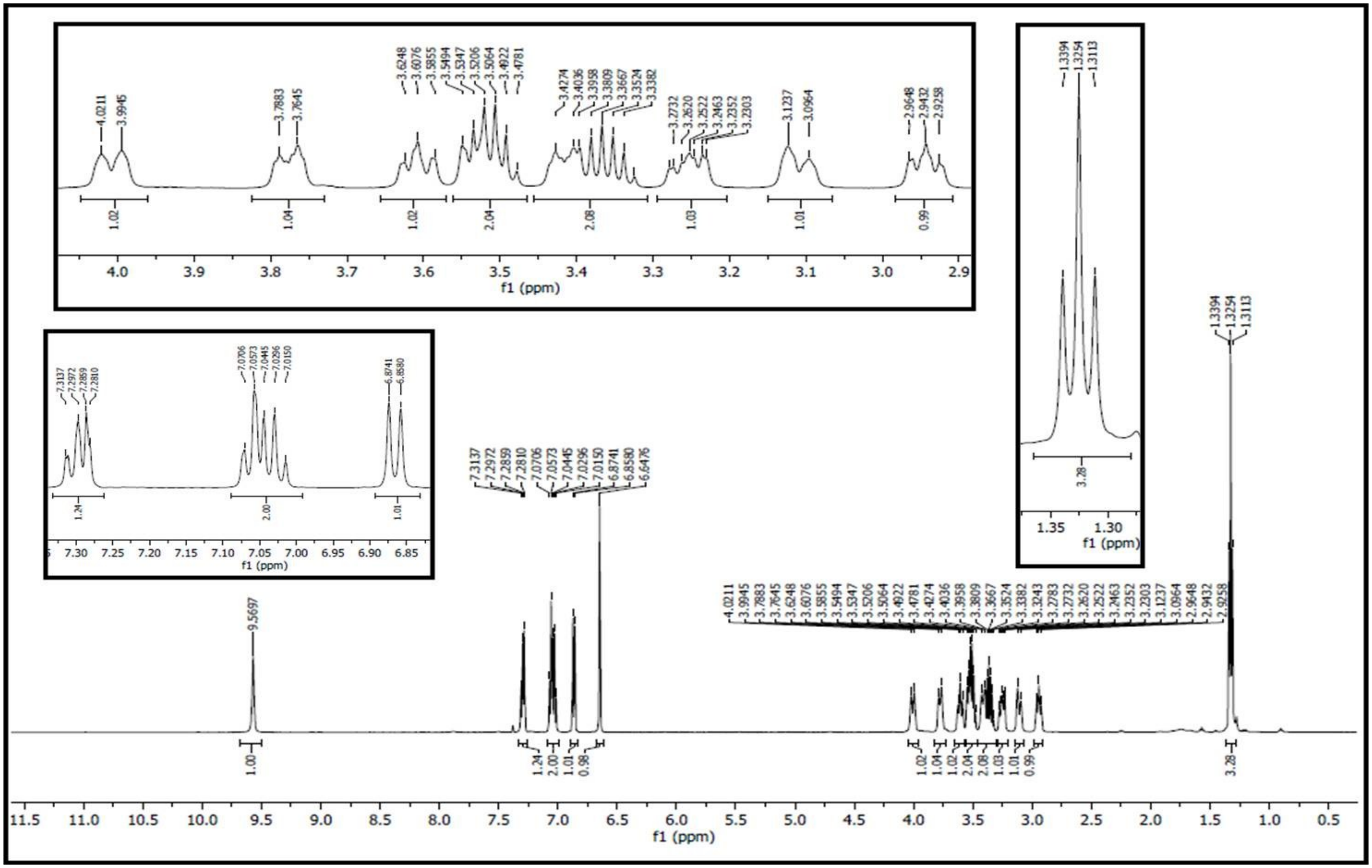

Fig. S29. ^{1}H-NMR spectrum of compound **13i** in $CDCl_3$.

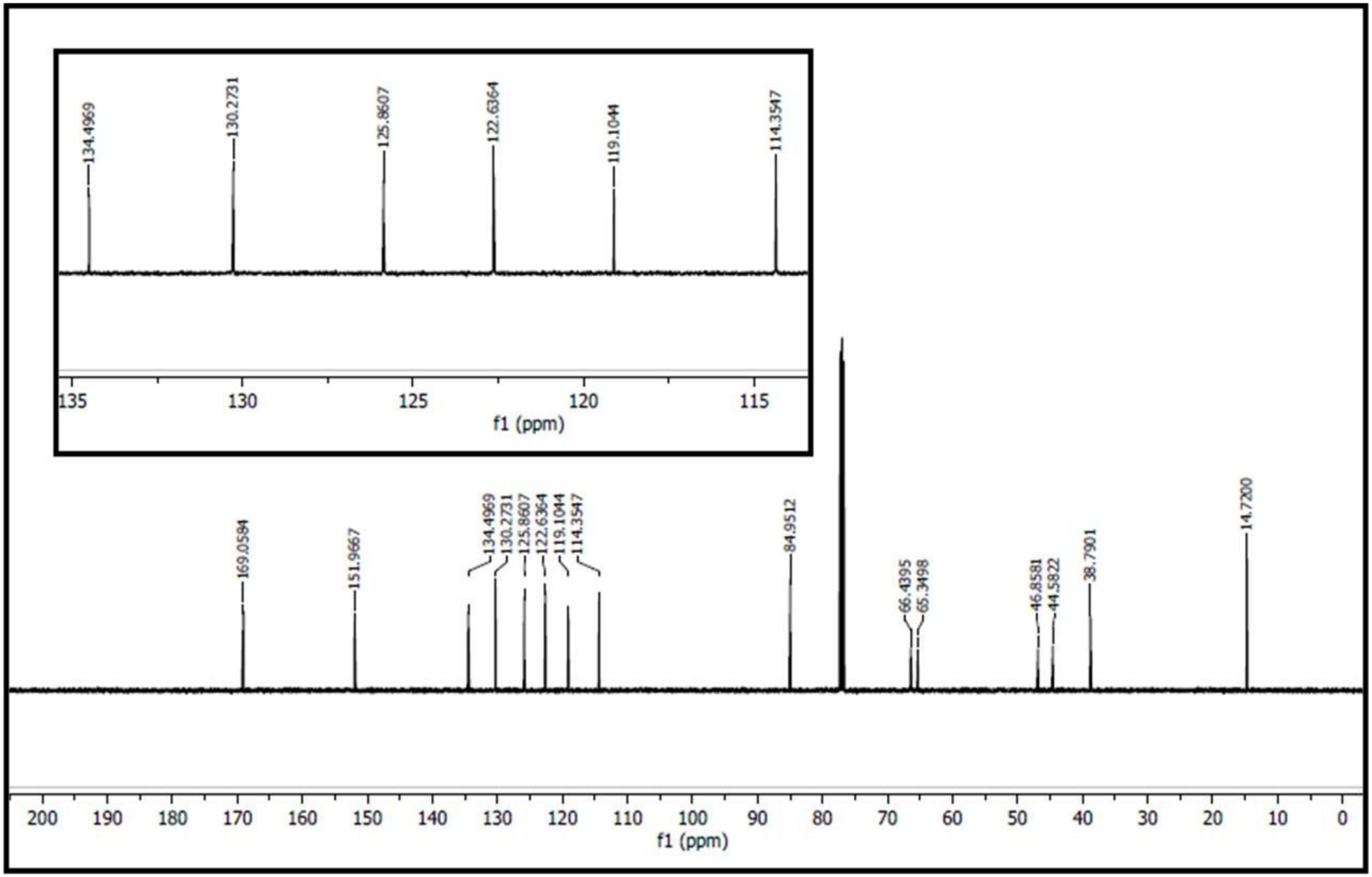

Fig. S30. ^{13}C-NMR spectrum of compound **13i** in $CDCl_3$.

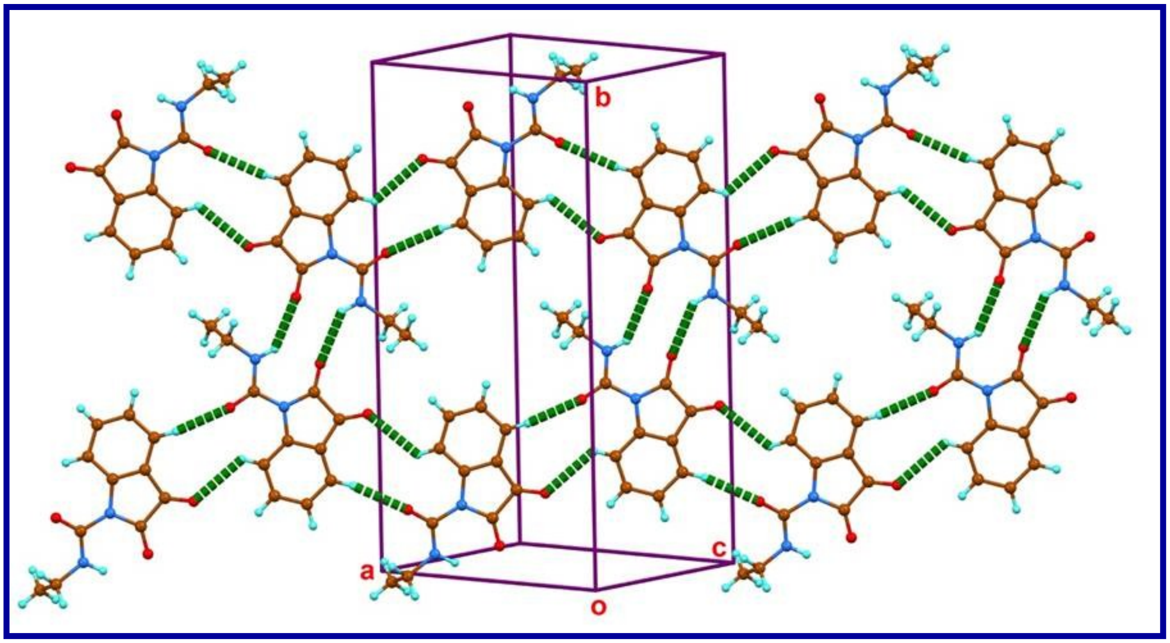

Fig. S31. A view of the unit cell contents for **10c** showing the H-bonds as dashed lines.

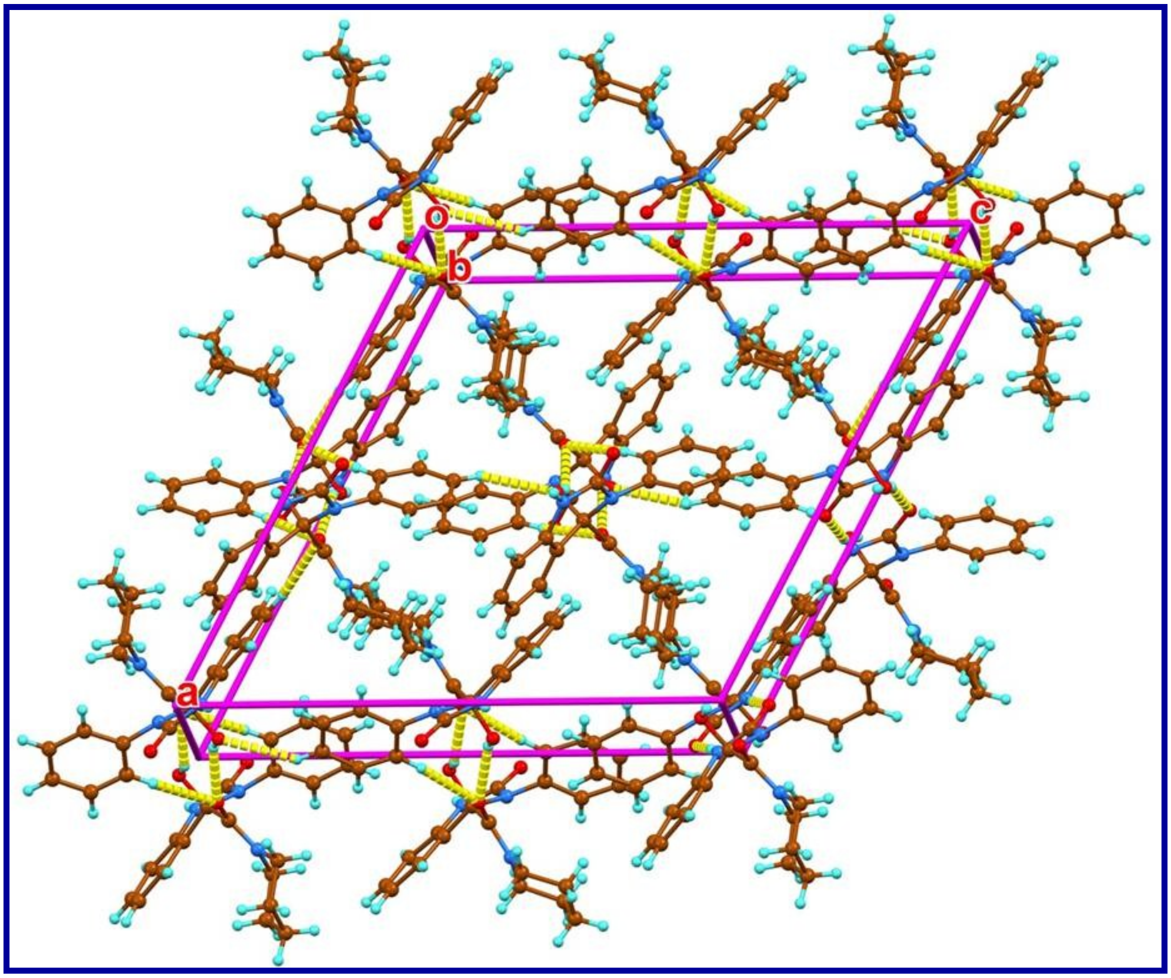

Fig. S32. A view of the unit cell contents for **13d** showing the H-bonds as dashed lines.

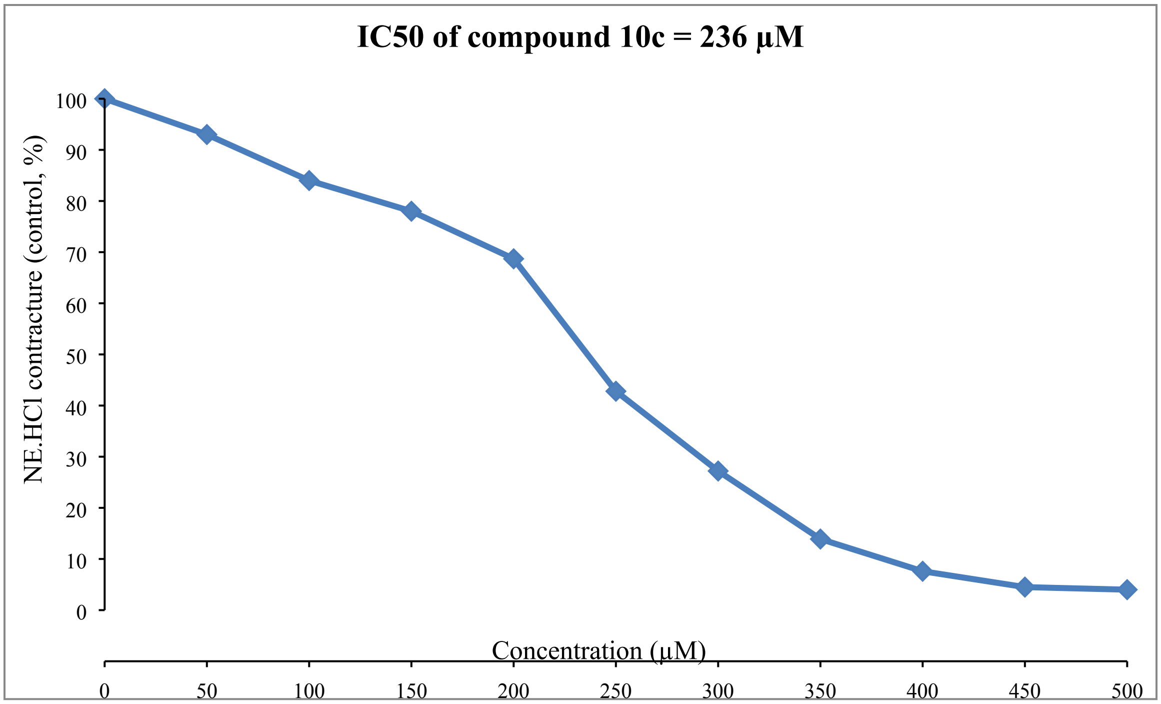
IC50 of compound 10c = 236 μM
NE.HCl contracture (control, %)
100
90
80
70
60
50
40
30
20
10
0
Concentration (μM)
0
50
100
150
200
250
300
350
400
450
500

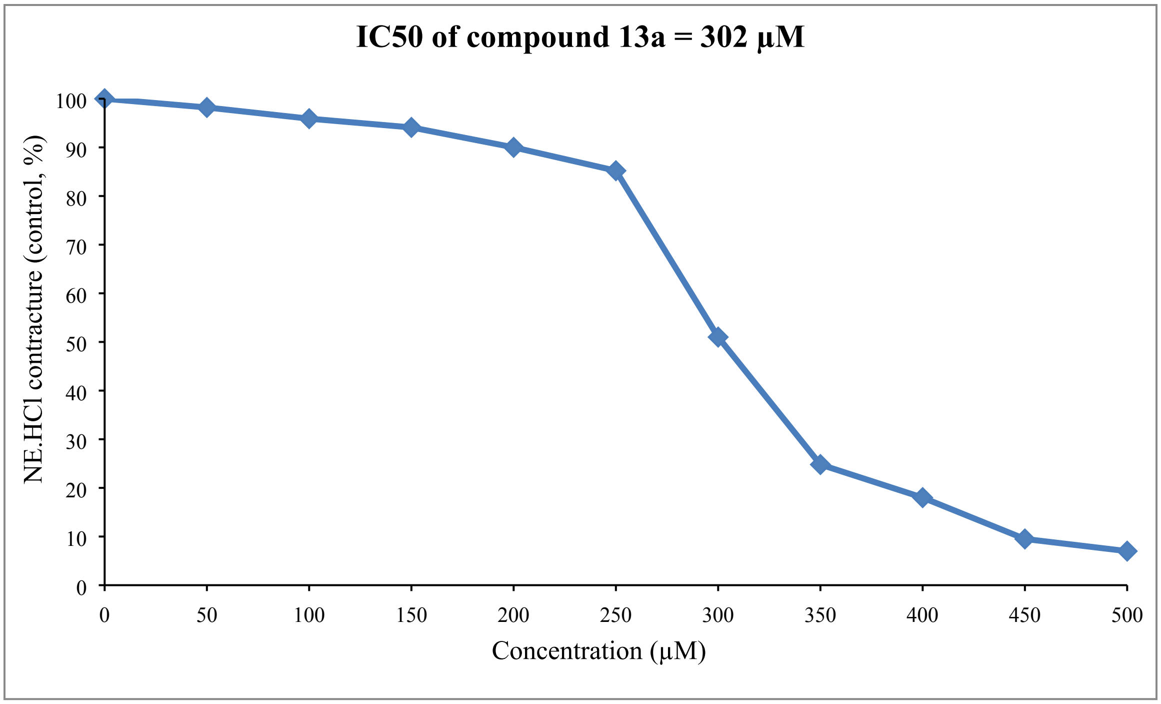
IC50 of compound 13a = 302 μM
NE.HCl contracture (control, %)
100
90
80
70
60
50
40
30
20
10
0
0
50
100
150
200
250
300
350
400
450
500
Concentration (μM)

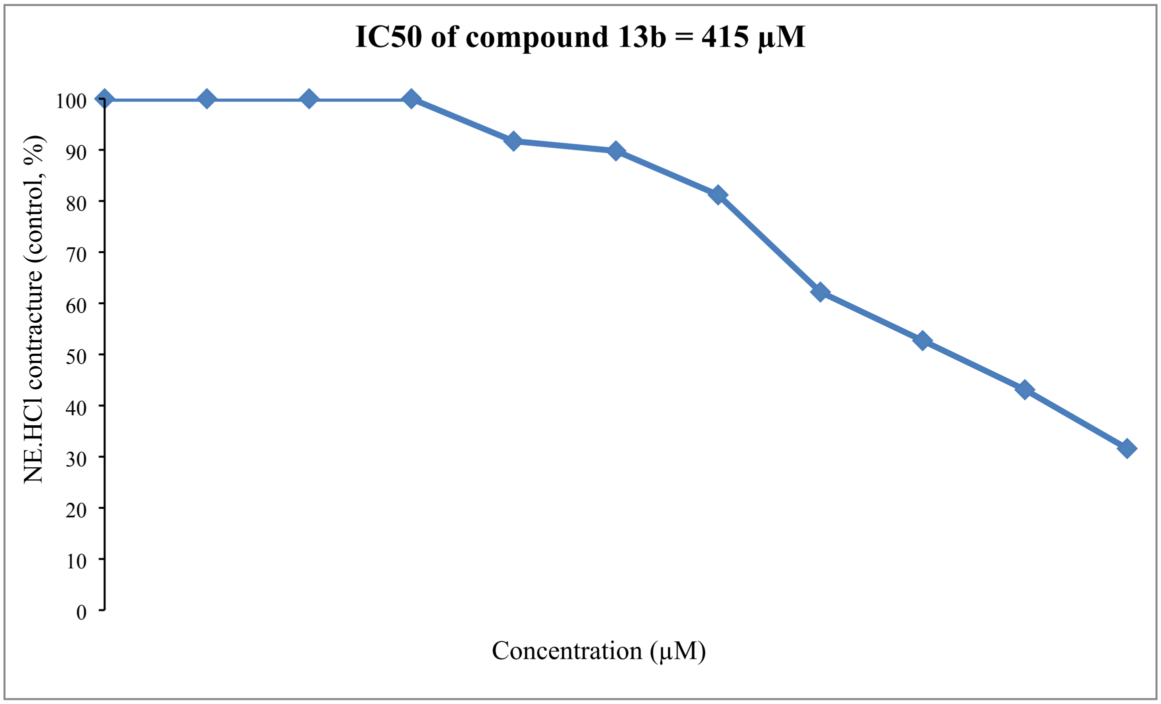
IC50 of compound 13b = 415 µM
NE.HCl contracture (control, %)
100
90
80
70
60
50
40
30
20
10
0
Concentration (µM)

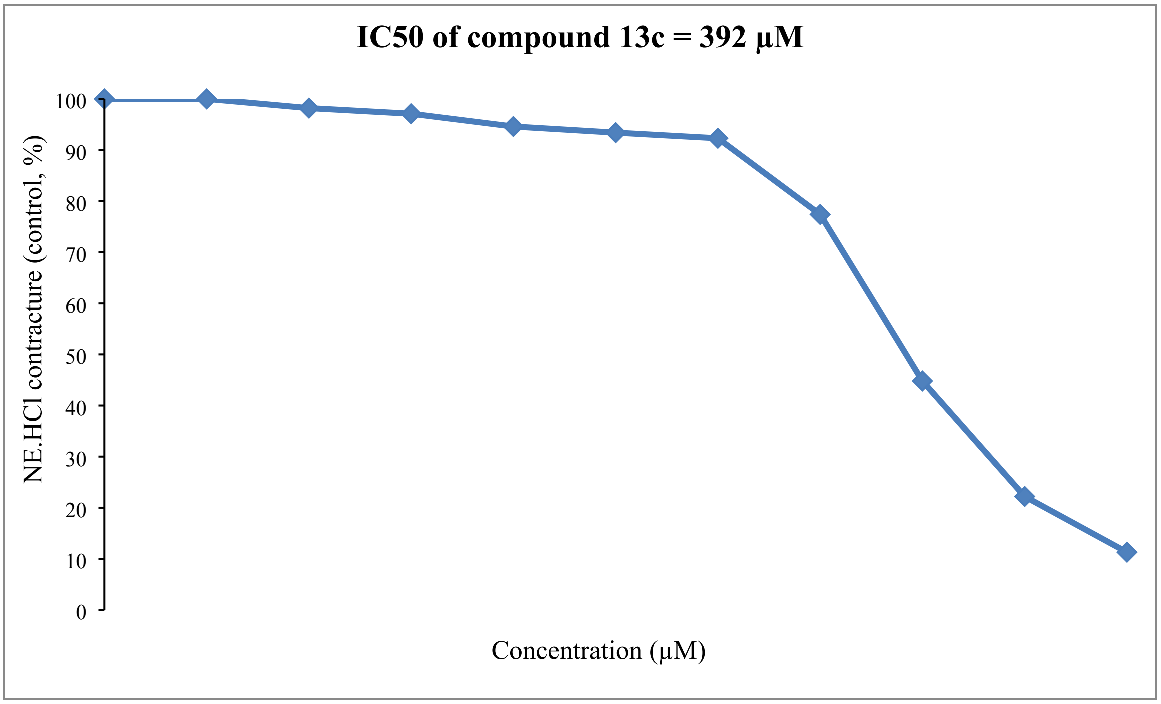
IC50 of compound 13c = 392 µM
NE.HCl contracture (control, %)
100
90
80
70
60
50
40
30
20
10
0
Concentration (µM)

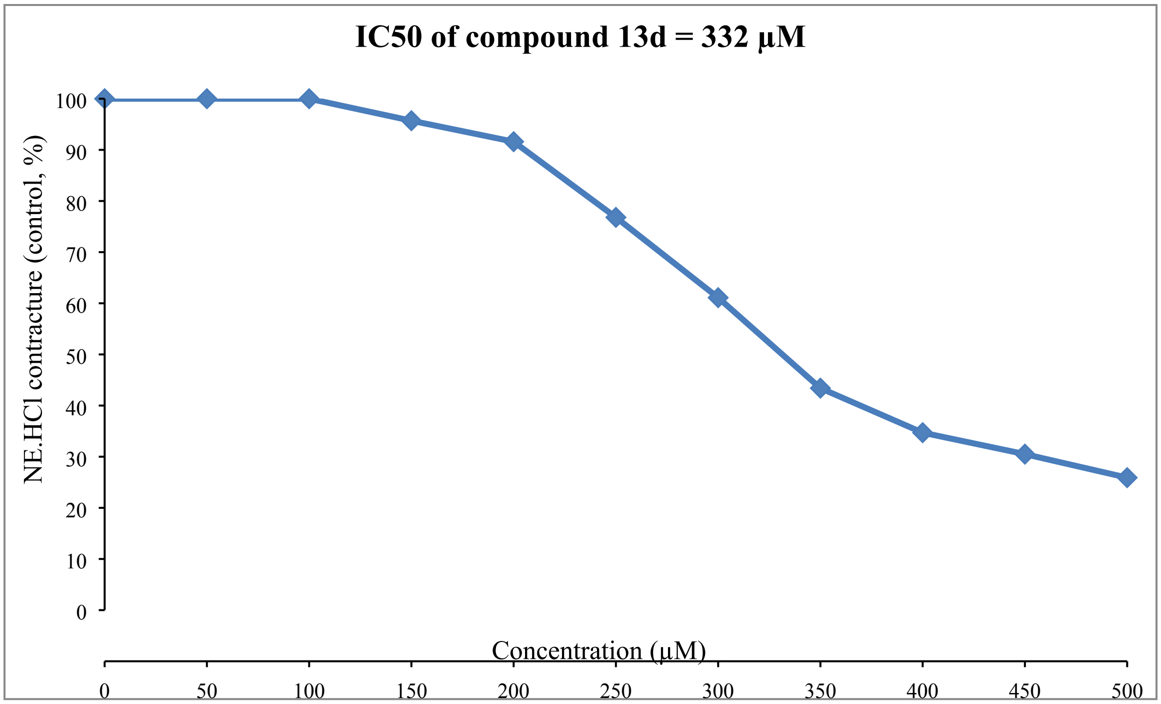
IC50 of compound 13d = 332 µM
NE.HCl contracture (control, %)
0
10
20
30
40
50
60
70
80
90
100
Concentration (µM)
0
50
100
150
200
250
300
350
400
450
500

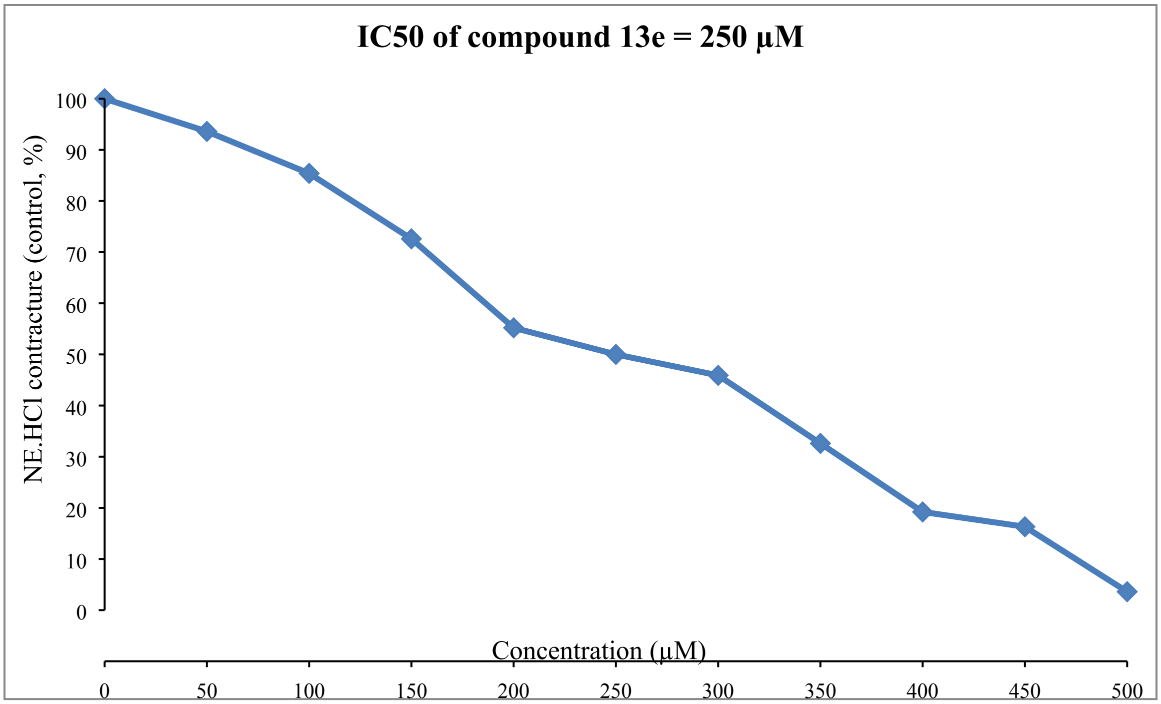
IC50 of compound 13e = 250 µM
NE.HCl contracture (control, %)
0
10
20
30
40
50
60
70
80
90
100
Concentration (µM)
0
50
100
150
200
250
300
350
400
450
500

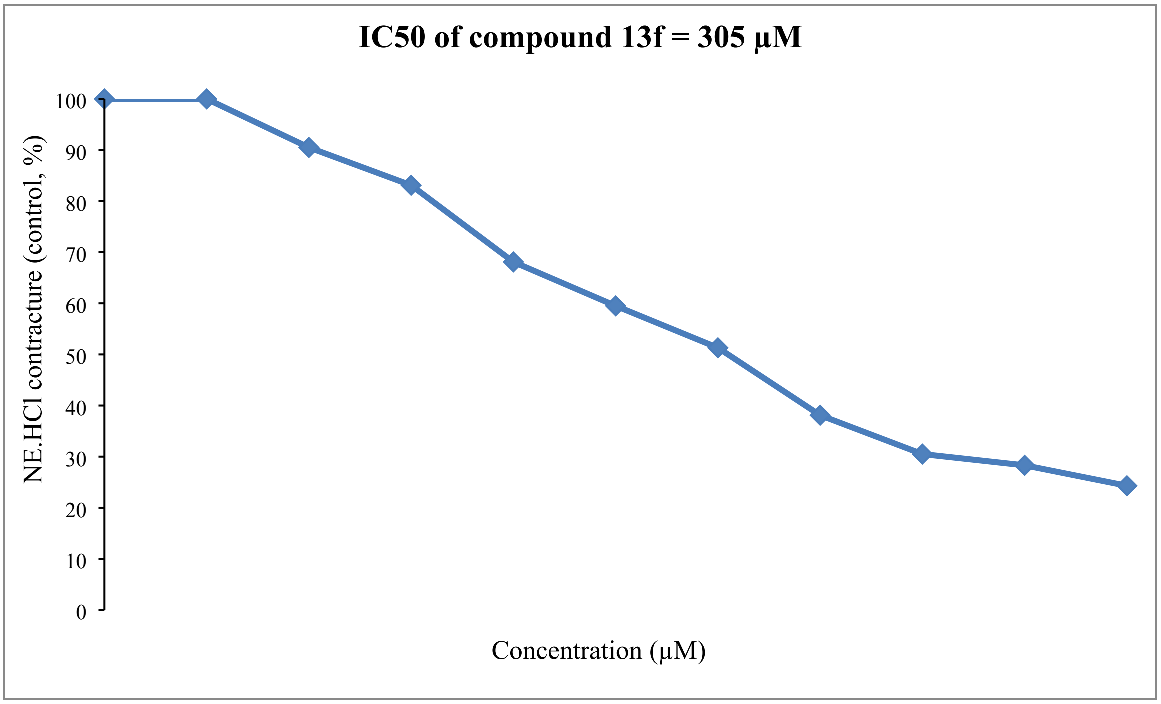
IC50 of compound 13f = 305 µM
NE.HCl contracture (control, %)
100
90
80
70
60
50
40
30
20
10
0
Concentration (µM)

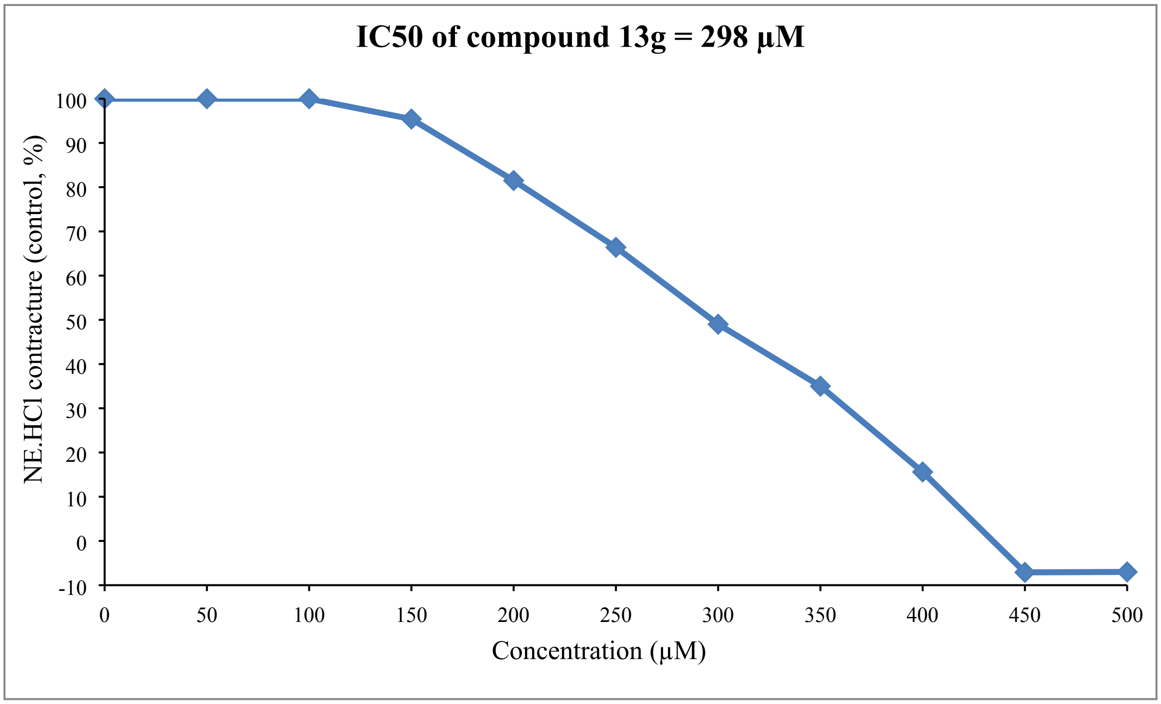
IC50 of compound 13g = 298 µM
NE.HCl contracture (control, %)
100
90
80
70
60
50
40
30
20
10
0
-10
0
50
100
150
200
250
300
350
400
450
500
Concentration (µM)

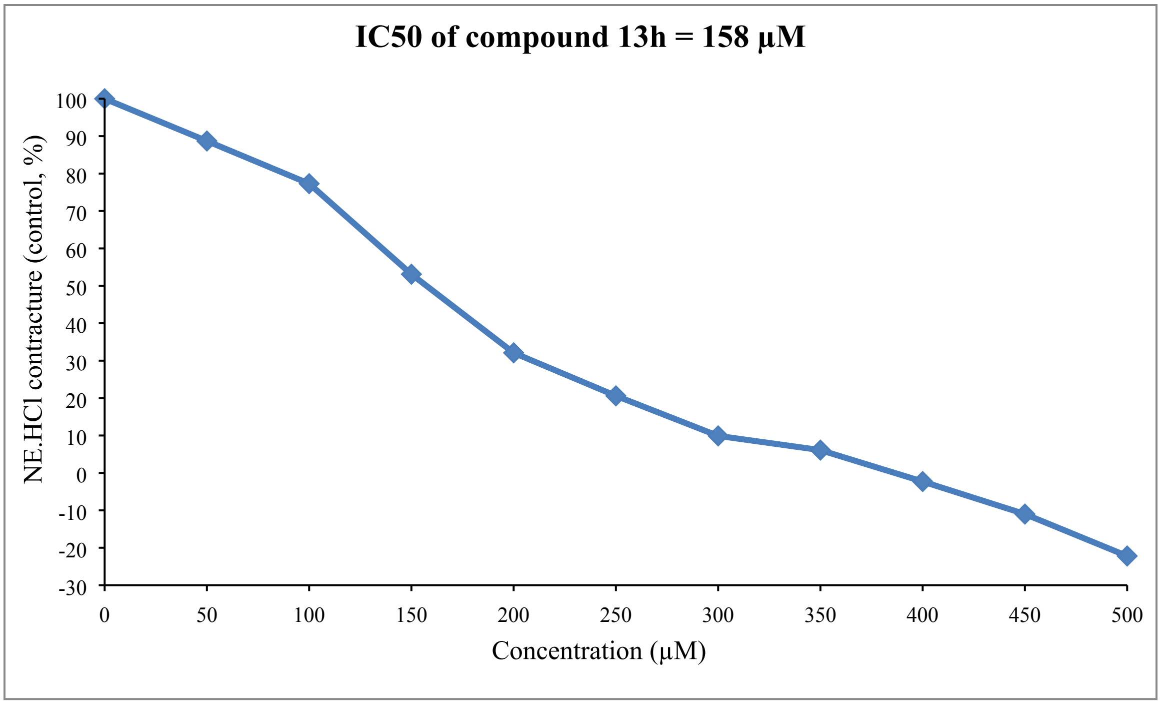
IC50 of compound 13h = 158 µM
NE.HCl contracture (control, %)
100
90
80
70
60
50
40
30
20
10
0
-10
-20
-30
0
50
100
150
200
250
300
350
400
450
500
Concentration (µM)

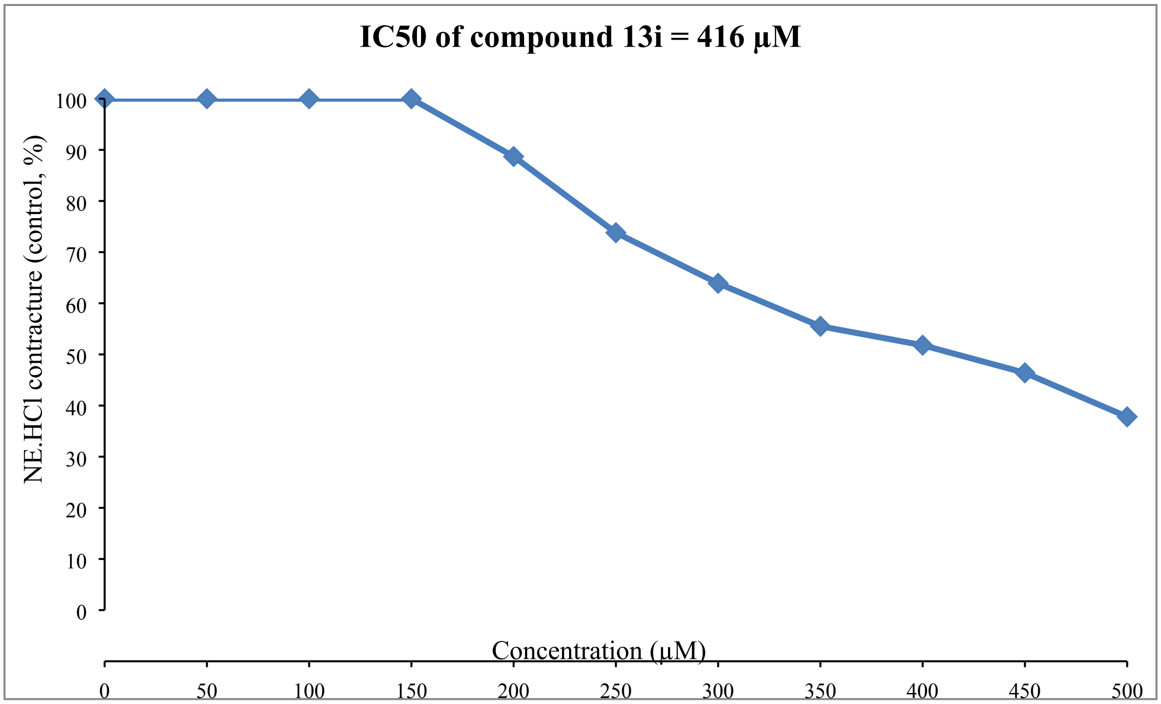
IC50 of compound 13i = 416 µM
NE.HCl contracture (control, %)
100
90
80
70
60
50
40
30
20
10
0
Concentration (µM)
0
50
100
150
200
250
300
350
400
450
500

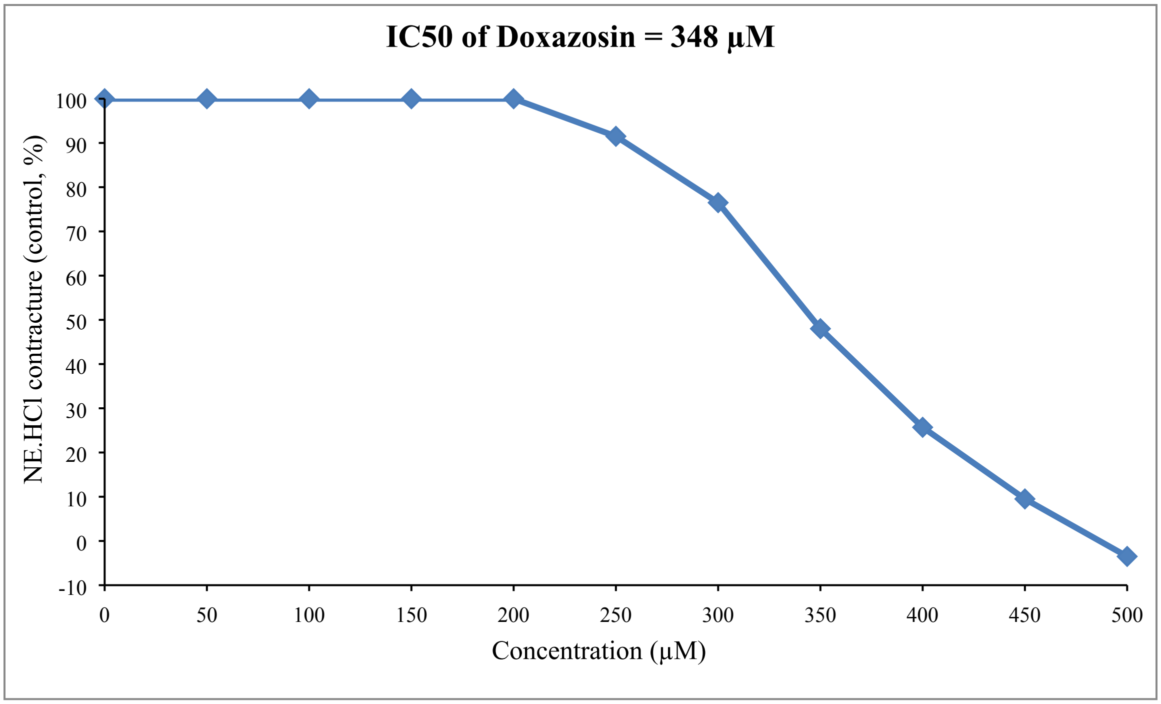

Fig. S33. Effect of synthesized compounds (**10c**, **13a-i**) and Doxazosin on contracture induced by norepinephrine hydrochloride (NE.HCl) in rat thoracic aortic rings.

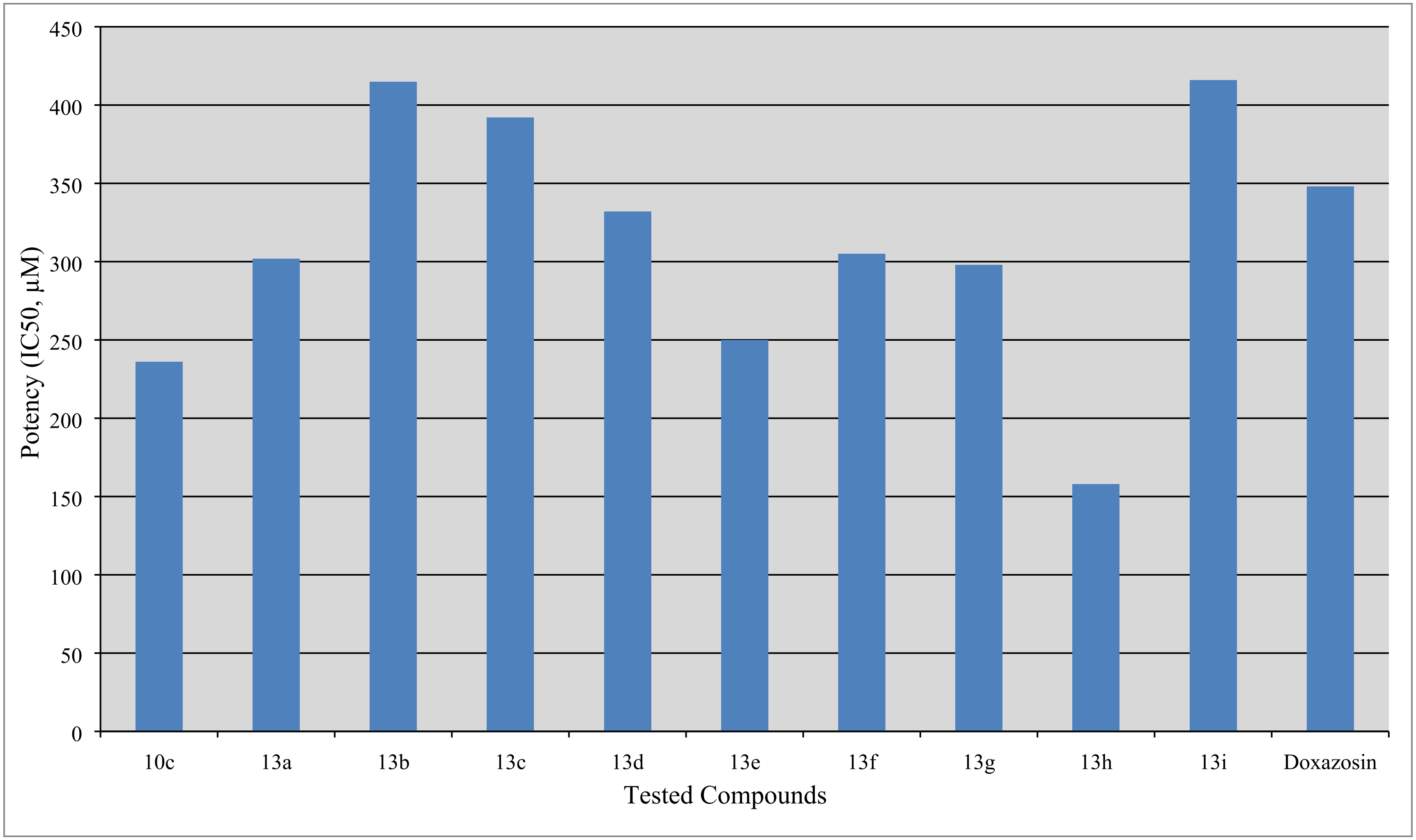

Fig. S34. Potency (IC_{50}, µM) of the tested compounds on contracture induced by norepinephrine hydrochloride in rat thoracic aortic rings compared with Doxazosin used as a reference standard.

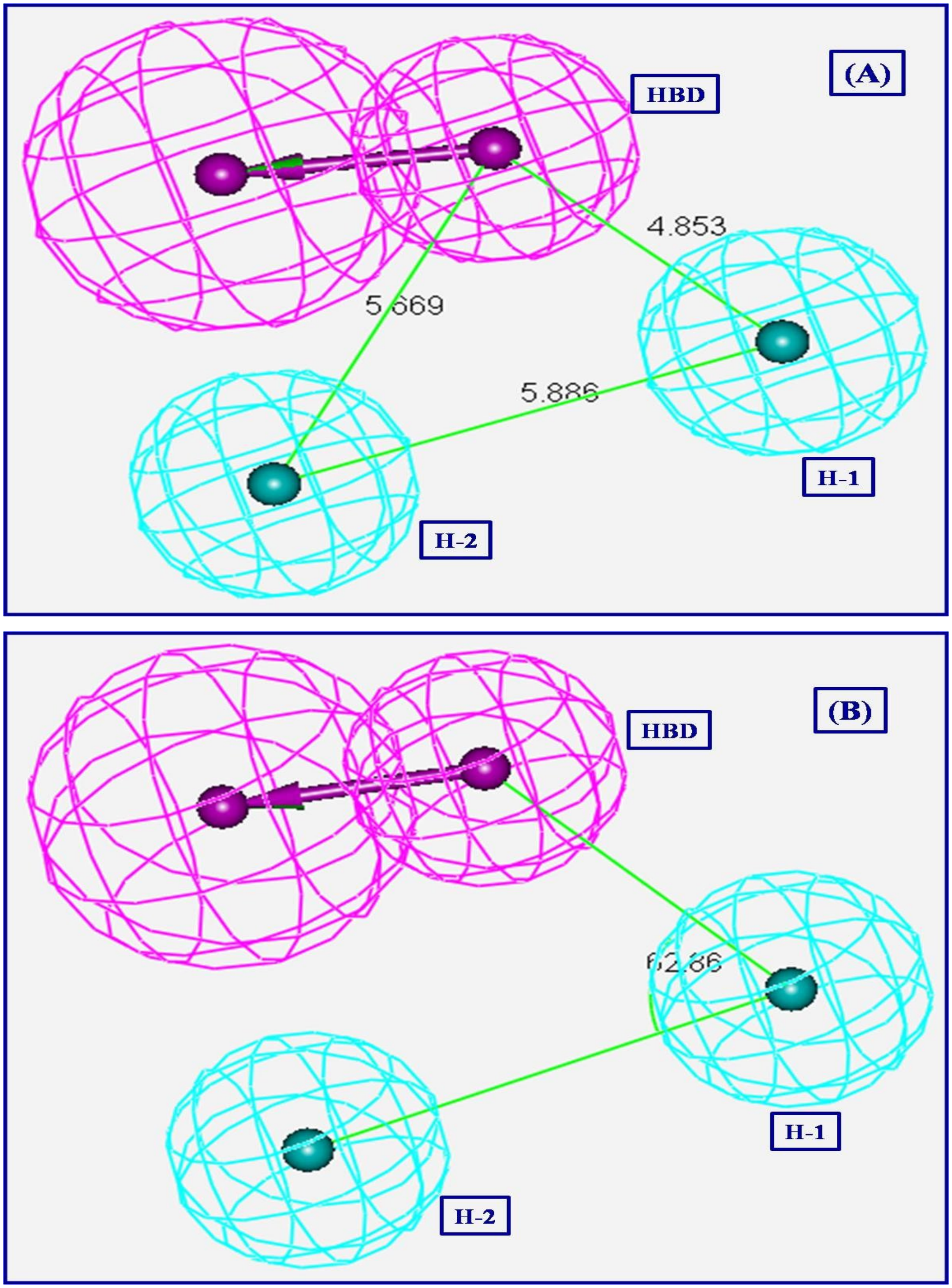

Fig. S35. (A) Constraint distances “H-1 – H-2 = 5.886, H-1 – HBD = 4.853, H-2 – HBD = 5.669 Å”, (B) Constraint angle “H-2 – H-2 – HBD = 62.86 °” of the generated 3D-pharmacophore for the tested quinazoline-4-carboxamides **13a–i** which contains two hydrophobics (H-1 and H-2; light blue) and one hydrogen bonding donor (HBD; purple).

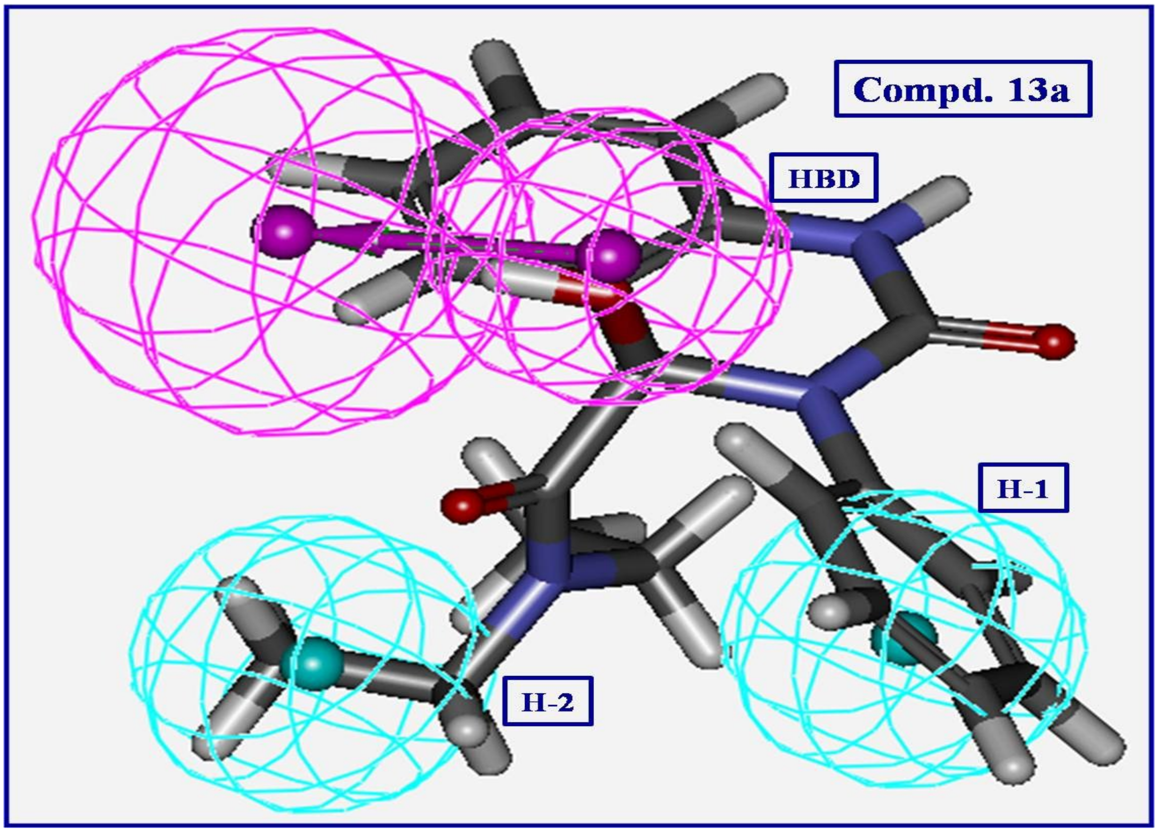
Compd. 13a
HBD
H-1
H-2

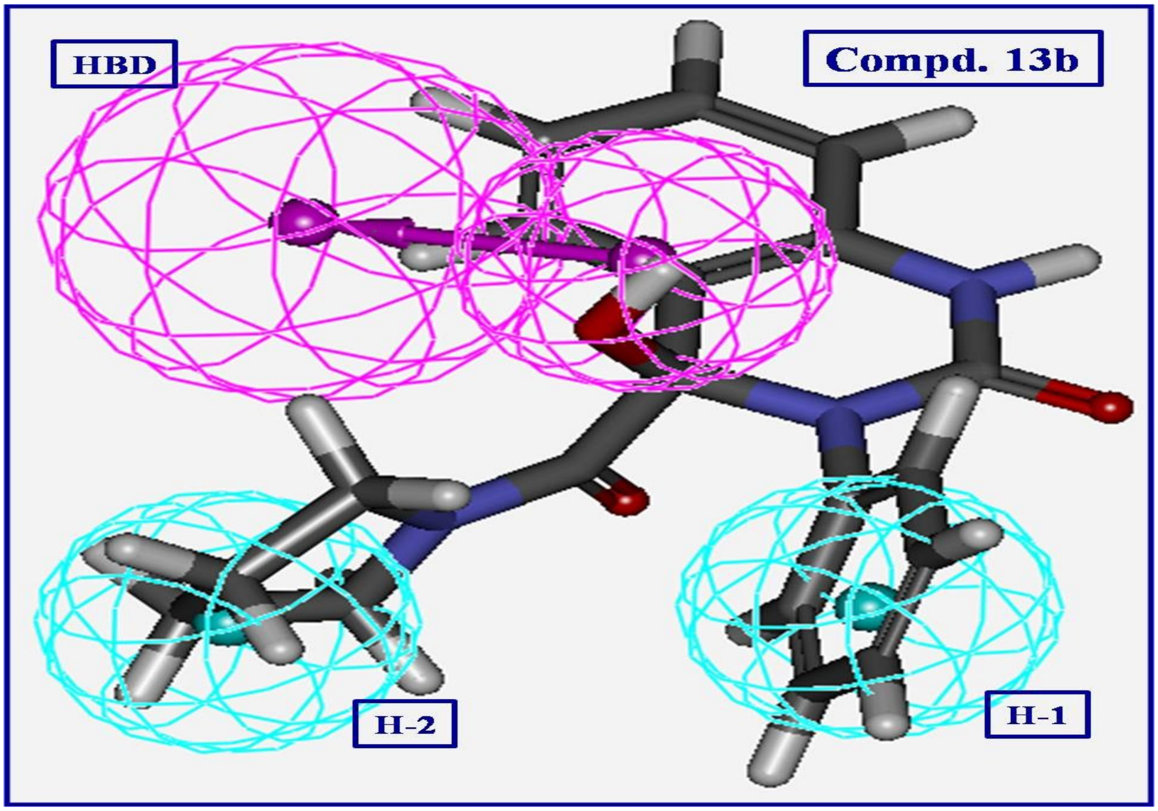
HBD
Compd. 13b
H-2
H-1

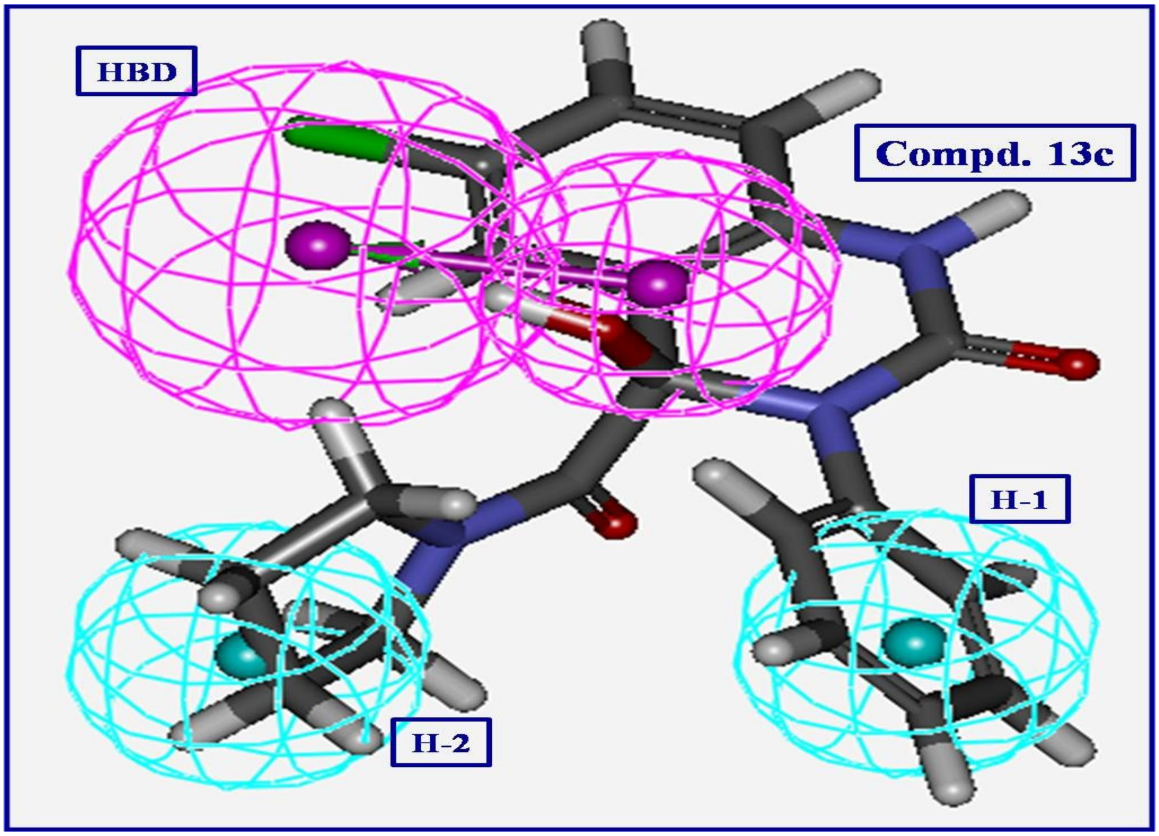
HBD
Compd. 13c
H-1
H-2

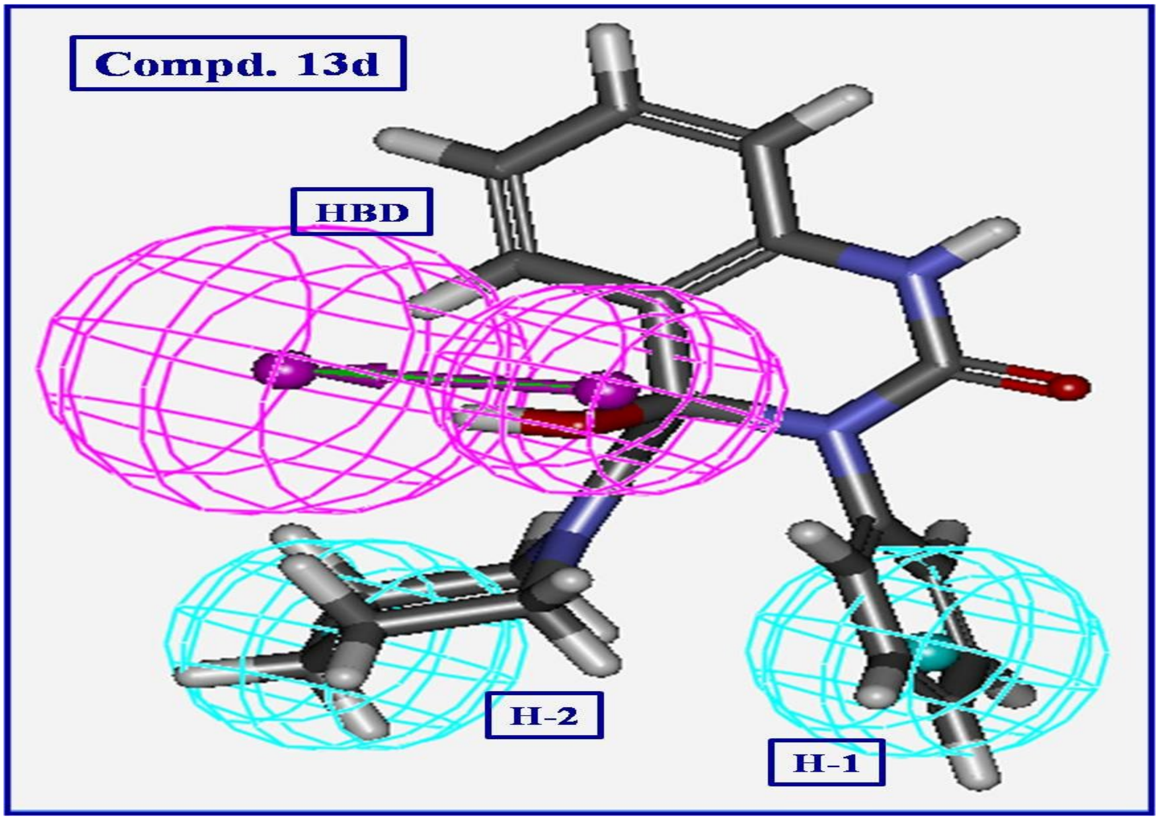
Compd. 13d
HBD
H-2
H-1

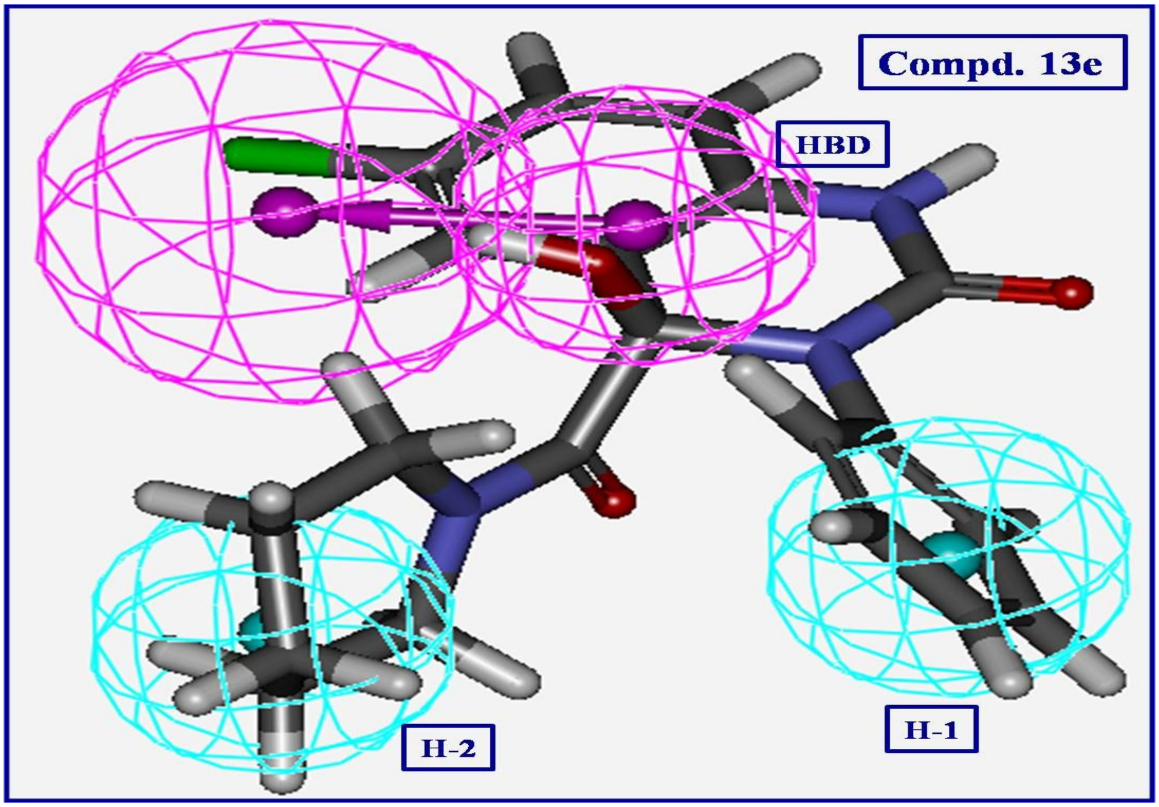
Compd. 13e
HBD
H-2
H-1

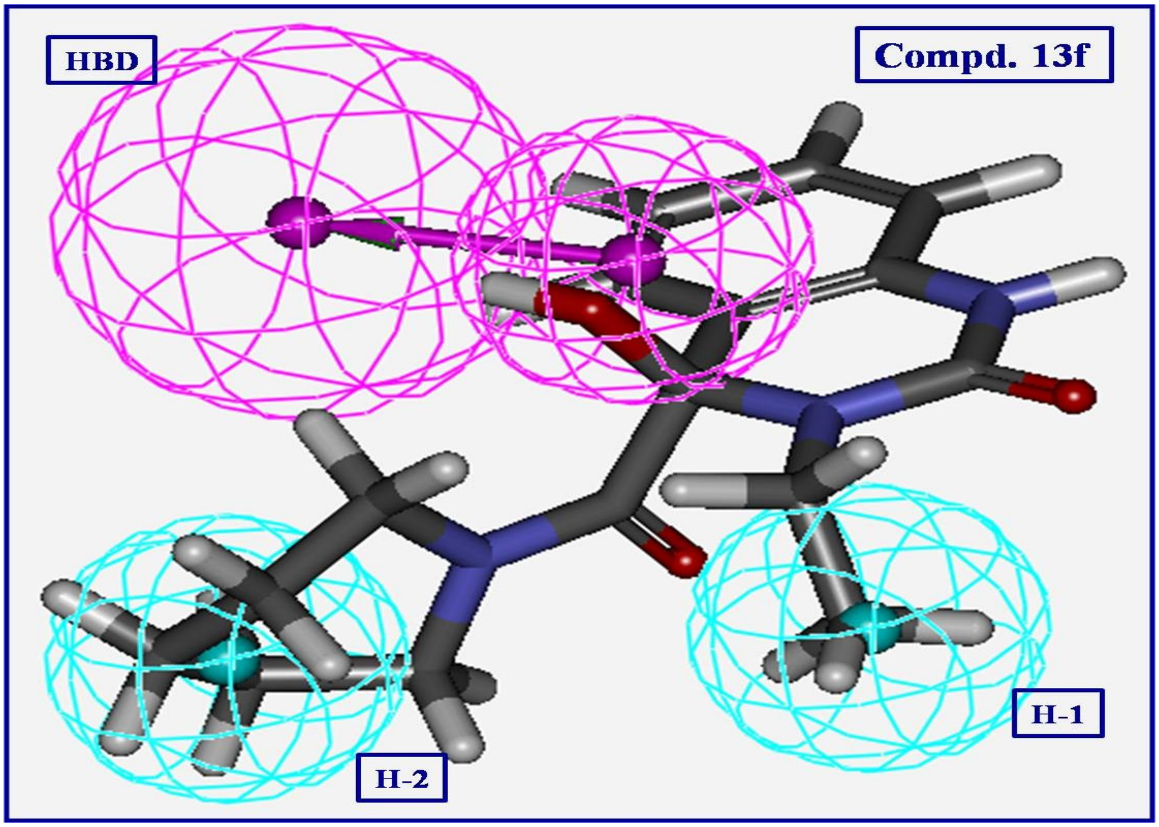
HBD
Compd. 13f
H-1
H-2

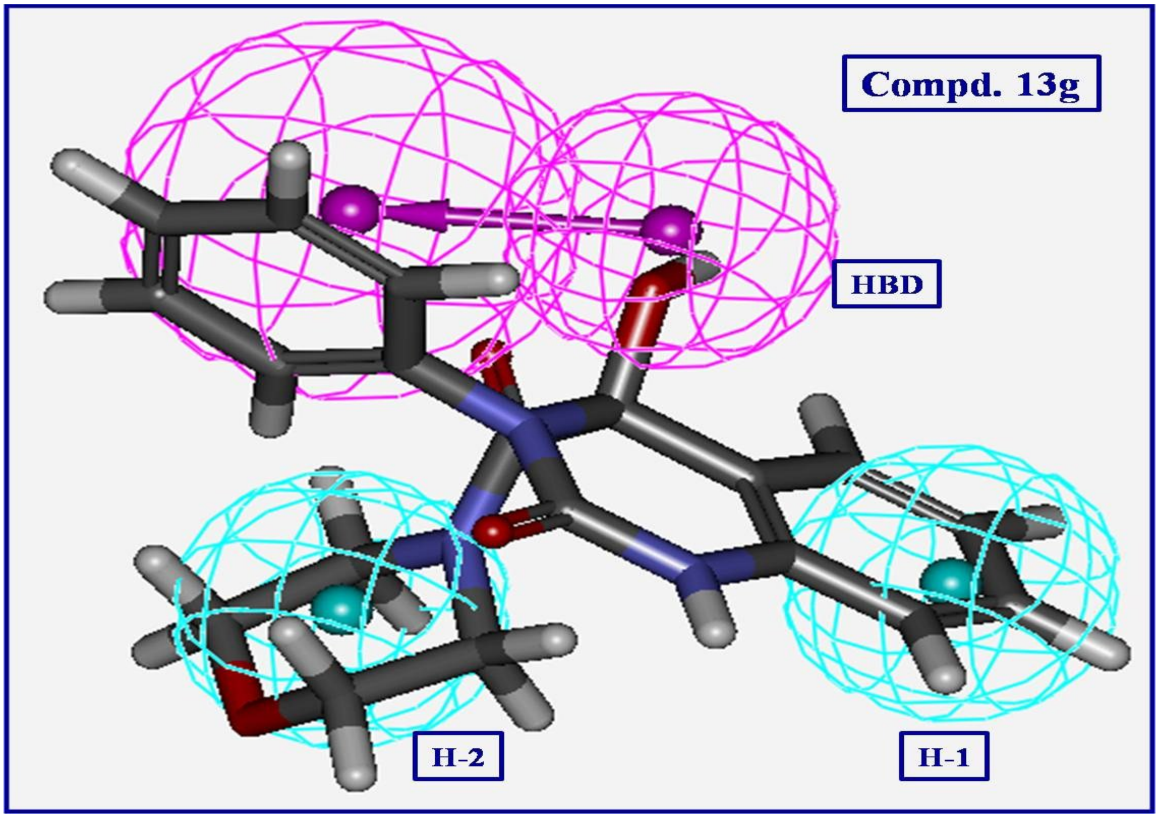
Compd. 13g
HBD
H-2
H-1

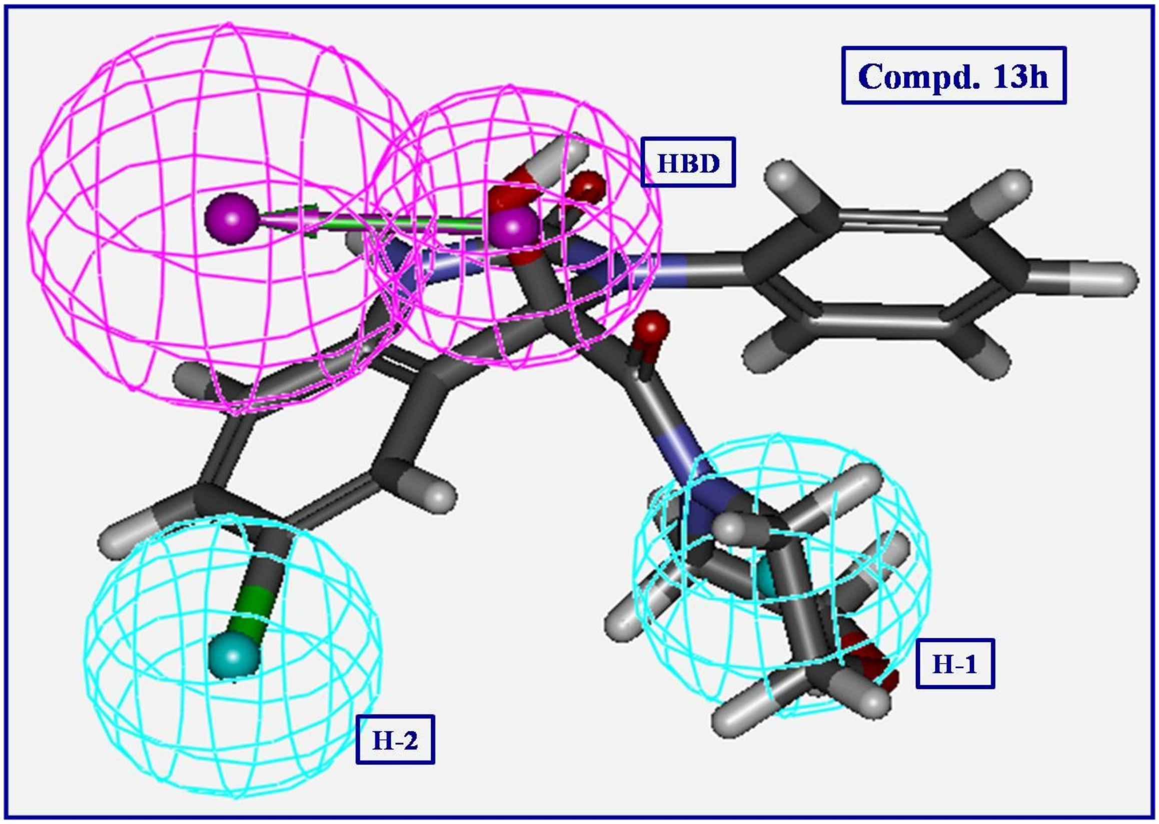
Compd. 13h
HBD
H-1
H-2

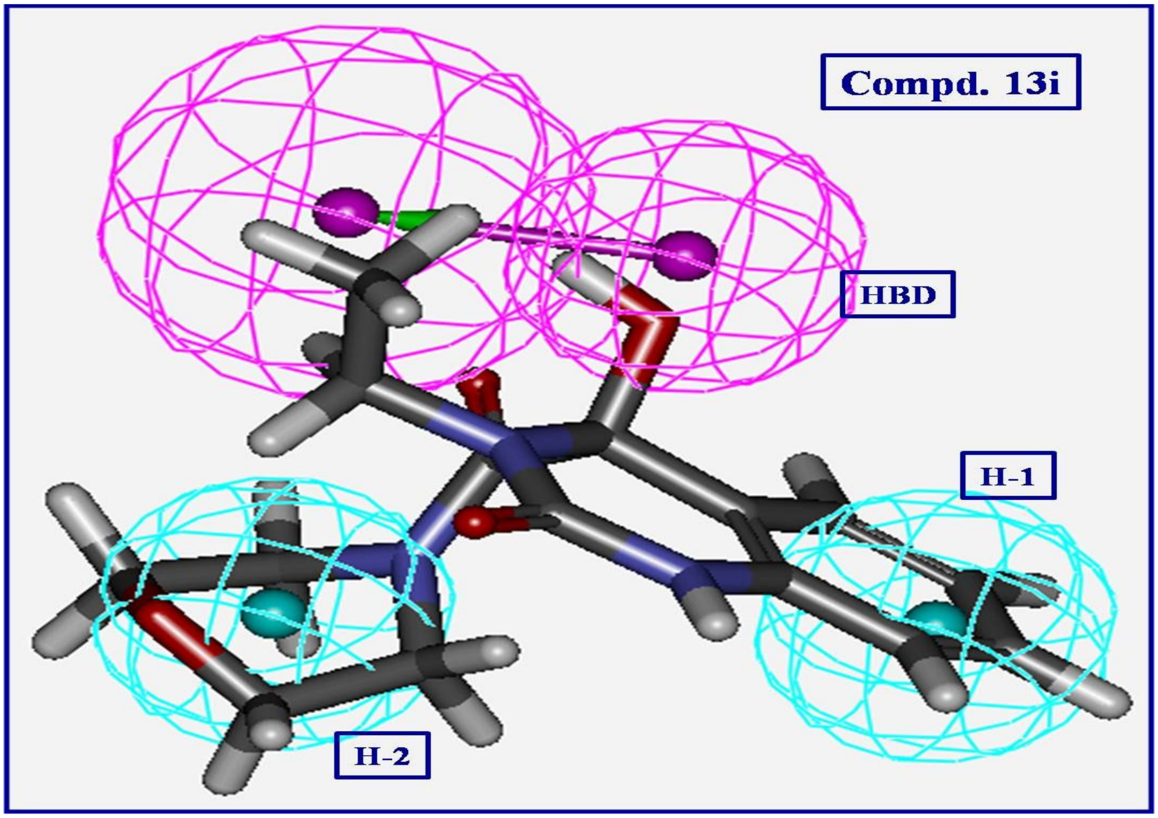

Fig. S36. 3D-pharmacophore model mapped on the tested quinazoline-4-carboxamides **13a–i**.
